# Modified unlocked nucleic acid (MUNA) mitigates off-target effects of small interfering RNAs

**DOI:** 10.1093/nar/gkaf937

**Published:** 2025-09-29

**Authors:** Shohei Mori, Dhrubajyoti Datta, Lydia Perkins, Michelle Jung, Lauren Blair Woods, Alex Eaton, June Qin, Tim Racie, MaryBeth Kim, Dale C Guenther, Adam Castoreno, Mark K Schlegel, Klaus Charisse, Martin Egli, Shigeo Matsuda, Muthiah Manoharan

**Affiliations:** Alnylam Pharmaceuticals, 675 West Kendall, Cambridge, MA 02142, United States; Alnylam Pharmaceuticals, 675 West Kendall, Cambridge, MA 02142, United States; Alnylam Pharmaceuticals, 675 West Kendall, Cambridge, MA 02142, United States; Alnylam Pharmaceuticals, 675 West Kendall, Cambridge, MA 02142, United States; Alnylam Pharmaceuticals, 675 West Kendall, Cambridge, MA 02142, United States; Alnylam Pharmaceuticals, 675 West Kendall, Cambridge, MA 02142, United States; Alnylam Pharmaceuticals, 675 West Kendall, Cambridge, MA 02142, United States; Alnylam Pharmaceuticals, 675 West Kendall, Cambridge, MA 02142, United States; Alnylam Pharmaceuticals, 675 West Kendall, Cambridge, MA 02142, United States; Alnylam Pharmaceuticals, 675 West Kendall, Cambridge, MA 02142, United States; Alnylam Pharmaceuticals, 675 West Kendall, Cambridge, MA 02142, United States; Alnylam Pharmaceuticals, 675 West Kendall, Cambridge, MA 02142, United States; Alnylam Pharmaceuticals, 675 West Kendall, Cambridge, MA 02142, United States; Department of Biochemistry and Center for Structural Biology, Vanderbilt University, School of Medicine, Nashville, TN 37232-0146, United States; Alnylam Pharmaceuticals, 675 West Kendall, Cambridge, MA 02142, United States; Alnylam Pharmaceuticals, 675 West Kendall, Cambridge, MA 02142, United States

## Abstract

Unlocked nucleic acid (UNA) is a nucleic acid analog that has an acyclic ribose ring lacking the bond between C2′ and C3′ atoms. The base-pairing properties of UNA have been studied, and it has been used as a scaffold for conjugation, but the chemical space around UNA and its potential applications in the context of short interfering RNAs (siRNAs), which mediate RNA interference, have not been thoroughly explored. In this study, we report syntheses of methylated and methoxylated UNAs and their incorporation into siRNAs. siRNAs with 5′-(*R*)-methyl-UNA and with 5′-(*S*)-methyl-UNA in the seed region had comparable potencies but reduced off-target effects compared to siRNA modified with UNA. In mice, siRNAs with modified UNAs were of comparable potency to an siRNA of the same sequence and chemistry lacking UNA. Modeling studies indicated that the flexibilities of UNA and the modified UNAs facilitate kinking of the antisense strand when incorporated at position 7. These findings highlight the potential of modified UNA for advancing therapeutics that act through the RNA interference pathway.

## Graphical Abstract

**Graphical Abstract:**
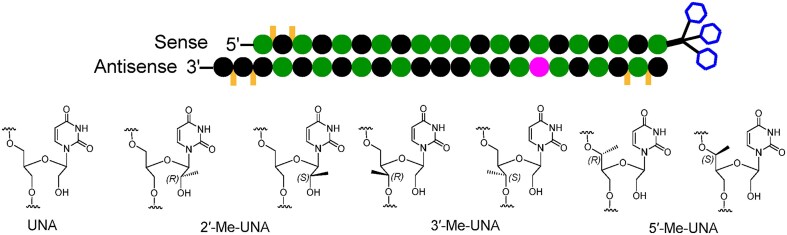

## Introduction

Oligonucleotide therapeutics, particularly agents that act through the RNA interference (RNAi) pathway, hold remarkable potential for addressing unmet medical needs. Seven RNAi-based therapeutics have been clinically approved: patisiran (ONPATTRO), givosiran (GIVLAARI), lumasiran (OXLUMO), inclisiran (LEQVIO), vutrisiran (AMVUTTRA), nedosiran (Rivfloza), and fitusiran (QFITLIA) [1–12]. These small interfering RNAs (siRNAs) mediate gene silencing by acting post-transcriptionally. When loaded onto AGO2, the catalytic component of the RNA-induced silencing complex (RISC), siRNAs target complementary mRNAs for degradation, thereby decreasing the expression of the encoded, undesired disease-causing proteins [13].

**Figure 1.**
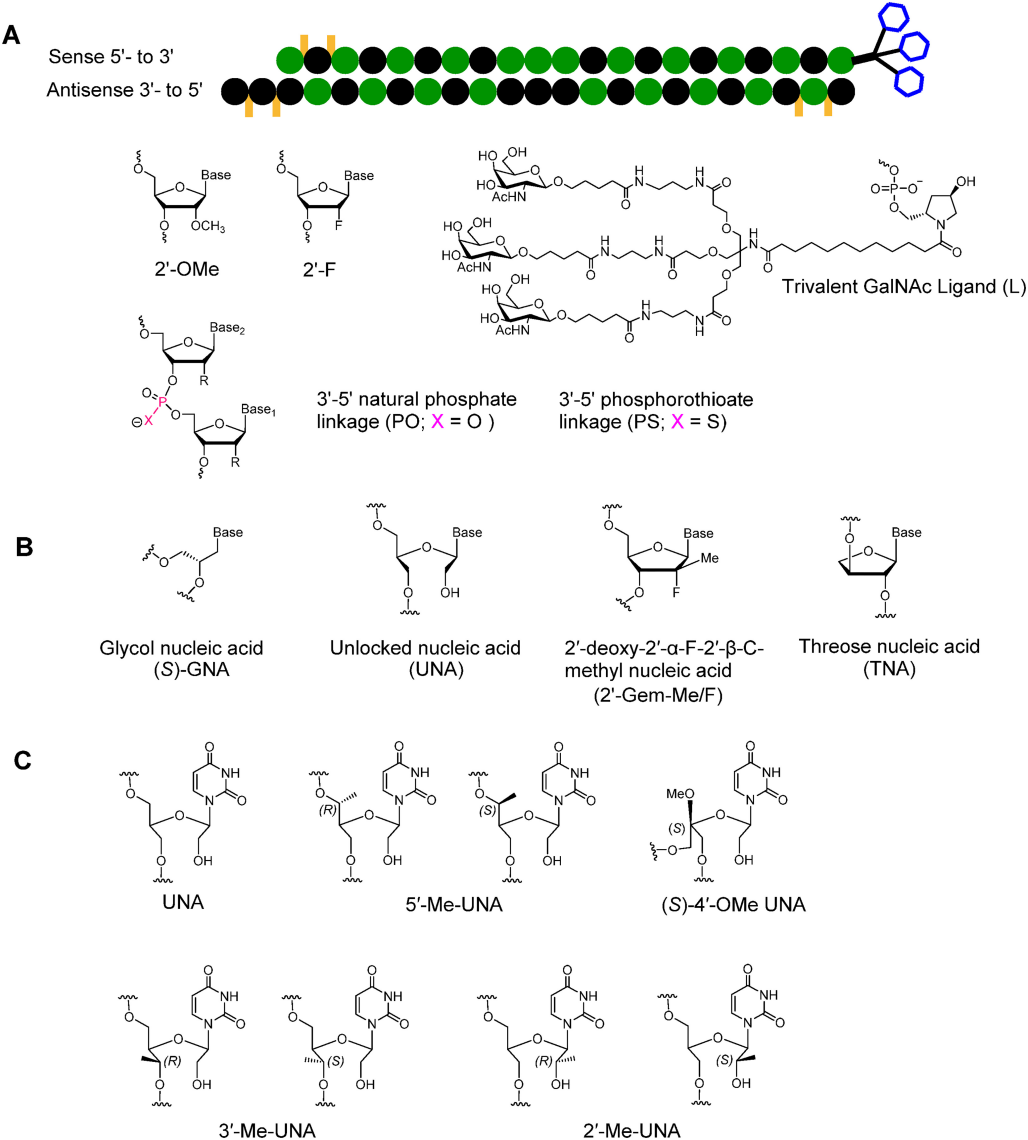
Chemical modifications used in siRNAs. (**A**) Chemical modifications used in siRNAs approved for clinical use. (**B**) Chemical modifications used to mitigate off-target effects. (**C**) Chemical modifications used in this study. Both *R-* and *S*-isomers of 2′-, 3′-, and 5′-Me-UNA and 4′-(*S*)-OMe-UNA were studied.

The successes of these therapeutics hinge on chemical modifications and efficient delivery methods [13–15]. Natural RNA duplexes are metabolically unstable, and their use therapeutically necessitates the inclusion of chemically modified building blocks to minimize enzymatic degradation, enhance lipophilicity, improve cell-membrane permeability, and mitigate immune responses and off-target effects (Fig. 1A) [16]. Patisiran has 2′-*O*-methyl (2′-OMe) ribonucleotides and is formulated in lipid nanoparticles [1]. Other therapeutics from Alnylam, givosiran, lumasiran, inclisiran, vutrisiran, and fitusiran are chemically modified with both 2′-OMe and 2′-deoxy-2′-fluoro (2′-F) ribonucleotides and are conjugated to a trivalent *N*-acetylgalactosamine (GalNAc) [17–19]. This GalNAc ligand specifically binds to the hepatic asialoglycoprotein receptor to mediate liver cell-specific uptake of the siRNAs. All clinically used siRNAs also have phosphorothioate (PS) linkages at the required 5′ and 3′ termini, which provide metabolic stability [20–23].

The 2′-OMe and 2′-F modifications help to maintain the RNA-like C3′-endo conformation, resulting in improved binding to complementary mRNA, reduced immune activation, more favorable AGO2 interactions, and increased resistance to nuclease degradation. Thermally destabilizing modifications, like (*S*)-glycol nucleic acid ((*S*)-GNA), α-(L)-threofuranosyl nucleic acid (TNA), and 2′-deoxy-2′-α-F-2′-β-*C*-methyl nucleotide (2′-*geminal*-Me/F) can minimize off-target effects (Fig. 1B) [18, 24–27]. (*S*)-GNA is well accommodated within the RNA duplex, allowing its incorporation without altering the overall structure. When incorporated into the seed region of the antisense strand of an siRNA, (*S*)-GNA decreases binding affinity for mRNAs that do not have complete complementarity to the siRNA antisense strand, without altering desired on-target silencing efficacy [28, 29]. TNA placed at position 7 of the antisense strand of the siRNA also mitigates off-target effects, likely due to the decrease in the thermodynamic binding affinity relative to the 2′-O-Me residue [27]. The 2′-Gem modifications also cause significant thermal destabilization due to steric effects and mitigate off-target effects [23]. When bound to AGO2, the antisense strand is kinked between positions 6 and 7, and the short internucleotide linkages of GNA and TNA contribute to the proper placement of these modifications within the RISC [27–29].

Another thermally destabilizing modification, unlocked nucleic acid (UNA, Fig. 1B), invented by Wengel *et al.* [30] has flexibility due to the lack of the C2′-C3′ bond, the bond that closes the ribose ring in natural nucleotides. Incorporation of UNA residues decreases RNA duplex thermal stability by 5–10°C [25, 30, 31]. UNA modifications do not disrupt the helical RNA duplex structure and protect siRNAs from degradation [32]. Previous work has shown that UNAs at specific positions enhance siRNA potency by preventing loading of the sense strand into the RISC, thus minimizing off-target effects [25, 33–35]. Here, we explored modified UNAs methylated at various positions of the acyclic sugar moiety (Fig. 1C). These methylated and methoxylated UNAs improve the resistance of siRNA against degradation by nucleases, and siRNAs with these modifications in the seed region do not decrease potency but do mitigate off-target effects, making them promising for RNAi-based therapeutic applications.

## Materials and methods

### Synthetic procedures and compound characterization

*Synthesis of compound* **3S**: To a solution of compound **1** synthesized, as described [36] (20.0 g, 42.3 mmol) in anhydrous dichloromethane (DCM, 500 mL) cooled to 0°C, was added Dess-Martin periodinane (21.5 g, 50.8 mmol, 1.2 equiv.). The reaction was stirred at 0°C for 1 h, warmed to room temperature, and stirred for additional 1.5 h. The reaction was then quenched by slowly adding the reaction mixture to vigorously stirred 10% aq. Na_2_S_2_O_3_ (300 mL) and saturated aq. NaHCO_3_ (300 mL) at 0°C and stirred for 1 h. After quenching, the organic layer was extracted with DCM (3 × 100 mL), dried over anhydrous Na_2_SO_4_, filtered, and concentrated under reduced pressure to yield crude compound **2** as flaky white solid. The dried compound **2** was resuspended in anhydrous THF (300 mL), and the solution was added via cannula to a stirring solution of Me_3_Al (2 M in toluene, 63.5 mL, 126.9 mmol, 3 equiv.) in THF (300 mL) at 0°C. After stirring at 0°C for 1 h, the reaction was warmed to room temperature and allowed to stir overnight. The reaction was brought to 0°C and quenched by gradually adding a 1:1 solution of aq. H_3_PO_4_ (10%) and saturated aq. NH_4_Cl (20 mL). After the solvent was removed under reduced pressure, the crude residue was extracted with DCM and sat. NaHCO_3_. The organic layer was separated, washed with brine, dried over anhydrous Na_2_SO_4_, filtered, and concentrated under reduced pressure. The crude material was purified by flash silica gel column chromatography (0–30% EtOAc in hexanes) to obtain compound **3S** as a white foam (5.85 g, 12.02 mmol, 25%; R_f_ = 0.48, developed with 50% EtOAc in hexane). ^1^H NMR (400 MHz, DMSO-*d*_6_) δ 11.35 (s, 1H), 8.07 (d, *J* = 8.1 Hz, 1H), 5.83 (d, *J* = 6.0 Hz, 1H), 5.71 (d, *J* = 8.1 Hz, 1H), 5.27 (d, *J* = 4.2 Hz, 1H), 4.26 (dd, *J* = 6.1, 4.4 Hz, 1H), 4.12 (dd, *J* = 4.5, 2.5 Hz, 1H), 3.88 – 3.78 (m, 1H), 3.72 (t, *J* = 2.2 Hz, 1H), 1.14 (d, *J* = 6.4 Hz, 3H), 0.89 (s, 9H), 0.83 (s, 9H), 0.08 (d, *J* = 3.6 Hz, 6H), and −0.04 (s, 3H) ppm. ^13^C NMR (101 MHz, DMSO-*d*_6_) δ 162.94, 150.74, 140.17, 102.05, 88.54, 86.43, 74.75, 72.90, 65.04, 25.70, 25.59, 19.98, 17.74, 17.60, −4.63, −4.77, −4.84, and −5.07 ppm. HRMS: [M + H]^+^ calc. for C_22_H_43_N_2_O_6_Si_2_, 487.2654; found: 487.2659.

*Synthesis of compound* **4S**: To a solution of compound **3S** (5.85 g, 12.02 mmol) in anhydrous THF (50 mL) and anhydrous pyridine (10 mL) were added DMTrCl (12.2 g, 36.1 mmol, 3 equiv.) and AgNO_3_ (4.08 g, 24.0 mmol, 2 equiv.). The mixture was stirred at room temperature for 24 h then additional DMTrCl (6.11 g, 18.0 mmol, 1.5 equiv.) and AgNO_3_ (2.04 g, 12.0 mmol, 1.0 equiv.) were added to the reaction mixture. The mixture was stirred at room temperature overnight. The mixture was filtered over Celite, and the filter cake was washed with DCM. The filtrate was concentrated under reduced pressure, and the residue was extracted with DCM and saturated aq. NaHCO_3_. The organic layer was washed with brine, dried over anhydrous Na_2_SO_4_, filtered and concentrated. The crude residue was purified by flash column chromatography (0–25% EtOAc in hexanes) to obtain the DMTr-protected compound as a yellow foam (8.05 g, 10.2 mmol, 85%, R_f_ = 0.35, developed with 33% EtOAc in hexanes). HRMS: [M + Na]^+^ calc. for C_43_H_60_N_2_O_8_Si_2_Na, 811.3786; found: 811.3798.

To a solution of the DMTr-protected compound (8.05 g, 10.2 mmol) in THF (51 mL) was added TBAF (1 M in THF, 25.5 mL, 25.5 mmol, 2.2 equiv.). After stirring at room temperature overnight, the solvent was removed under reduced pressure. The crude residue was purified by flash column chromatography (0–100% EtOAc in hexanes then 2.5% MeOH in EtOAc) to obtain compound **4S** as a yellowish foam (5.84 g, quant.; R_f_ = 0.50, developed with 5% MeOH in EtOAc). ^1^H NMR (500 MHz, DMSO-*d*_6_) δ 11.41–11.34 (m, 1H), 7.57 (d, *J* = 8.1 Hz, 1H), 7.46–7.40 (m, 2H), 7.30 (t, *J* = 7.9 Hz, 6H), 7.21 (t, *J* = 7.3 Hz, 1H), 6.88 (dd, *J* = 8.8, 6.7 Hz, 4H), 5.68 (d, *J* = 5.1 Hz, 1H), 5.58 (dd, *J* = 8.0, 1.6 Hz, 1H), 5.39 (d, *J* = 5.2 Hz, 1H), 5.03 (d, *J* = 5.1 Hz, 1H), 4.01 (h, *J* = 5.8 Hz, 2H), 3.74 (s, 3H), 3.72 (s, 4H), 3.61–3.51 (m, 1H), 0.67 (d, *J* = 6.2 Hz, 3H) ppm. ^13^C NMR (126 MHz, DMSO-*d*_6_) δ 162.93, 158.06, 150.67, 146.11, 140.47, 136.40, 136.30, 130.16, 130.08, 127.95, 127.61, 126.61, 112.99, 101.93, 87.25, 86.43, 85.81, 72.56, 69.24, 68.89, 55.00, 54.99, and 17.09 ppm. HRMS: [M + Na]^+^ calc. for C_31_H_32_N_2_O_8_Na, 583.2051; found: 583.2068.

*Synthesis of compound* **5S**: To a solution of compound **4S** (5.84 g, 10.42 mmol) in dioxane (135 mL) and H_2_O (25 mL) was added NaIO_4_ (2.45 g, 11.46 mmol, 1.1 equiv.) dissolved in H_2_O (25 mL). The bi-layer reaction mixture was vigorously stirred at room temperature for 4 h. The reaction mixture was filtered through a sintered funnel, and the filter cake was washed with additional dioxane. To the filtrate was added NaBH_4_ (0.434 g, 11.46 mmol, 1.1 equiv.). After stirring at room temperature for 2 h, the mixture was cooled to 0°C then quenched with 1:1 v/v acetic acid:pyridine. After the solvent was removed under reduced pressure, the crude residue was extracted with EtOAc and saturated aq. NaHCO_3_. The organic layer was separated, dried over anhydrous Na_2_SO_4_, filtered, and concentrated under reduced pressure. The crude material was purified by flash column chromatography (0–5% MeOH in DCM) to obtain compound **5S** as a white foam (5.01 g, 8.90 mmol, 85%; R_f_ = 0.13 developed with 5% MeOH in DCM). ^1^H NMR (400 MHz, DMSO-*d*_6_) δ 11.14 (d, *J* = 2.2 Hz, 1H), 7.35 (dd, *J* = 8.0, 1.8 Hz, 3H), 7.30–7.15 (m, 7H), 6.90–6.78 (m, 4H), 5.64 (dd, *J* = 6.4, 4.7 Hz, 1H), 5.47 (dd, *J* = 8.0, 2.2 Hz, 1H), 5.01 (t, *J* = 6.0 Hz, 1H), 4.68 (t, *J* = 5.4 Hz, 1H), 3.82 (dd, *J* = 11.7, 5.7, 2.2 Hz, 1H), 3.60–3.51 (m, 2H), 3.50–3.39 (m, 2H), 3.24–3.17 (m, 1H), 3.12–3.01 (m, 1H), 2.07 (s, 2H), 1.19 (t, *J* = 7.3 Hz, 2H), 0.56 (d, *J* = 6.3 Hz, 3H) ppm. ^13^C NMR (126 MHz, DMSO-*d*_6_) δ 163.08, 158.09, 158.03, 150.80, 145.64, 140.57, 136.49, 136.11, 129.75, 129.60, 127.65, 127.62, 126.68, 113.02, 101.18, 85.80, 84.73, 81.69, 68.82, 61.24, 60.36, 54.98, 45.48, 39.24, 15.38, 8.49, and 1.12 ppm. HRMS: [M + Na]^+^ calc. for C_31_H_34_N_2_O_8_Na, 585.2207; found: 585.2224.

*Synthesis of compound* **6S**: To a solution of compound **5S** (5.01 g, 8.90 mmol) in anhydrous DCM (245 mL) and pyridine (7 mL) cooled to −78°C was slowly added benzoyl chloride (1.14 mL, 9.79 mmol, 1.1 equiv.). After stirring at −78°C for 1 h, the reaction mixture was brought to 0°C, and quenched with ethanol (5 mL). The mixture was extracted with DCM and saturated aq. NaHCO_3_. The organic layer was washed with brine, separated, dried over anhydrous Na_2_SO_4_, filtered, and concentrated. The crude residue was purified by flash column chromatography (0–75% EtOAc in hexanes) to yield compound **6S** as a white foam (1.31 g, 1.96 mmol, 22%; R_f_ = 0.32, developed with 50% EtOAc in hexanes). ^1^H NMR (400 MHz, DMSO-*d*_6_) δ 11.29–11.25 (m, 1H), 7.87 (dd, *J* = 8.4, 1.4 Hz, 2H), 7.69–7.61 (m, 1H), 7.55–7.46 (m, 3H), 7.39–7.32 (m, 2H), 7.27 (d, *J* = 1.0 Hz, 1H), 7.25 (d, *J* = 1.7 Hz, 2H), 7.23 (d, *J* = 2.7 Hz, 3H), 7.22 (d, *J* = 2.1 Hz, 1H), 7.20 (t, *J* = 1.4 Hz, 1H), 6.03 (dd, *J* = 6.8, 5.0 Hz, 1H), 5.57–5.52 (m, 1H), 4.78 (t, *J* = 5.3 Hz, 1H), 4.50 (dd, *J* = 11.5, 5.0 Hz, 1H), 4.33 (dd, *J* = 11.5, 6.8 Hz, 1H), 3.84 (ddd, *J* = 11.6, 5.1, 2.2 Hz, 1H), 3.72 (s, 7H), 3.59 (ddd, *J* = 11.6, 8.4, 5.5 Hz, 1H), 3.54–3.47 (m, 1H), 3.12 (ddd, *J* = 8.5, 4.6, 2.2 Hz, 1H), 0.69 (d, *J* = 6.3 Hz, 3H) ppm. ^13^C NMR (101 MHz, DMSO-*d*_6_) δ 164.93, 162.94, 158.12, 158.06, 150.58, 145.59, 139.93, 136.40, 136.10, 133.55, 129.74, 129.62, 129.12, 129.01, 128.77, 127.68, 127.64, 126.71, 113.03, 101.88, 85.88, 81.76, 81.60, 68.74, 63.36, 60.42, 54.98, 39.40, 39.18, 38.97, and 15.50 ppm. HRMS: [M + Na]^+^ calc. for C_38_H_38_N_2_O_9_Na, 689.2470; found: 689.2465.

*Synthesis of compound* **7S**: To a solution of compound **6S** (1.21 g, 1.81 mmol) in DCM (10 mL) and DIPEA (0.66 mL, 5.4 mmol) was added 2-cyanoethyl-*N,N*-diisopropylchlorophosphoramidite (0.695 mL, 2.2 mmol) at 0°C. The mixture was stirred at 0°C for 2 h. The reaction mixture was diluted with DCM (100 mL) then washed with saturated aq. NaHCO_3_ (100 mL). The organic layer was separated, dried over anhydrous Na_2_SO_4_, filtered, and concentrated. The amidite **7S** was precipitated from hexanes (1.33 g, 1.5 mmol, 85%). ^1^H NMR (600 MHz, CD_3_CN) δ 9.03 (s, 1H), 7.96–7.91 (m, 2H), 7.61 (tq, *J* = 7.4, 1.3 Hz, 1H), 7.50–7.40 (m, 4H), 7.37–7.14 (m, 9H), 6.81 (qdd, *J* = 8.0, 6.6, 3.6 Hz, 4H), 6.02 (q, *J* = 5.3 Hz, 1H), 5.55 (dd, *J* = 8.1, 4.5 Hz, 1H), 4.42–4.33 (m, 1H), 4.27 (dt, *J* = 11.5, 5.6 Hz, 1H), 4.11–4.00 (m, 2H), 3.87–3.69 (m, 9H), 3.66–3.53 (m, 2H), 3.01–2.85 (m, 1H), 2.66–2.58 (m, 2H), 1.18–1.13 (m, 12H), 0.95 (dd, *J* = 15.0, 6.4 Hz, 3H) ppm. ^13^C NMR (151 MHz, CD_3_CN) δ 166.41, 166.39, 163.83, 159.74, 159.70, 151.37, 151.33, 147.06, 147.01, 140.73, 140.70, 137.75, 137.66, 137.31, 137.30, 134.39, 134.38, 131.18, 131.16, 131.01, 130.99, 130.47, 130.45, 130.43, 129.61, 128.88, 128.75, 128.74, 127.85, 119.53, 114.02, 114.01, 113.99, 102.91, 87.46, 83.00, 82.92, 81.14, 81.08, 80.99, 80.94, 70.06, 64.65, 64.60, 64.53, 64.42, 64.08, 63.98, 60.95, 59.65, 59.53, 59.40, 55.89, 55.88, 43.89, 43.81, 25.09, 25.04, 25.02, 24.97, 24.94, 24.92, 24.89, 24.87, 21.09, 21.06, 21.04, 21.02, 16.11, 16.08 ppm. ^31^P NMR (243 MHz, CD_3_CN) δ 147.37, 147.13 ppm. HRMS: [M + H]^+^ calc. for C_47_H_56_N_4_O_10_P, 867.3729; found: 867.3725.

*Synthesis of compound* **3R**: To a solution of compound **3S** (2.36 g, 4.85 mmol) in anhydrous THF (48.5 mL) at 0°C were added *p*-nitrobenzoic acid (4.05 g, 24.25 mmol, 5.0 equiv.), triphenylphosphine (6.36 g, 24.25 mmol, 5.0 equiv.), and DIAD (4.69 mL, 24.25 mmol, 5.0 equiv.). The reaction was allowed to stir at room temperature overnight, and the solvent was removed under reduced pressure. The crude material was purified by flash column chromatography (0–25% EtOAc in hexanes) to obtain *p*-nitrobenzyl ester as a yellowish foam (2.86 g, 4.50 mmol, R_f_ = 0.37 developed in 33% EtOAc in hexanes). This material was resuspended in 7 N ammonia in methanol (100 mL) and stirred at room temperature overnight. The solvent was removed, and the crude material was purified by flash column chromatography (0–30% EtOAc in hexanes) to yield compound **3R** as a white foam (1.47 g, 3.01 mmol, 62% over 2 steps; R_f_= 0.27, developed with 33% EtOAc in hexanes). ^1^H NMR (400 MHz, DMSO-*d*_6_) δ 11.37 (s, 1H), 7.85 (d, *J* = 8.1 Hz, 1H), 5.89 (d, *J* = 7.8 Hz, 1H), 5.71 (d, *J* = 8.0 Hz, 1H), 5.19 (d, *J* = 4.9 Hz, 1H), 4.30 (dd, *J* = 7.8, 4.5 Hz, 1H), 4.21 (d, *J* = 4.4 Hz, 1H), 3.82–3.73 (m, 1H), 3.62 (d, *J* = 4.7 Hz, 1H), 1.11 (d, *J* = 6.5 Hz, 3H), 0.89 (s, 9H), 0.81 (s, 9H), 0.10 (d, *J* = 2.9 Hz, 6H), 0.00 (s, 3H), −0.09 (s, 3H) ppm. ^13^C NMR (101 MHz, DMSO-*d*_6_) δ 162.78, 150.87, 140.66, 102.41, 90.00, 85.75, 73.90, 71.64, 66.39, 39.18, 25.69, 25.56, 20.08, 17.71, 17.57, −4.57, −4.63, −4.70, and −5.22 ppm. HRMS: [M + H]^+^ calc. for C_22_H_43_N_2_O_6_Si_2_, 487.2654; found: 487.2650.

*Synthesis of compound* **4R**: To a solution of compound **3R** (1.40 g, 2.88 mmol) in anhydrous THF (11.5 mL) and anhydrous pyridine (2.2 mL) were added DMTrCl (2.92 g, 8.63 mmol, 3 equiv.) and AgNO_3_ (0.97 g, 5.75 mmol, 2 equiv.). The mixture was stirred overnight at room temperature. The mixture was filtered over Celite, and the filter cake was washed with DCM. The filtrate was concentrated under reduced pressure, and the residue was extracted with DCM and saturated aq. NaHCO_3_. The organic layer was washed with brine, dried over anhydrous Na_2_SO_4_, filtered, and concentrated. The crude residue was purified by flash column chromatography (0–25% EtOAc in hexanes) to obtain DMTr-protected compound as a bright yellow foam (2.03 g, 2.57 mmol, 89%, R_f_ = 0.27; developed with 33% EtOAc in hexanes). ^1^H NMR (400 MHz, DMSO-*d*_6_) δ 11.38 (s, 1H), 7.46–7.38 (m, 2H), 7.30 (td, *J* = 8.1, 7.7, 5.7 Hz, 7H), 7.26–7.17 (m, 1H), 6.94–6.84 (m, 4H), 5.66 (d, *J* = 5.8 Hz, 1H), 5.31 (d, *J* = 8.0 Hz, 1H), 4.11 (dd, *J* = 4.6, 3.2 Hz, 1H), 4.06–4.02 (m, 1H), 3.86 (dd, *J* = 4.6, 3.2 Hz, 1H), 3.74 (s, 6H), 3.49–3.41 (m, 1H), 0.85 (s, 9H), 0.82 (s, 8H), 0.79 (d, *J* = 6.2 Hz, 3H), 0.06 (d, *J* = 3.2 Hz, 5H), 0.01 (s, 3H), −0.08 (s, 3H) ppm. ^13^C NMR (101 MHz, DMSO-*d*_6_) δ 158.13, 158.09, 150.42, 141.13, 135.84, 130.07, 130.03, 127.75, 127.65, 126.66, 113.09, 113.04, 101.86, 88.28, 87.24, 86.16, 73.32, 71.44, 69.53, 55.00, 25.64, 25.54, 17.62, 17.54, 17.09, −4.45, −4.64, −4.86, and −5.07 ppm. HRMS: [M + Na]^+^ calc. for C_43_H_60_N_2_O_8_Si_2_Na, 811.3786; found: 811.3787.

To a solution of fully protected compound (2.03 g, 2.57 mmol) in THF (12.9 mL) was added TBAF (5.14 g, 5.14 mmol, 2 equiv.). After stirring at room temperature overnight, the solvent was removed under reduced pressure. The crude reside was purified by flash column chromatography (0–100% EtOAc in hexanes then 2.5% MeOH in EtOAc) to obtain compound **4R** as a white foam (1.00 g, 1.78 mmol, 69%; R_f_ = 0.45, developed with 5% MeOH in EtOAc). ^1^H NMR (400 MHz, DMSO-*d*_6_) δ 11.35 (d, *J* = 2.1 Hz, 1H), 7.47–7.40 (m, 2H), 7.31 (ddd, *J* = 10.1, 7.7, 3.8 Hz, 7H), 7.25–7.18 (m, 1H), 6.95 – 6.84 (m, 4H), 5.68 (d, *J* = 5.9 Hz, 1H), 5.37 (d, *J* = 5.8 Hz, 1H), 5.18 (dd, *J* = 8.1, 2.0 Hz, 1H), 5.09 (d, *J* = 5.5 Hz, 1H), 4.18 (q, *J* = 5.3 Hz, 1H), 3.97 (q, *J* = 5.9 Hz, 1H), 3.74 (s, 6H), 3.68 (dd, *J* = 4.3, 3.1 Hz, 1H), 3.48–3.39 (m, 1H), 0.76 (d, *J* = 6.4 Hz, 3H) ppm. ^13^C NMR (101 MHz, DMSO-*d*_6_) δ 162.83, 158.10, 158.08, 150.61, 146.33, 140.77, 136.26, 136.16, 130.13, 130.09, 127.84, 127.69, 126.58, 113.10, 113.06, 101.65, 87.36, 86.92, 85.98, 72.53, 69.67, 68.96, 55.05, 55.03, and 17.08 ppm. HRMS: [M + Na]^+^ calc. for C_31_H_32_N_2_O_8_Na, 583.2051; found: 583.2060.

*Synthesis of compound* **5R**: To a solution of compound **4R** (1.00 g, 1.78 mmol) in dioxane (24 mL) and H_2_O (3 mL) was added NaIO_4_ (0.42 g, 1.96 mmol, 1.1 equiv. dissolved in 3 mL H_2_O). The bi-layer reaction mixture was vigorously stirred at room temperature for 4 h. The reaction mixture was filtered, and the filter cake was washed with additional dioxane. To the filtrate was added NaBH_4_ (0.074 g, 1.96 mmol, 1.1 equiv.). After stirring at room temperature for 2 h, the mixture was cooled to 0°C then quenched with 1:1 v/v acetic acid:pyridine. After the solvent was removed under reduced pressure, the crude residue was extracted with EtOAc and saturated aq. NaHCO_3_. The organic layer was separated, dried over anhydrous Na_2_SO_4_, filtered, and concentrated under reduced pressure. The crude material was purified by flash column chromatography (0–5% MeOH in DCM) to obtain compound **5R** as a white foam (250 mg, 0.44 mmol, 25%; R_f_ = 0.28 developed with 5% MeOH in DCM). ^1^H NMR (400 MHz, DMSO-*d*_6_) δ 11.22 (d, *J* = 2.3 Hz, 1H), 7.65 (d, *J* = 8.1 Hz, 1H), 7.37–7.30 (m, 2H), 7.29–7.14 (m, 8H), 6.90–6.77 (m, 4H), 5.65 (t, *J* = 5.8 Hz, 1H), 5.53 (dd, *J* = 8.0, 2.2 Hz, 1H), 5.10 (t, *J* = 5.9 Hz, 1H), 4.64 (t, *J* = 5.3 Hz, 1H), 3.73 (s, 7H), 3.63 – 3.43 (m, 3H), 3.30–3.14 (m, 2H), 3.04–2.95 (m, 1H), 1.23 (t, *J* = 7.3 Hz, 1H), 0.77 (d, *J* = 6.3 Hz, 3H) ppm. ^13^C NMR (101 MHz, DMSO-*d*_6_) δ 163.30, 157.97, 151.06, 145.81, 141.30, 136.45, 136.36, 129.91, 129.86, 127.90, 127.55, 126.48, 112.98, 112.96, 101.48, 85.79, 84.25, 83.07, 69.00, 61.65, 61.03, 54.98, 15.80, and 1.12 ppm. HRMS: [M + Na]^+^ calc. for C_31_H_34_N_2_O_8_Na, 585.2207; found: 585.2217.

*Synthesis of compound* **6R**: To a solution of compound **5R** (4.63 g, 7.75 mmol) in anhydrous DCM (215 mL) and pyridine (1.5 mL) cooled to -78°C, benzoyl chloride (1.0 mL, 8.53 mmol) was added dropwise. After stirring at −78°C for 1 h, the reaction mixture was brought to 0°C and quenched with ethanol (5 mL). The mixture was extracted with DCM and saturated aq. NaHCO_3_. The organic layer was washed with brine, separated, dried over anhydrous Na_2_SO_4_, filtered, and concentrated. The crude residue was purified by flash column chromatography (0–75% EtOAc in hexanes) to yield compound **6R** as a white foam (2.30 g, 3.45 mmol, 45%; R_f_ = 0.54, developed in 50% EtOAc in hexane). ^1^H NMR (400 MHz, DMSO-*d*_6_) δ 11.32 (d, *J* = 2.2 Hz, 1H), 7.88 (dd, *J* = 8.4, 1.4 Hz, 2H), 7.79 (d, *J* = 8.0 Hz, 1H), 7.70–7.63 (m, 1H), 7.53 (t, *J* = 7.8 Hz, 2H), 7.37–7.31 (m, 2H), 7.29–7.15 (m, 7H), 6.87–6.78 (m, 4H), 6.03 (dd, *J* = 6.7, 5.0 Hz, 1H), 5.53 (dd, *J* = 8.0, 2.1 Hz, 1H), 4.73 (t, *J* = 5.1 Hz, 1H), 4.55 (dd, *J* = 11.4, 5.0 Hz, 1H), 4.40 (dd, *J* = 11.4, 6.8 Hz, 1H), 3.73 (s, 6H), 3.57 (qd, *J* = 6.4, 1.8 Hz, 1H), 3.35–3.25 (m, 2H), 3.20 (dt, *J* = 11.4, 5.0 Hz, 1H), 2.97–2.91 (m, 1H), 0.85 (d, *J* = 6.3 Hz, 3H) ppm. ^13^C NMR (101 MHz, DMSO-*d*_6_) δ 164.96, 163.08, 158.02, 150.86, 145.75, 140.73, 136.37, 136.24, 133.60, 129.92, 129.85, 129.10, 129.05, 128.83, 127.88, 127.59, 126.54, 113.04, 113.00, 102.08, 85.92, 83.05, 81.25, 68.92, 63.63, 61.13, 55.00, 39.99, and 15.74 ppm. HRMS: [M + Na]^+^ calc. for C_38_H_38_N_2_O_9_Na, 689.2470; found: 689.2477.

*Synthesis of compound* **7R**: To a solution of compound **6R** (2.30 g, 3.45 mmol) in DCM (30 mL) and DIPEA (2.30 mL, 18.9 mmol) was added 2-cyanoethyl-*N*,*N*-diisopropylchlorophosphoramidite (2.41 mL, 7.57 mmol) at 0°C. The mixture was stirred at 0°C for 2 h. The reaction mixture was diluted with DCM (100 mL) then washed with saturated aq. NaHCO_3_ (100 mL). The organic layer was separated, dried over anhydrous Na_2_SO_4_, filtered, and concentrated. The crude material was purified by flash column chromatography (0–33% EtOAc in hexanes) to give compound **7R** as a white foam (2.31 g, 2.66 mmol, 77%, R_f_ = 0.41; developed with 50% EtOAc in hexanes). ^1^H NMR (600 MHz, CD_3_CN) δ 9.04 (s, 1H), 7.94 (ddd, *J* = 8.4, 4.9, 1.4 Hz, 2H), 7.67–7.59 (m, 2H), 7.52–7.40 (m, 4H), 7.36–7.08 (m, 7H), 6.87–6.79 (m, 4H), 5.99 (q, *J* = 5.0 Hz, 1H), 5.46 (d, *J* = 8.1 Hz, 1H), 4.49–4.30 (m, 2H), 3.80–3.63 (m, 9H), 3.56–3.46 (m, 2H), 3.42–3.31 (m, 1H), 2.83–2.63 (m, 1H), 2.59 (td, *J* = 5.9, 4.1 Hz, 2H), 1.15–1.09 (m, 7H), 1.09–1.04 (m, 8H) ppm. ^13^C NMR (151 MHz, CD_3_CN) δ 166.43, 166.40, 163.87, 159.73, 159.67, 151.56, 147.06, 147.00, 141.80, 137.76, 137.74, 137.27, 137.19, 134.40, 131.31, 131.28, 131.07, 131.05, 130.51, 130.50, 130.43, 130.41, 129.64, 129.06, 128.98, 128.73, 128.72, 127.75, 127.72, 119.54, 119.51, 114.13, 114.08, 114.04, 114.01, 102.99, 87.72, 87.70, 82.97, 82.95, 82.78, 82.72, 70.34, 70.30, 65.10, 65.00, 64.93, 64.89, 64.85, 64.84, 60.95, 59.51, 59.38, 59.34, 59.21, 55.91, 55.90, 43.86, 43.81, 43.78, 43.73, 24.93, 24.90, 24.87, 24.85, 24.82, 21.14, 21.02, 21.01, 20.98, 20.96, 16.53, and 16.38 ppm. ^31^P NMR (243 MHz, CD_3_CN) δ 147.33, 147.30 ppm. HRMS: [M + H]^+^ calc. for C_47_H_56_N_4_O_10_P, 867.3729; found: 867.3726.

*Synthesis of compounds* **9** *and* **10***: m*CPBA (19.9 g, 116 mmol) was added to a cooled solution of compound **8** synthesized as previously described [37] (25.0 g, 55.9 mmol) in methanol (275 mL), and the mixture was stirred overnight at room temperature. To the solution were added 10% aq. Na_2_S_2_O_3_ (100 mL) and saturated aq. NaHCO_3_ (100 mL), and the reaction mixture was stirred for 15 min. The reaction mixture was then extracted with DCM (150 mL x 3 times). The organic layers were combined, dried over anhydrous Na_2_SO_4_, filtered, and concentrated. The crude material was purified by flash column chromatography (0–2% MeOH in DCM) to give compounds **9** (1.0 g, 1.9 mmol, 3.6%, R_f_= 0.48; developed with 5% MeOH in DCM) and **10** (8.8 g, 17.5 mmol, 31%, R_f_= 0.42; developed with 5% MeOH in DCM). Compound**9**: ^1^H NMR (400 MHz, DMSO-*d*_6_) δ 11.44 (d, *J* = 2.1 Hz, 1H), 7.42 (d, *J* = 8.2 Hz, 1H), 6.01 (d, *J* = 7.4 Hz, 1H), 5.81 (dd, *J* = 8.1, 2.0 Hz, 1H), 4.74 (t, *J* = 4.9 Hz, 1H), 4.56 (dd, *J* = 7.4, 3.7 Hz, 1H), 4.00 (d, *J* = 3.6 Hz, 1H), 3.55 (t, *J* = 4.3 Hz, 2H), 3.31 (s, 1H), 3.29 (s, 3H), 0.86 (d, *J* = 40.3 Hz, 18H), 0.12 (d, *J* = 8.3 Hz, 6H), −0.01 (s, 3H), −0.09 (s, 3H) ppm. ^13^C NMR (126 MHz, DMSO-*d*_6_) δ 162.60, 150.98, 140.16, 109.53, 103.49, 86.14, 75.18, 74.91, 55.45, 48.85, 25.82, 25.77, 17.99, 17.77, −4.33, −4.39, −4.97, and −5.10 ppm. HRMS: [M + Na]^+^ calc. for C_22_H_42_N_2_O_7_Si_2_Na, 525.2423; found: 525.2438. Compound**10**: ^1^H NMR (400 MHz, DMSO-*d*_6_) δ 11.41 (d, *J* = 2.2 Hz, 1H), 7.76 (d, *J* = 8.1 Hz, 1H), 5.99 (d, *J* = 7.2 Hz, 1H), 5.73 (dd, *J* = 8.4, 2.4 Hz, 1H), 5.34 (t, *J* = 5.6 Hz, 1H), 4.32 (dd, *J* = 7.2, 5.2 Hz, 1H), 4.22 (d, *J* = 5.2 Hz, 1H), 3.54–3.41 (m, 2H), 3.30 (s, 3H), 0.90 (s, 9H), 0.80 (s, 9H), 0.07 (d, *J* = 6.2 Hz, 6H), −0.01 (s, 3H), −0.10 (s, 3H) ppm. ^13^C NMR (126 MHz, DMSO-*d*_6_) δ 162.60, 150.98, 140.16, 109.53, 103.49, 86.14, 75.18, 74.91, 55.45, 48.85, 25.82, 25.77, 17.99, 17.77, −4.33, −4.39, −4.97, and −5.10 ppm. HRMS: [M + Na]^+^ calc. for C_22_H_42_N_2_O_7_Si_2_Na, 525.2423; found: 525.2426.

*Synthesis of compound* **11**: To a solution of compound **10** (8.8 g, 17.5 mmol) in anhydrous pyridine (60 mL) was added DMTrCl (8.89 g, 26.3 mmol), and the mixture was stirred at room temperature overnight. After removing the solvent, the residue was extracted with DCM and saturated aq. NaHCO_3_. The organic layer was dried over anhydrous Na_2_SO_4_, filtered, and concentrated. The crude material was purified by flash column chromatography to give the DMTr-protected compound (14.1 g, 17.9 mmol, 99%, R_f_= 0.77; developed in 50% EtOAC in hexanes). ^1^H NMR (400 MHz, DMSO-*d*_6_) δ 11.46 (s, 1H), 7.61 (d, *J* = 8.1 Hz, 1H), 7.46–7.37 (m, 2H), 7.33–7.23 (m, 6H), 7.22–7.15 (m, 1H), 6.86 (dd, *J* = 9.0, 3.1 Hz, 4H), 6.01 (d, *J* = 4.9 Hz, 1H), 5.80 (d, *J* = 8.1 Hz, 1H), 4.43 (t, *J* = 4.7 Hz, 1H), 4.03 (d, *J* = 4.4 Hz, 1H), 3.71 (s, 6H), 3.65 (d, *J* = 10.7 Hz, 1H), 3.45 (s, 3H), 2.88 (d, *J* = 10.7 Hz, 1H), 0.71 (s, 8H), 0.63 (s, 8H), −0.03 (s, 3H), −0.04 (d, *J* = 3.6 Hz, 6H), −0.19 (s, 3H) ppm. ^13^C NMR (126 MHz, DMSO-*d*_6_) δ 158.07, 150.80, 144.86, 140.29, 135.71, 129.59, 129.57, 127.77, 127.55, 126.57, 113.20, 113.12, 108.03, 102.81, 88.51, 75.64, 74.36, 64.56, 54.97, 52.32, 25.63, 25.50, 17.51, 17.44, −4.33, −4.81, −4.86, and −5.18 ppm. HRMS: [M + Na]^+^ calc. for C_43_H_60_N_2_O_9_Si_2_Na, 827.3730; found: 827.3710.

To a solution of the fully protected compound (12.1 g, 15.0 mmol) in THF (100 mL) was added 1 M TBAF in THF (30 mL, 30 mmol). The reaction was stirred overnight under argon atmosphere, and the next day the solvent was evaporated under vacuum. The crude material was purified with column chromatography (0–2% MeOH in EtOAc) to yield compound **11** (8.31 g, 14.4 mmol, 96%, R_f_= 0.48; developed with 10% MeOH in DCM). ^1^H NMR (400 MHz, DMSO-*d*_6_) δ 11.35 (s, 1H), 7.48–7.42 (m, 2H), 7.39 (d, *J* = 8.2 Hz, 1H), 7.34–7.17 (m, 8H), 6.87 (d, *J* = 8.9 Hz, 3H), 5.98 (d, *J* = 7.4 Hz, 1H), 5.71 (d, *J* = 5.1 Hz, 2H), 5.52 (d, *J* = 6.7 Hz, 1H), 4.44 (td, *J* = 7.2, 4.2 Hz, 1H), 4.05 (t, J = 4.4 Hz, 1H), 3.72 (d, *J* = 1.3 Hz, 7H), 3.44 (d, *J* = 9.8 Hz, 1H), 3.31 (s, 3H), 2.83 (s, 4H), 2.80 (d, *J* = 3.5 Hz, 1H), 1.98 (s, 1H) ppm. ^13^C NMR (126 MHz, DMSO-*d*_6_) δ 162.86, 158.13, 151.19, 144.65, 140.25, 135.48, 134.95, 129.99, 129.86, 127.83, 127.77, 126.73, 113.09, 113.07, 109.33, 103.11, 86.93, 85.32, 73.77, 73.22, 59.81, 57.26, 55.04, 48.13, and 25.85 ppm. HRMS: [M + Na]^+^ calc. for C_31_H_32_N_2_O_9_Na, 599.2000; found: 599.2009.

*Synthesis of compound* **12**: Compound **11** (7.28 g, 12.6 mmol) was dissolved in dioxane (85 mL) and water (15 mL). NaIO_4_ (3.24 g, 15.2 mmol) was added slowly to this solution while stirring at room temperature. The reaction mixture was stirred at room temperature overnight then filtered, and the precipitate was washed with additional dioxane (100 mL). To the filtrate, NaBH_4_ (0.500 g, 19.0 mmol) was added, and the reaction mixture was stirred at room temperature for 3 h. The reaction was quenched with 20 mL of 1:1 mixture of pyridine and acetic acid. After removing the solvents, the residue was extracted with DCM and saturated aq. NaHCO_3_. The organic layer was dried over anhydrous Na_2_SO_4_, filtered, and concentrated. The crude material was purified by flash column chromatography (0–3% MeOH in DCM) to give compound **12** (5.40 g, 9.33 mol, 74%, R_f_ = 0.26; developed with 5% MeOH in DCM). ^1^H NMR (400 MHz, DMSO-*d*_6_) δ 11.12 (s, 1H), 7.47 (d, *J* = 8.0 Hz, 1H), 7.42 – 7.12 (m, 9H), 6.95 – 6.82 (m, 4H), 5.84 (t, *J* = 5.8 Hz, 1H), 5.50 (d, *J* = 8.0 Hz, 1H), 5.08 (s, 1H), 4.85 (t, *J* = 3.9 Hz, 1H), 3.73 (s, 6H), 3.44 (dd, *J* = 7.1, 3.9 Hz, 3H), 3.26 (d, *J* = 9.7 Hz, 1H), 2.90 (s, 3H), 2.87 (d, *J* = 9.6 Hz, 1H) ppm. ^13^C NMR (126 MHz, DMSO-*d*_6_) δ 163.40, 158.04, 150.41, 144.66, 141.80, 135.46, 135.09, 129.75, 129.70, 127.78, 127.66, 126.64, 113.15, 113.11, 102.82, 100.91, 75.32, 61.95, 59.63, 58.85, 54.99, 48.13, 45.55, and 10.23 ppm. HRMS: [M + Na]^+^ calc. for C_31_H_34_N_2_O_9_Na, 601.2157; found: 601.2161.

*Synthesis of compound* **13**: To a solution of compound **12** (4.70 g, 8.12 mmol) in DCM (175 mL) and pyridine (7 mL) at −78°C was added a solution of benzoyl chloride (1.04 mL, 8.9 mmol) in DCM (50 mL) over a period of 30 min. The reaction mixture was then stirred for 1 h at −78°C and then allowed warm to room temperature, at which point 5 mL of ethanol was added to quench the reaction. The mixture was washed with saturated aq. NaHCO_3_ (250 mL) and extracted with DCM (100 mL x 3 times). The organic layers were combined, dried over anhydrous Na_2_SO_4_, and concentrated. The crude material was purified by flash column chromatography (0–3% MeOH in DCM) to yield compound **13** (2.73 g, 4.0 mmol, 49%, R_f_= 0.30; developed with 5% MeOH in DCM). ^1^H NMR (400 MHz, DMSO-*d*_6_) δ 11.24 (s, 1H), 7.85–7.77 (m, 2H), 7.68 (d, *J* = 8.1 Hz, 1H), 7.66–7.62 (m, 1H), 7.49 (t, *J* = 7.8 Hz, 2H), 7.43–7.37 (m, 2H), 7.33–7.14 (m, 7H), 6.86 (d, *J* = 8.9 Hz, 3H), 6.22 (t, *J* = 6.2 Hz, 1H), 5.59 (d, *J* = 8.1 Hz, 1H), 4.99 (t, *J* = 4.2 Hz, 1H), 4.38 (d, *J* = 6.1 Hz, 2H), 3.73 (dd, J = 11.5, 4.1 Hz, 1H), 3.70 (s, 6H), 3.47 (dd, *J* = 11.5, 4.1 Hz, 1H), 3.26 (d, *J* = 9.9 Hz, 1H), 2.98 (d, *J* = 10.2 Hz, 1H), and 2.96 (s, 3H) ppm. ^13^C NMR (126 MHz, DMSO-*d*_6_) δ 164.86, 163.13, 158.07, 150.30, 144.52, 141.08, 135.23, 135.01, 133.62, 129.72, 129.70, 129.07, 128.89, 128.79, 127.81, 127.63, 126.68, 113.16, 113.13, 103.38, 101.72, 85.40, 72.77, 63.73, 59.64, 59.36, 54.96, and 48.56 ppm. HRMS: [M + Na]^+^ calc. for C_38_H_38_N_2_O_10_Na, 705.2419; found: 705.2411.

*Synthesis of compound* **14**: To a solution of compound **13** (2.58 g, 3.77 mmol) in DCM (20 mL) were added DIPEA (1.38 mL, 11.31 mmol) and 2-cyanoethyl-*N,N*-diisopropylchlorophosphoramidite (1.32 mL, 4.15 mmol) at 0°C. The reaction mixture was allowed to warm room temperature and was stirred for 3 h. The reaction mixture was diluted with DCM (100 mL) then washed with saturated aq. NaHCO_3_ (100 mL). The organic layer was separated, dried over anhydrous Na_2_SO_4_, filtered, and concentrated. The residue was dissolved in minimal DCM in 1 L of hexanes. Solids were collected by dissolving in DCM and then concentrated to yield compound **14** (3.89 g, 4.4 mmol, quant.) as a white foam. ^1^H NMR (600 MHz, CD_3_CN) δ 9.17 (s, 1H), 7.90 (d, *J* = 8.3 Hz, 1H), 7.65–7.59 (m, 1H), 7.55 (dd, *J* = 11.3, 8.1 Hz, 1H), 7.51–7.44 (m, 4H), 7.39–7.26 (m, 6H), 7.25–7.19 (m, 1H), 6.88–6.81 (m, 4H), 6.28 (ddd, *J* = 11.4, 6.9, 4.6 Hz, 1H), 5.69 (dd, *J* = 11.6, 8.1 Hz, 1H), 4.45–4.31 (m, 2H), 4.16–4.00 (m, 1H), 3.90–3.57 (m, 10H), 3.55–3.43 (m, 3H), 3.41–3.11 (m, 2H), 3.05 (d, *J* = 14.7 Hz, 3H), 2.75 (t, *J* = 5.9 Hz, 1H), 2.66–2.51 (m, 2H), 1.30–1.09 (m, 24H) ppm. ^13^C NMR (151 MHz, CD_3_CN) δ 166.39, 163.89, 163.85, 159.75, 159.74, 151.33, 151.17, 145.82, 145.78, 141.82, 141.40, 136.60, 136.35, 136.33, 136.30, 134.48, 131.21, 131.20, 131.13, 131.10, 131.04, 130.39, 130.37, 130.35, 129.67, 129.06, 128.94, 128.90, 128.86, 128.84, 127.92, 127.90, 119.50, 119.44, 118.97, 114.05, 104.35, 104.28, 104.21, 103.57, 103.01, 87.22, 87.13, 74.65, 74.28, 65.00, 62.25, 62.14, 61.66, 61.56, 61.31, 60.88, 59.87, 59.75, 59.72, 59.59, 59.16, 59.13, 55.90, 55.89, 50.44, 50.25, 47.40, 45.99, 45.95, 43.84, 43.78, 43.76, 43.70, 24.99, 24.94, 24.91, 24.88, 24.84, 23.17, 23.16, 23.10, 23.09, 21.01, 20.99, 20.97, 20.95, 20.61, and 20.56 ppm. ^31^P NMR (243 MHz, CD_3_CN) δ 147.26, 147.25 ppm. HRMS: [M + H]^+^ calc. for C_47_H_56_N_4_O_11_SP, 883.3678; found: 883.3674.

*Synthesis of compound* **16**: To a solution of compound **15** synthesized as previously described [38, 39] (500 mg, 0.893 mmol) in DCM (10 mL) was added NaIO_4_ (287 mg, 1.34 mmol) in H_2_O (10 mL). The resulting mixture was vigorously stirred for 4 h, the reaction completion was confirmed by TLC. The organic layer was separated and evaporated *in vacuo*. The resulting keto-aldehyde, which was obtained as a colorless foam, was used for next step without further purification. A round-bottom flask was charged with η6-(p-cymene-*S*,*S*)-N-toluenesulfonyl-1,2-diphenylethylenediamine ruthenium(II) chloride (15 mg, 0.024 mmol, 2.5 mol%) and the keto-aldehyde (500 mg, 0.893 mmol), and the system was flushed three times with argon. A solution of sodium formate (2.27 g, 33.3 mmol) in water (13 mL) was added, followed by EtOAc (3 mL). The resulting two-phase mixture was vigorously stirred for 24 h at room temperature. The organic phase was separated, and the aqueous phase was extracted with another 10 mL of EtOAc. The solvent was removed from the combined organic layers at reduced pressure on a rotary evaporator. The crude residue was purified by flash column chromatography on silica gel (100% EtOAc) to afford compound **16** as a white foam (401 mg, 80% over 2 steps). ^1^H NMR (400 MHz, DMSO-*d_6_*): δ 11.34 (s, 1H), 7.60 (d, *J* = 8.0 Hz, 1H), 7.37–7.08 (m, 9H), 6.85 (d, *J* = 8.4 Hz, 4H), 5.48 (dd, *J* = 15.5, 6.5 Hz, 2H), 5.08 (d, *J* = 5.3 Hz, 1H), 4.72 (s, 1H), 3.72 (s, 7H), 3.55 (s, 3H), 3.10 (s, 2H), 1.03 (d, *J* = 6.4 Hz, 3H) ppm. ^13^C NMR (126 MHz, DMSO-*d*_6_) δ 163.21, 157.99, 151.08, 144.81, 141.57, 135.61, 135.51, 129.61, 129.57, 127.75, 127.63, 126.59, 113.12, 113.09, 101.20, 86.46, 85.50, 79.54, 67.11, 63.64, 60.44, 59.75, 54.99, 39.23, 18.38, and 14.09 ppm. HRMS: [M + Na]^+^ calc. for C_31_H_34_N_2_O_8_Na, 585.2213; found: 585.2224.

*Synthesis of compound* **17S**: To a solution of compound **16** (520 mg, 0.925 mmol) in dry pyridine (10 mL) was added TBSCl (154 mg, 1.02 mmol) and DMAP (11 mg, 0.09 mmol). The reaction mixture was stirred for 3 h at room temperature, and then the resulting mixture was diluted with DCM. The reaction was quenched with saturated aq. NaHCO_3_. The organic layer was washed with brine and concentrated under vacuum. The crude residue was purified by flash column chromatography on silica gel (50% EtOAc in hexanes) to afford compound **17S** as a white foam (400 mg, 64%). ^1^H NMR (400 MHz, DMSO-*d*_6_) δ 11.34 (d, *J* = 2.0 Hz, 1H), 7.62 (d, *J* = 8.0 Hz, 1H), 7.45–7.06 (m, 9H), 7.00–6.69 (m, 4H), 5.62 – 5.31 (m, 2H), 4.98 (d, *J* = 5.9 Hz, 1H), 3.73 (ddd, *J* = 41.8, 11.1, 5.2 Hz, 8H), 3.60–3.38 (m, 2H), 3.05 (d, *J* = 5.1 Hz, 2H), 1.05 (d, *J* = 6.3 Hz, 3H), 0.74 (s, 9H), −0.07 (d, *J* = 6.9 Hz, 6H) ppm. ^13^C NMR (126 MHz, DMSO-*d6*) δ 163.21, 158.01, 151.07, 144.76, 141.61, 135.59, 135.41, 129.56, 129.54, 127.71, 127.57, 126.57, 113.09, 113.06, 101.02, 85.92, 85.61, 78.31, 66.87, 63.36, 61.41, 54.96, 25.58, 18.56, 17.70, −5.65, and −5.69 ppm. HRMS: [M + Na]^+^ calc. for C_37_H_48_N_2_O_8_SiNa, 699.3078; found: 699.3067.

*Synthesis of compound* **18S**: To a solution of compound **17S** (6.6 g, 9.76 mmol) in dry DCM (100 mL) were added Et_3_N (13.5 mL, 97.6 mmol) and BzCl (5.6 mL, 48.8 mmol). The reaction mixture was stirred for 4 h at room temperature, and then the resulting mixture was diluted with DCM. The reaction was quenched with saturated aq. NaHCO_3_. The organic layer was washed with brine and concentrated under vacuum. The crude residue was purified by flash column chromatography on silica gel (25% EtOAc in hexanes) to afford compound **18S** as a colorless foam (7.8 g, 90%). ^1^H NMR (400 MHz, DMSO-*d*_6_) δ 8.07–7.11 (m, 20H), 6.98–6.69 (m, 4H), 6.02 (d, *J* = 4.6 Hz, 1H), 5.69–5.27 (m, 2H), 3.81–3.42 (m, 9H), 3.17 (qd, *J* = 10.7, 4.4 Hz, 2H), 1.37 (d, *J* = 6.5 Hz, 3H), 0.71 (s, 9H), -0.10 (d, *J* = 12.1 Hz, 6H) ppm. ^13^C NMR (126 MHz, DMSO-*d*_6_) δ 169.19, 165.01, 161.61, 158.08, 149.35, 144.66, 141.00, 135.57, 135.52, 135.30, 133.60, 130.92, 130.23, 129.62, 129.47, 129.23, 129.18, 128.73, 127.78, 127.63, 126.71, 113.14, 113.11, 101.19, 85.91, 84.66, 78.65, 69.99, 63.23, 61.81, 54.96, 25.54, 17.67, 14.99, and −5.76 ppm. HRMS: [M + Na]^+^ calc. for C_51_H_56_N_2_O_10_SiNa, 907.3602; found: 907.3611.

*Synthesis of compound* **19S**: To a solution of compound **18S** (7.3 g, 8.25 mmol) in dry THF (83 mL) was added Et_3_N·3HF (13.4 mL, 82.5 mmol) dropwise. The reaction mixture was stirred for 8 h at room temperature then diluted with EtOAc and quenched with saturated aq. NaHCO_3_. The organic layer was washed with brine and concentrated under vacuum. The crude residue was purified by flash column chromatography on silica gel (50% EtOAc in hexanes) to afford compound **19S** as a colorless foam (6.1 g, 96%). ^1^H NMR (400 MHz, DMSO-*d*_6_) δ 8.05–7.11 (m, 20H), 7.04–6.67 (m, 4H), 6.04 (d, *J* = 3.9 Hz, 1H), 5.67–5.25 (m, 2H), 4.85 (t, *J* = 5.1 Hz, 1H), 3.72–3.70(m, 7H), 3.60–3.38 (m, 2H), 3.18 (qd, *J* = 10.7, 5.1 Hz, 2H), 1.39 (d, *J* = 6.5 Hz, 3H) ppm. ^13^C NMR (126 MHz, DMSO-*d*_6_) δ 169.19, 165.10, 161.60, 158.05, 149.26, 144.64, 141.00, 135.56, 135.50, 135.37, 133.56, 130.95, 130.19, 129.65, 129.47, 129.21, 129.17, 128.73, 127.78, 127.70, 126.72, 113.14, 113.12, 100.84, 85.83, 84.54, 79.12, 70.06, 63.63, 60.48, 54.97, and 14.94 ppm. HRMS: [M + Na]^+^ calc. for C_45_H_42_N_2_O_10_Na, 793.2737; found: 793.2724.

*Synthesis of compound* **20S**: To a solution of compound **19S** (568 mg, 0.737 mmol) in dry DCM (8 mL) were added DIPEA (385 μL, 2.21 mmol) and 2-cyanoethyl-*N*,*N*-diisopropylchlorophosphoramidite (181 μL, 0.811 mmol) dropwise. The reaction mixture was stirred for 1 h at room temperature, then diluted with DCM. The reaction was quenched with saturated aq. NaHCO_3_. The organic layer was washed with brine and concentrated under vacuum. The crude residue was purified by flash column chromatography on silica gel (30% EtOAc in hexanes) to afford compound **20S** as a white foam (623 mg, 87%). ^1^H NMR (500 MHz, CD_3_CN) δ 8.09–7.89 (m, 4H), 7.86–7.13 (m, 16H), 6.95–6.74 (m, 4H), 6.01 (dd, *J* = 8.7, 3.7 Hz, 1H), 5.45 (dtt, *J* = 21.1, 8.2, 4.1 Hz, 2H), 3.90–3.60 (m, 11H), 3.51 (ddq, *J* = 13.5, 10.3, 6.7 Hz, 2H), 3.44–3.10 (m, 2H), 2.54 (dt, *J* = 8.8, 5.9 Hz, 2H), 1.44 (t, *J* = 6.2 Hz, 3H), 1.32–0.93 (m, 12H) ppm. ^13^C NMR (101 MHz, CD_3_CN) δ 169.12, 165.27, 161.69, 158.37, 149.44, 144.58, 140.43, 135.10, 133.09, 131.14, 129.94, 129.69, 129.67, 129.39, 129.11, 128.33, 127.69, 127.54, 126.57, 118.12, 116.96, 112.76, 112.75, 100.82, 84.61, 69.86, 54.58, 42.58, 42.46, 23.67, 23.62, 23.55, 23.48, 19.68, and 19.62 ppm. ^31^P NMR (202 MHz, CD_3_CN) δ 149.67; 149.29 ppm. HRMS: [M + H]^+^ calc. for C_54_H_60_N_4_O_11_P, 971.3996; found: 971.3989.

*Synthesis of compound* **17R**: To a solution of compound **17S** (2.2 g, 3.25 mmol) in dry THF (100 mL) were added PPh_3_ (4.26 g, 16.3 mmol), BzOH (1.98 g, 16.3 mmol), and DIAD (3.15 mL, 16.3 mmol) dropwise. The reaction mixture was stirred for 3 h at room temperature, and reaction completion was confirmed by TLC. The solvent was removed under vacuum. The crude residue was purified by flash column chromatography on silica gel to afford a mixture of 2,2′-anhydro-nucleoside and DIAD byproducts. This mixture was dissolved in THF (50 mL). To the mixture was added 1 N aq. NaOH (10 mL) dropwise. The resulting mixture was stirred for 3 h. The solvent was removed under vacuum. The crude residue was purified by flash column chromatography on silica gel (50% EtOAc in hexanes) to afford compound **17R** as a white foam (1.8 g, 80%). ^1^H NMR (400 MHz, DMSO-*d*_6_) δ 11.30 (d, *J* = 2.1 Hz, 1H), 7.62 (d, *J* = 8.1 Hz, 1H), 7.43–7.04 (m, 9H), 6.96–6.65 (m, 4H), 5.60–5.40 (m, 2H), 5.07 (d, *J* = 5.4 Hz, 1H), 3.96–3.44 (m, 10H), 3.11–2.77 (m, 2H), 1.14 (d, *J* = 6.2 Hz, 3H), 0.75 (s, 9H), and −0.05 (s, 6H) ppm. ^13^C NMR (126 MHz, DMSO-*d*_6_) δ 163.22, 158.00, 157.99, 151.78, 144.75, 141.42, 135.58, 135.38, 129.51, 129.46, 127.70, 127.53, 126.56, 113.10, 113.06, 101.70, 86.25, 85.44, 77.71, 66.11, 63.07, 61.51, 54.96, 25.59, 19.72, 17.73, −5.66, and −5.68 ppm. HRMS: [M + Na]^+^ calc. for C_37_H_48_N_2_O_8_SiNa, 699.3078; found: 699.3099.

*Synthesis of compound* **18R**: To a solution of compound **17R** (3.3 g, 4.88 mmol) in dry DCM (50 mL) were added Et_3_N (6.8 mL, 48.8 mmol) and BzCl (2.8 mL, 24.4 mmol). The reaction mixture was stirred for 4 h at room temperature, and then the resulting mixture was diluted with DCM. The reaction was quenched with saturated aq. NaHCO_3_. The organic layer was washed with brine and concentrated under vacuum. The crude residue was purified by flash column chromatography on silica gel (25% EtOAc in hexanes) to afford compound **18R** as a colorless foam (4.0 g, 92%). ^1^H NMR (400 MHz, DMSO-*d*_6_) δ 8.11–7.76 (m, 3H), 7.76–7.56 (m, 4H), 7.48 (t, *J* = 7.7 Hz, 2H), 7.39–7.02 (m, 11H), 7.08–6.69 (m, 4H), 6.03 (d, *J* = 7.1 Hz, 1H), 5.83 (d, *J* = 8.2 Hz, 1H), 5.40 (p, *J* = 6.4 Hz, 1H), 3.70 (s, 9H), 3.19–2.93 (m, 2H), 1.41 (d, *J* = 6. Hz, 3H), 0.75 (s, 9H), −0.06 (d, *J* = 1.4 Hz, 6H) ppm. ^13^C NMR (101 MHz, DMSO-*d*_6_) δ 164.48, 161.58, 158.07, 158.05, 149.77, 141.63, 135.50, 135.42, 135.31, 133.72, 130.77, 129.81, 129.61, 129.55, 129.34, 129.21, 128.99, 128.87, 127.77, 127.58, 126.66, 113.16, 113.12, 102.04, 85.74, 78.75, 70.38, 62.97, 61.91, 54.96, 39.97, 25.59, 17.71, 16.19, −5.67, and −5.71 ppm. HRMS: [M + Na]^+^ calc. for C_51_H_56_N_2_O_10_SiNa, 907.3602; found: 907.3616.

*Synthesis of compound* **19R**: To a solution of compound **18R** (3.8 g, 4.30 mmol) in dry THF (43 mL) was slowly added Et_3_N·3HF (6.98 mL, 43.0 mmol). The reaction mixture was stirred for 8 h at room temperature, and then the resulting mixture was diluted with DCM. The reaction was quenched with saturated aq. NaHCO_3_. The organic layer was washed with brine and concentrated under vacuum. The crude residue was purified by flash column chromatography on silica gel (50% EtOAc in hexanes) to afford compound **19R** as a colorless foam (3.2 g, 97%). ^1^H NMR (400 MHz, DMSO-*d*_6_) δ 7.96 (dd, *J* = 8.2, 1.4 Hz, 1H), 7.90 (dt, *J* = 8.3, 1.4 Hz, 2H), 7.74–7.61 (m, 4H), 7.48 (t, *J* = 7.7 Hz, 2H), 7.40–7.16 (m, 11H), 6.86 (d, *J* = 8.4 Hz, 4H), 6.03 (dd, *J* = 7.1, 2.0 Hz, 1H), 5.84 (d, *J* = 8.1 Hz, 1H), 5.46–5.34 (m, 1H), 4.86 (td, *J* = 5.1, 1.7 Hz, 1H), 3.66–3.62 (m, 7H), 3.52 (s, 2H), 3.21–3.01 (m, 2H), and 1.48–1.37 (m, 3H) ppm. ^13^C NMR (101 MHz, DMSO-*d*_6_) δ 169.14, 164.53, 161.60, 158.03, 158.01, 149.76, 144.74, 141.66, 135.50, 135.43, 133.70, 130.82, 129.80, 129.65, 129.57, 129.35, 129.22, 129.02, 128.87, 127.78, 127.64, 126.65, 113.15, 113.13, 101.94, 85.62, 84.94, 79.38, 70.53, 63.44, 60.51, 54.96, 16.33, and 14.07 ppm. HRMS: [M + Na]^+^ calc. for C_45_H_42_N_2_O_10_Na, 793.2737; found: 793.2742.

*Synthesis of compound* **20R**: To a solution of compound **19R** (543 mg, 0.705 mmol) in dry DCM (7 mL) were added DIPEA (368 μL, 2.12 mmol) and 2-cyanoethyl-*N*,*N*-diisopropylchlorophosphoramidite (173 μL, 0.776 mmol) dropwise. The reaction mixture was stirred for 1 h at room temperature, and then the resulting mixture was diluted with DCM. The reaction was quenched with saturated aq. NaHCO_3_. The organic layer was washed with brine and concentrated under vacuum. The crude residue was purified by flash column chromatography on silica gel (30% EtOAc in hexanes) to afford compound **20R** as a colorless foam (549 mg, 80%). ^1^H NMR (500 MHz, CD_3_CN) δ 7.95 (ddd, *J* = 8.4, 2.7, 1.4 Hz, 2H), 7.81–7.56 (m, 5H), 7.52–7.35 (m, 4H), 7.35–7.16 (m, 9H), 6.90–6.77 (m, 4H), 6.04 (dd, *J* = 8.7, 6.8 Hz, 1H), 5.39 (dt, *J* = 11.2, 6.5 Hz, 1H), 3.86–3.61 (m, 11H), 3.62–3.40 (m, 2H), 3.33–3.11 (m, 2H), 2.56 (q, *J* = 5.9 Hz, 2H), 1.45 (dd, *J* = 6.4, 3.9 Hz, 3H), 1.30–0.97 (m, 12H) ppm. ^13^C NMR (101 MHz, CD_3_CN) δ 166.05, 163.01, 159.76, 151.29, 146.07, 142.03, 142.00, 136.94, 136.84, 136.31, 134.59, 132.37, 131.05, 131.01, 130.97, 130.67, 130.56, 130.44, 129.82, 129.04, 128.91, 127.92, 119.52, 118.35, 114.16, 114.14, 103.32, 87.38, 86.03, 71.83, 71.78, 64.57, 55.98, 44.01, 43.90, 43.88, 25.11, 25.09, 25.02, 24.99, 24.96, 24.89, 21.10, 21.03, 17.08, 17.04, 2.01, 1.80, and 1.67 ppm. ^31^P NMR (202 MHz, CD_3_CN) δ 149.61, 149.29 ppm. HRMS: [M + H]^+^ calc. for C_54_H_60_N_4_O_11_P, 971.3996; found: 971.3967.

*Synthesis of compound* **22**: To a solution of 1-*O*-acetyl-2,3,5-tri-*O*-benzoly-L-rhamnofuranose (compound **21**; 3.70 g, 7.14 mmol) synthesized as described previously [40] and uracil (1.61 g, 14.3 mmol) in acetonitrile (MeCN, 80 mL) was added *N*,*O*-bis(trimethylsilyl)acetamide (10.6 mL, 42.9 mmol). The mixture was refluxed for 1 h, and Me_3_SiOTf (1.55 mL, 8.57 mmol) was added dropwise at room temperature. After refluxing again for 1 h, the mixture was quenched with saturated NaHCO_3_ solution (100 mL), evaporated, and extracted with DCM. The organic layer was dried over MgSO_4_, concentrated, and purified by column chromatography on silica gel (25% EtOAc in hexanes) to afford compound **22** as a white foam (3.22 g, 79%, R_f_ = 0.15; developed with 25% EtOAc in hexanes). ^1^H NMR (500 MHz, DMSO-*d*_6_) δ 11.46 (d, *J* = 2.0 Hz, 1H), 7.95–7.30 (m, 16H), 6.26–6.19 (m, 2H), 6.10–6.07 (m, 1H), 5.73 (dd, *J* = 8.5, 2.0 Hz, 1H), 5.41–5.34 (m, 1H), 5.05 (dd, *J* = 8.5, 3.0 Hz, 1H), 1.43 (d, *J* = 6.0 Hz, 3H) ppm. ^13^C NMR (126 MHz, DMSO-*d*_6_) δ 164.59, 164.53, 164.41, 163.12, 150.61, 142.42, 133.90, 133.83, 133.42, 129.29, 129.22, 129.16, 129.10, 128.82, 128.65, 128.61, 128.59, 128.24, 102.34, 89.13, 81.75, 74.11, 71.96, 67.81, and 17.22 ppm. HRMS: [M + H]^+^ calc. for C_31_H_27_N_2_O_9_Na, 593.1536; found: 593.1545

*Synthesis of compound* **23**: Compound **22** (9.60 g, 16.8 mmol) was dissolved in 1 N NH_3_ in MeOH (168 mL). The resulting mixture was stirred for 3 days at room temperature, and solvent was removed by evaporation. The crude residue was purified by column chromatography on silica gel (10% MeOH in EtOAc) to afford compound **23** as a white powder (3.41 g, 80%, R_f_ = 0.23; developed with 10% MeOH in EtOAc). ^1^H NMR (400 MHz, DMSO-*d*_6_) δ 11.29 (brs, 1H), 7.75 (d, *J* = 11.5 Hz, 1H), 5.77 (d, *J* = 8.5 Hz, 1H), 5.64 (d, *J* = 10.0 Hz, 1H), 5.33 (d, *J* = 8.0 Hz, 1H), 5.03 (d, *J* = 1.5 Hz, 1H), 4.56 (d, *J* = 6.5 Hz, 1H), 4.39–4.31 (m, 1H), 4.06 (brs, 1H), 3.91 (dd, *J* = 3.0, 10.5 Hz, 1H), 3.86–3.76 (m, 1H), 1.05 (d, *J* = 7.5 Hz, 3H) ppm.^13^C NMR (126 MHz, DMSO-*d*_6_) δ 163.11, 150.95, 141.90, 102.07, 88.24, 85.40, 74.32, 70.52, 63.45, and 20.53 ppm. HRMS: [M + Na]^+^ calc. for C_10_H_14_N_2_O_6_Na, 281.0744; found: 281.0619.

*Synthesis of compound* **24**: To a solution of compound **23** (640 mg, 2.48 mmol) in a mixture of 1,4-dioxane (25 mL) and water (5 mL), was added NaIO_4_ (591 mg, 2.72 mmol). The reaction mixture was stirred for 1.5 h at room temperature and then diluted with 1,4-dioxane and filtered through a Celite pad. The solid residue was washed with 1,4-dioxane. Sodium borohydrate (94.0 mg, 2.48 mmol) was added to the filtrate. The resulting mixture was stirred for 15 min, and the solvent was removed under vacuum. The crude residue was purified by column chromatography on silica gel (10–20% MeOH in EtOAc) to afford compound **24** as a colorless sticky glass (548 g, 85%, R_f_ = 0.34; developed with 15% MeOH in EtOAc). ^1^H NMR (400 MHz, DMSO-*d*_6_) δ 11.16 (brs, 1H), 7.60 (d, *J* = 8.0 Hz, 1H), 5.83 (m, 1H), 5.55 (d, *J* = 8.0 Hz, 1H), 5.06 (m, 1H), 4.68 (d, *J* = 5.2 Hz, 1H), 4.49–4.41 (m, 1H), 3.76–3.67 (m, 1H), 3.65–3.46 (m, 1H), 3.38–3.21 (m, 3H), 1.05 (d, *J* = 6.4 Hz, 1H) ppm. ^13^C NMR (101 MHz, DMSO-*d*_6_) δ 163.49, 151.39, 141.61, 101.00, 84.33, 83.90, 65.85, 61.46, 60.73, and 18.25 ppm. HRMS: [M + H]^+^ calc. for C_10_H_16_N_2_O_6_Na, 283.0906; found: 283.0916.

*Synthesis of compound* **25S**: To a solution of compound **24** (2.04 g, 7.84 mmol) in dry pyridine (80 mL) were added DMTrCl (2.89 g, 8.63 mmol) and 4-dimethylaminopyridine (95.8 mg, 0.784 mmol). The reaction mixture was stirred for 12 h at room temperature and then diluted with DCM. The reaction was quenched with saturated aq. NaHCO_3_. The organic layer was washed with brine, and the solvent was removed under vacuum. The crude residue was purified by column chromatography on silica gel (EtOAc) to afford compound **25S** a white foam (1.32 g, 30%, R_f_ = 0.52; developed with EtOAc). ^1^H NMR (400 MHz, DMSO-*d*_6_) δ 11.31 (d, *J* = 1.2 Hz, 1H), 7.58 (d, *J* = 8.0 Hz, 1H), 7.35–7.10 (m, 9H), 6.88–6.81 (m, 4H), 5.89–5.80 (m, 1H), 5.46 (dd, *J* = 1.2, 8.0 Hz, 1H), 5.13–5.06 (m, 1H), 4.72 (d, *J* = 4.8 Hz, 1H), 3.78–3.52 (m, 10H), 3.04–2.78 (m, 2H), and 0.87 (d, *J* = 6.4 Hz, 3H) ppm. ^13^C NMR (101 MHz, DMSO-*d*_6_) δ 163.28, 157.98, 157.96, 151.51, 144.82, 141.23, 135.66, 135.54, 129.58, 129.49, 127.77, 127.61, 126.58, 113.14, 113.11, 101.67, 85.45, 84.42, 82.56, 66.08, 63.02, 61.01, 55.03, 55.00, and 18.09 ppm. HRMS: [M + Na]^+^ calc. for C_31_H_34_N_2_O_8_Na, 585.2213; found: 585.2205.

*Synthesis of compound* **27S**: To a solution of compound **25S** (500 mg, 0.890 mmol) in dry pyridine (85 mL) were added benzoic anhydride (211 mg, 0.979 mmol) and 4-dimethylaminopyridine (10.9 mg, 0.0890 mmol) at 0°C. The reaction mixture was stirred for 5 h at room temperature and then diluted with DCM. The reaction was quenched with saturated aq. NaHCO_3_. The organic layer was washed with brine, and the solvent was removed under vacuum. The crude residue was purified by column chromatography on silica gel (50% EtOAc in hexanes) to afford compound **27S** as a white foam (474 g, 80%, R_f_ = 0.34; developed with 50% EtOAc in hexane). ^1^H NMR (400 MHz, DMSO-*d*_6_) δ 11.43 (d, *J* = 2.0 Hz, 1H), 7.96–7.82 (m, 2H), 7.82–7.43 (m, 4H), 7.41–7.04 (m, 9H), 7.02–6.73 (m, 4H), 6.32–6.12 (m, 1H), 5.51 (d, *J* = 8.1 Hz, 1H), 4.84 (d, *J* = 4.5 Hz, 1H), 4.65 (dd, *J* = 11.6, 5.2 Hz, 1H), 4.49 (dd, *J* = 11.5, 6.8 Hz, 1H), 3.79 – 3.61 (m, 8H), 3.17–2.74 (m, 2H), 0.90 (d, *J* = 6.4 Hz, 3H) ppm. ^13^C NMR (101 MHz, DMSO-*d*_6_) δ 165.02, 163.10, 157.99, 157.97, 151.10, 144.75, 140.70, 135.56, 135.46, 133.65, 129.58, 129.49, 129.16, 129.01, 128.84, 127.77, 127.60, 126.61, 113.13, 102.13, 85.58, 82.81, 81.64, 66.08, 63.34, 62.94, 55.01, 54.98, and 17.91 ppm. HRMS: [M + Na]^+^ calc. for C_38_H_38_N_2_O_9_Na, 689.2475; found: 689.2505.

*Synthesis of compound* **28S**: To a solution of compound **27S** (2.50 g, 3.75 mmol) in dry DCM (38 mL) were added DIPEA (1.97 mL, 11.3 mmol) and 2-cyanoethyl-*N*,*N*-diisopropylchlorophosphoramidite (921 μL, 14.1 mmol) dropwise. The reaction mixture was stirred for 1 h at room temperature and then diluted with DCM. The reaction was quenched with saturated aq. NaHCO_3_. The organic layer was washed with brine, and the solvent was removed under vacuum. The crude residue was purified by column chromatography on silica gel (40% EtOAc in hexanes) to afford compound **28S** as a white foam (2.79 g, 86%, R_f_ = 0.23; developed with 40% EtOAc in hexane). ^1^H NMR (500 MHz, CD_3_CN) δ 9.20 (brs, 1H); 7.99–7.93 (m, 2H), 7.64–7.18 (m, 13H), 6.85–6.80 (m, 4H), 6.34–6.28 (m, 1H), 5.49 (d, *J* = 8.0 Hz, 1H), 4.59–4.40 (m, 2H), 4.19–4.05 (m, 1H), 3.79–3.48 (m, 12H), 3.19–3.09 (m, 2H), 2.62–2.57 (m, 2H), 1.16–1.01 (m, 15H) ppm. ^13^C NMR (126 MHz, CD_3_CN) δ 166.44, 163.94, 159.62, 159.59, 151.90, 151.82, 145.97, 145.95, 141.46, 141.41, 136.80, 136.77, 136.73, 134.38, 130.91, 130.89, 130.84, 130.80, 130.46, 130.44, 130.43, 129.60, 128.88, 128.82, 128.79, 127.77, 119.52, 114.03, 114.01, 103.05, 103.00, 87.48, 87.45, 83.30, 83.26, 83.08, 82.99, 82.89, 82.86, 71.64, 71.51, 71.35, 71.21, 64.76, 64.72, 64.09, 64.06, 59.52, 59.37, 59.14, 58.99, 55.87, 55.86, 43.88, 43.87, 43.79, 43.77, 25.05, 24.99, 24.93, 24.87, 24.81, 24.79, 24.73, 21.02, 20.98, 20.96, 20.92, 17.40, 17.39, 17.22, and 17.19 ppm. ^31^P NMR (202 MHz, CD_3_CN) δ 148.94; 148.75 ppm. HRMS: [M + H]^+^ calc. for C_47_H_56_N_4_O_10_P, 867.3734; found: 867.3742.

*Synthesis of compound* **25R**: To the solution of compound **25S** (500 g, 0.890 mmol) in dry THF (9 mL) were added PPh_3_ (622 mg, 2.67 mmol), benzoic acid (543 mg, 4.45 mmol), and DIAD (526 μL, 2.67 mmol) dropwise. The reaction mixture was stirred for 5 h at room temperature, and reaction completion was confirmed by TLC. The solvent was removed under vacuum. The crude residue was purified by column chromatography on silica gel (5–10% MeOH in EtOAc) to afford the 2,2′-anhydro nucleoside **26** (R_f_ = 0.11; developed with EtOAc). ^1^H NMR (400 MHz, DMSO-*d_6_*) δ 7.93 (d, *J* = 7.6 Hz, 1H), 7.88–7.84 (m, 2H), 7.70–7.64 (m, 1H), 7.53–7.48 (m, 2H) 7.36–7.16 (m, 9H), 6.85–6.76 (m, 4H), 6.13 (dd, *J* = 5.6, 1.6 Hz, 1H), 5.76 (dt, *J* = 7.6, 12.4 Hz, 1H), 4.67 (dd, *J* = 10.0, 5.6 Hz, 1H), 4.51–4.41 (m, 2H), 3.70 (s, 3H), 3.69 (s, 3H), 3.26 (dd, *J* = 10.0, 3.6 Hz, 1H), 2.96 (dd, *J* = 10.4, 5.2 Hz, 1H), 1.15 (d, *J* = 7.2 Hz, 3H) ppm. ^13^C NMR (101 MHz, DMSO-*d_6_*) δ 171.00, 164.91, 160.23, 158.12, 158.05, 144.55, 136.77, 135.10, 135.05, 133.52, 129.62, 129.48, 129.16, 128.78, 127.86, 127.51, 126.75, 113.18, 108.72, 87.62, 85.63, 80.12, 73.40, 70.49, 62.56, 55.02, 54.98, 54.96, and 15.37 ppm. HRMS: [M + H]^+^ calc. for C_38_H_37_N_2_O_8_, 649.2550; found: 649.2546.

Compound **26** was dissolved in THF (10 mL). To the solution was added 1 N aq. NaOH (3 mL) dropwise. The resulting mixture was stirred for 12 h. The solvent was removed under vacuum. The crude residue was purified by column chromatography on silica gel (0–5% MeOH in EtOAc) to afford compound **25R** as a white foam (452 g, 90% over 2 steps, R_f_ = 0.32; developed with EtOAc). ^1^H NMR (500 MHz, DMSO-*d*_6_) δ 11.39 (brs, 1H), 7.63 (d, *J* = 8.0 Hz, 1H), 7.39–7.04 (m, 9H), 6.97–6.67 (m, 4H), 5.82 (t, *J* = 5.9 Hz, 1H), 5.49 (d, *J* = 8.0 Hz, 1H), 5.23–5.16 (m, 1H), 4.77 (d, *J* = 4.8 Hz, 1H), 3.84–3.46 (m, 10H), 3.06–2.84 (m, 2H), 0.86 (d, *J* = 6.4 Hz, 3H) ppm.^13^C NMR (101 MHz, DMSO-*d*_6_) δ 163.27, 157.97, 157.95, 151.44, 144.81, 141.22, 135.58, 135.56, 129.61, 129.52, 127.75, 127.63, 126.57, 113.10, 101.73, 85.31, 84.80, 83.02, 65.90, 62.88, 61.16, 55.00, 54.98, and 18.55 ppm. HRMS: [M + Na]^+^ calc. for C_31_H_34_N_2_O_8_Na, 585.2213; found: 585.2205.

*Synthesis of compound* **27R**: To a solution of compound **26** (2.00 g, 3.56 mmol) in dry pyridine (36 mL) were added DMAP (43.5 mg, 0.356 mmol) and Bz_2_O (845 mg, 3.74 mmol). The reaction mixture was stirred for 5 h at room temperature, and then the resulting mixture was diluted with DCM. The reaction was quenched with saturated aq. NaHCO_3_. The organic layer was washed with brine, and the solvent was removed under vacuum. The crude residue was purified by column chromatography on silica gel (50% EtOAc in hexanes) to afford compound **27R** as a colorless foam (1.99 g, 84%, R_f_ = 0.34; developed with 50% EtOAc in hexanes). ^1^H NMR (400 MHz, DMSO-*d*_6_) δ 11.45 (d, *J* = 2.0 Hz, 1H), 7.99–7.41 (m, 6H), 7.41–7.03 (m, 9H), 6.95–6.58 (m, 4H), 6.20 (t, *J* = 6.0 Hz, 1H), 5.54 (dd, *J* = 7.6, 1.6 Hz, 1H), 4.86 (d, *J* = 5.2 Hz, 1H), 4.70 (dd, *J* = 12.0, 5.6 Hz, 1H), 4.51 (dd, *J* = 12.0, 5.6 Hz, 1H), 3.86–3.52 (m, 8H), 3.10–2.91 (m, 2H), 0.84 (d, *J* = 6.4 Hz, 3H) ppm. ^13^C NMR (101 MHz, DMSO-*d*_6_) δ 165.03, 163.10, 157.98, 157.96, 151.11, 144.75, 140.76, 135.52, 135.49, 133.69, 129.61, 129.52, 129.12, 129.00, 128.87, 127.75, 127.62, 126.59, 113.10, 102.20, 85.40, 83.17, 81.90, 65.75, 63.40, 62.73, 55.00, 54.97, and 18.49 ppm. HRMS: [M + Na]^+^ calc. for C_38_H_38_N_2_O_9_Na, 689.2475; found: 689.2490.

*Synthesis of compound* **28R**: To a solution of compound **27R** (2.00 g, 3.00 mmol) in dry DCM (30 mL) were added DIPEA (1.57 mL, 9.00 mmol) and 2-cyanoethyl-*N*,*N*-diisopropylchlorophosphoramidite (737 μL, 3.30 mmol) dropwise. The mixture was stirred for 2 h at room temperature and then diluted with DCM. The reaction was quenched with saturated aq. NaHCO_3_. The organic layer was washed with brine, and the solvent was removed under vacuum. The crude residue was purified by column chromatography on silica gel (50% EtOAc in hexanes) to afford compound **28R** a white foam (2.11 g, 81%, R_f_ = 0.65; developed with 50% EtOAc in hexane). ^1^H NMR (500 MHz, CD_3_CN) δ 8.00–7.94 (m, 2H), 7.64–7.16 (m, 13H), 6.86–6.81 (m, 4H), 6.28–6.22 (m, 1H), 5.56–5.48 (m, 1H), 4.69–4.44 (m, 3H), 4.23–4.03 (m, 1H), 3.86–3.42 (m, 12H), 3.31–3.04 (m, 2H), 2.61–2.51 (m, 2H), and 1.28–0.98 (m, 15H) ppm. ^13^C NMR (126 MHz, CD_3_CN) δ 166.51, 164.07, 164.04, 159.66, 159.64, 151.98, 145.99, 141.49, 141.43, 136.89, 136.84, 136.83, 136.81, 134.54, 134.52, 131.03, 130.97, 130.47, 129.73, 129.02, 129.00, 128.85, 127.84, 127.82, 119.56, 114.11, 114.07, 103.34, 103.30, 87.26, 87.24, 83.19, 83.02, 82.99, 82.96, 82.87, 82.83, 70.43, 70.31, 64.84, 64.82, 64.01, 63.91, 59.32, 59.29, 59.17, 59.14, 58.19, 55.95, 44.06, 44.00, 43.96, 43.90, 25.09, 25.03, 24.99, 24.95, 24.94, 24.89, 24.82, 24.76, 22.03, 21.07, 21.02, 21.00, 20.95, 17.87, and 17.84 ppm. ^31^P NMR (202 MHz, CD_3_CN) δ 149.10; 148.41 ppm. HRMS: [M + H]^+^ calc. for C_47_H_56_N_4_O_10_P, 867.3734; found: 867.3760.

*Synthesis of compound* **29S**: To a solution of compound **17S** (100 mg, 0.148 mmol), DMAP (1.8 mg, 0.02 mmol), and Et_3_N (103 μL, 0.740 mmol) in dry MeCN (2 mL) was added (*R*–)-MTPACl (33.2 μL, 0.178 mmol) dropwise. The reaction mixture was stirred for 5 h at room temperature, and then the resulting mixture was diluted with EtOAc. The reaction was quenched with saturated aq. NaHCO_3_. The organic layer was washed with brine and concentrated under vacuum. The crude residue was purified by flash column chromatography on silica gel (50% EtOAc in hexanes) to afford compound **29S** as a colorless foam (70 mg, 54%). ^1^H NMR (400 MHz, DMSO-*d*_6_) δ 11.48 (s, 1H); 7.65 (d, *J* = 8.1 Hz, 2H), 7.53–7.02 (m, 13H), 6.94–6.65 (m, 4H), 5.88 (d, *J* = 6.0 Hz, 1H), 5.56–5.27 (m, 2H), 3.87–3.44 (m, 9H), 3.31 (s, 3H), 3.04 (t, *J* = 4.0 Hz, 2H), 1.20 (d, *J* = 6.4 Hz, 3H), 0.68 (s, 9H), −0.15 (d, *J* = 7.4 Hz, 6H) ppm. ^13^C NMR (126 MHz, DMSO-*d*_6_) δ 158.04, 150.98, 144.67, 135.45, 135.25, 129.94, 129.58, 128.53, 127.71, 127.54, 127.16, 126.61, 113.09, 113.06, 102.30, 77.77, 72.94, 63.27, 61.25, 55.19, 54.97, 39.06, 25.50, 17.62, 14.76, −5.83, and −5.86 ppm. HRMS: [M + Na]^+^ calc. for C_47_H_55_F_3_N_2_O_10_SiNa, 915.3476; found: 915.3484.

*Synthesis of compound* **30S**: To a solution of compound **17S** (100 mg, 0.148 mmol), DMAP (1.8 mg, 0.02 mmol), and Et_3_N (103 μL, 0.740 mmol) in dry MeCN (2 mL) was slowly added (*S*-+)-MTPACl (33.2 μL, 0.178 mmol). The reaction mixture was stirred for 5 h at room temperature, and then the resulting mixture was diluted with EtOAc. The reaction was quenched with saturated aq. NaHCO_3_. The organic layer was washed with brine and concentrated under vacuum. The crude residue was purified by flash column chromatography on silica gel (50% EtOAc in hexanes) to afford compound **30S** as a colorless foam (68 mg, 52%). ^1^H NMR (400 MHz, DMSO-*d*_6_) δ 11.44 (s, 1H), 7.60–7.08 (m, 15H), 6.84 (dd, *J* = 8.9, 3.5 Hz, 4H), 5.86 (d, *J* = 5.9 Hz, 1H), 5.48–5.19 (m, 2H), 3.72 (s, 6H), 3.51–3.34 (m, 5H), 2.93 (t, *J* = 4.4 Hz, 3H), 1.31 (d, *J* = 6.4 Hz, 3H), 0.69 (s, 9H), −0.15 (d, *J* = 9.4 Hz, 6H) ppm. ^13^C NMR (126 MHz, DMSO-*d*_6_) δ 165.03, 162.83, 158.02, 150.90, 144.65, 139.94, 135.42, 135.24, 131.20, 129.81, 129.56, 128.41, 127.68, 127.52, 126.85, 126.59, 113.06, 113.03, 102.06, 85.63, 82.96, 77.67, 72.79, 63.22, 61.07, 55.35, 54.95, 39.23, 25.48, 17.59, 15.09, −5.78,and −5.84 ppm. HRMS: [M + Na]^+^ calc. for C_47_H_55_F_3_N_2_O_10_SiNa, 915.3476; found: 915.3485.

*Synthesis of compound* **31R**: To a solution of compound **17R** (100 mg, 0.148 mmol), DMAP (1.8 mg, 0.02 mmol), and Et_3_N (103 μL, 0.740 mmol) in dry MeCN (2 mL) was slowly added (*R*)-(–)-MTPACl (33.2 μL, 0.178 mmol). The reaction mixture was stirred for 5 h at room temperature, and then the resulting mixture was diluted with EtOAc. The reaction was quenched with saturated aq. NaHCO_3_. The organic layer was washed with brine and concentrated under vacuum. The crude residue was purified by flash column chromatography on silica gel (50% EtOAc in hexanes) to afford compound **31R** as a colorless foam (102 mg, 78%). ^1^H NMR (400 MHz, DMSO-*d*_6_) δ 11.28 (d, *J* = 2.0 Hz, 1H), 7.56–7.02 (m, 15H), 6.98–6.71 (m, 4H), 5.89 (d, *J* = 7.3 Hz, 1H), 5.54–5.29 (m, 2H), 3.72 (d, *J* = 1.3 Hz, 6H), 3.67–3.44 (m, 3H), 3.40 (s, 3H); 3.11–2.85 (m, 2H), 1.39 (d, *J* = 6.2 Hz, 3H), 0.75 (s, 9H), and −0.06 (d, *J* = 1.9 Hz, 6H) ppm. ^13^C NMR (126 MHz, DMSO-*d*_6_) δ 164.54, 162.83, 158.02, 158.00, 151.10, 144.68, 135.50, 135.31, 131.00, 129.90, 129.49, 129.43, 128.57, 127.71, 127.49, 126.71, 126.58, 113.12, 113.07, 102.39, 85.54, 78.70, 71.71, 62.82, 61.77, 55.17, 54.97, 39.25, 25.57, 17.70, 16.08, −5.70, and −5.72 ppm. HRMS: [M + Na]^+^ calc. for C_47_H_55_F_3_N_2_O_10_SiNa, 915.3476; found: 915.3466.

*Synthesis of compound* **32R**: To a solution of compound **17R** (100 mg, 0.148 mmol), DMAP (1.8 mg, 0.02 mmol), and Et_3_N (103 μL, 0.740 mmol) in dry MeCN (2 mL) was added (*S*)-(+)-MTPACl (33.2 μL, 0.178 mmol) dropwise. The reaction mixture was stirred for 5 h at room temperature, and then the resulting mixture was diluted with EtOAc. The reaction was quenched with saturated aq. NaHCO_3_. The organic layer was washed with brine and concentrated under vacuum. The crude residue was purified by flash column chromatography on silica gel (50% EtOAc in hexanes) to afford compound **32R** as a colorless foam (101 mg, 77%). ^1^H NMR (400 MHz, DMSO-*d*_6_) δ 11.47 (d, *J* = 2.0 Hz, 1H), 7.67 (d, *J* = 8.1 Hz, 1H), 7.56–7.04 (m, 14H), 6.94–6.71 (m, 4H), 5.98 (d, *J* = 7.1 Hz, 1H), 5.62–5.23 (m, 2H), 3.71 (s, 6H); 3.68–3.44 (m, 2H), 3.36 (s, 3H), 3.01 (t, *J* = 5.4 Hz, 2H), 1.30 (d, *J* = 6.2 Hz, 3H), 0.74 (s, 9H), −0.06 (d, *J* = 2.7 Hz, 6H) ppm. ^13^C NMR (126 MHz, DMSO-*d*_6_) δ 164.68, 162.88, 158.01, 151.25, 144.68, 140.54, 135.48, 135.29, 131.12, 129.96, 129.51, 129.45, 128.61, 127.72, 127.49, 126.86, 126.60, 113.11, 113.07, 102.47, 78.80, 72.30, 62.90, 61.80, 55.01, 54.96, 25.56, 17.68, 15.69, and −5.71 ppm. HRMS: [M + Na]^+^ calc. for C_47_H_55_F_3_N_2_O_10_SiNa, 915.3476; found: 915.3460.

### Oligonucleotide synthesis

Oligonucleotides used for the exonuclease assay were synthesized on an ABI-394 synthesizer and those used for *in vitro* efficacy assays were synthesized on a MerMade 192 synthesizer on 1-μmol scale using universal or custom supports. A solution of 0.25 M 5-(*S*-ethylthio)-1*H*-tetrazole in MeCN was used as the activator. The solutions of commercially available phosphoramidites and synthesized modified UNA phosphoramidites were used at 0.15 M in anhydrous MeCN. The oxidizing reagent was 0.02 M I_2_ in THF/pyridine/H_2_O. The detritylation reagent was 3% dichloroacetic acid in DCM. After completion of the automated synthesis on the ABI-394, the oligonucleotide was cleaved from support and deprotected using aq. MeNH_2_ (40 wt.%) at room temperature for 90 min. After filtration through a 0.45-μm nylon filter, oligonucleotides were either purified or, for oligonucleotides containing ribose sugars, the 2′ hydroxyl was deprotected by treatment with Et_3_N·3HF at 60°C for 30 min. Oligonucleotides were purified using ion exchange HPLC (IEX-HPLC) using an appropriate gradient of mobile phase (buffer A: 0.15 M NaCl, 10% MeCN; buffer B 1.0 M NaBr, 10% MeCN) and desalted using size-exclusion chromatography with water as an eluent. Oligonucleotides were then quantified by measuring the absorbance at 260 nm. Oligonucleotide extinction coefficients were calculated using the following extinction coefficients for each residue: A, 13.86; T/U, 7.92; C, 6.57; and G, 10.53 M^−1^cm^−1^. The purity and identity of modified oligonucleotides were verified by analytical anion exchange chromatography and electrospray ionization mass spectroscopy (ESI-MS), respectively.

After the trityl-off synthesis using the MerMade 192, columns were incubated with 150 mL of 40 wt.% aq. methylamine for 30 min at room temperature, and solutions were drained via vacuum into a 96-well plate. After repeating the incubation and draining with a fresh portion of aq. methylamine, the plate containing the crude oligonucleotides was sealed and shaken at room temperature for 60 min to completely remove all protecting groups. In the case of RNA, the 2′ hydroxyl was deprotected by treating with Et_3_N·3HF at 60°C for 60 min. Precipitation of the crude oligonucleotides was accomplished via the addition of 1.2 mL of MeCN/ethanol (9:1, v/v) to each well, followed by centrifugation at 3000 rpm for 45 min at 4°C. The supernatant was removed from each well, and the pellets were resuspended in 950 mL of 20 mM aq. NaOAc. Oligonucleotides were desalted over a GE Hi-Trap desalting column (Sephadex G25 Superfine) using water as an eluant. The identities and purities of all oligonucleotides were confirmed using ESI-MS and IEX-HPLC, respectively.

### Determination of thermal denaturation temperatures

Thermal denaturation temperatures were measured with equimolar concentrations of both strands (2.5 μM) in phosphate-buffered saline (PBS) by monitoring absorbance at 260 nm with increasing temperature (1°C/min). Values were reported as the maximum of the first derivative and are the average of at least two experiments.

### Nuclease resistance assays

Oligonucleotides were prepared at final concentrations of 0.1 mg/ml in 50 mM Tris (pH 7.2), 10 mM MgCl_2_ for assays in the presence of 3′-specific snake venom phosphodiesterase (SVPD) or in 50 mM sodium acetate (pH 6.5), 10 mM MgCl_2_ for assays in the presence of 5′-specific exonuclease phosphodiesterase II (PDE-II). The exonuclease (150 mU/mL SVPD or 500 mU/mL PDE-II) was added to oligonucleotide solution immediately prior to the first injection onto the HPLC column, and enzymatic degradation kinetics were monitored for 24 h at 25°C. Samples were analyzed on a Dionex DNAPac PA200 analytical column at 30°C. The gradient was from 37% to 52% 1 M NaBr, 10% MeCN, 20 mM sodium phosphate buffer at pH 11 over 10 min with a flow rate of 1 mL/min. The full-length oligonucleotide amount was determined as the area under the curve of the peak detected at 260 nm. Percent full-length oligonucleotide was calculated by dividing the area under the curve at a given time point by that at the first time point and multiplying by 100. Activity of enzyme was verified by including a 20-mer oligodeoxythymidylate with a terminal PS linkage in each experiment. An aliquot of enzyme was thawed just prior to the experiment. The half-life was determined by fitting to first-order kinetics. Each degradation experiment was performed in duplicate.

### RT-qPCR quantification of mRNA

Primary mouse hepatocytes were transfected with siRNAs using the RNAiMAX reagent (Thermo Fisher) according to the manufacturer's recommendations. Briefly, cells were thawed just prior to transfection and plated onto a 384-well plate with a seeding density of approximately 5000 cells per well in Williams Medium E supplemented with 10% fetal bovine serum. A pre-incubated lipid/siRNA complex (0.1 μL RNAiMAX and siRNA in 5 μL Opti-MEM (Thermo Fisher), incubated for 15 min) was added to each well of a 384-well collagen-coated plate (BioCoat; Corning). Cells were incubated for 20 h at 37°C in an atmosphere of 5% CO_2_. After incubation, the media was removed, and the cells were washed and lysed. RNA was extracted using the Dynabeads mRNA isolation kit (Invitrogen) according to the manufacturer′s protocol, and then reverse-transcribed using the ABI High-Capacity cDNA Reverse Transcription Kit. Quantification was performed by real-time quantitative PCR (qPCR), where the cDNA (2 μL) was added to a master mix containing 0.5 μL mouse *GAPDH* TaqMan Probe, 5 μL of the target TaqMan probe, and 0.5 μL LightCycler 480 Probe Master Mix. RT-qPCR was performed on an ABI 7900HT Real-Time PCR System using the ΔΔCt method for quantification. Results are reported from at least four biological replicates. Each well was normalized to the *GAPDH* control, and the remaining mRNA levels were calculated relative to a non-targeting siRNA that targets Luciferase. IC_50_ values were calculated from fitted curves using GraphPad Prism.

### Analysis of off-target effects

The on-target and off-target reporters were previously described [24]. The reporter plasmids were generated by Blue Heron Biotech by cloning into the psiCHECK2 vector between XhoI and NotI restriction sites in the 3′ untranslated region (3′-UTR) of *Renilla* luciferase. The on-target reporter plasmid contained a single site perfectly complementary to the antisense strand in the 3′-UTR of *Renilla* luciferase (5′-TGTTCTTGCTCTATAAACCGTGT-3′). The off-target reporter plasmid contained four tandem seed-complementary sites (5′-AAACCGTGA-3′) separated by a 19-nucleotide spacer (5′-TAATATTACATAAATAAAA-3′) in the 3′-UTR of *Renilla* luciferase. Both the on-target and off-target regions were flanked at the 5′ ends by 5′- ATAAACAAGGTTTGACATCAATCTAGCTATATCTTTAAGAATGATAAACT-3′ and at the 3′ ends by 5′-GACATTGGTGAGGAAAAATCCTTTGGCCGTTTCCAAGATCTGACAGTGCA-3′. Both plasmids co-expressed firefly luciferase as a transfection control.

Cos7 cells (ATCC) were grown to near confluence at 37°C in an atmosphere of 5% CO_2_ in DMEM (ATCC) supplemented with 10% fetal bovine serum. Cells were released from the plate by trypsinization before transfection and resuspended in 35 μL of fresh complete media. A solution of 5 μL of siRNA (or PBS as a control), 5 μL of 1 mg/mL appropriate psiCHECK2 plasmid, 5 μL of Opti-MEM (Thermo Fisher), and 0.1 μL of Lipofectamine RNAiMAX (Thermo Fisher) was incubated at room temperature for 15 min and was then added to the cells. The transfected cells were then incubated at 37°C in an atmosphere of 5% CO_2_. At 48 h post-transfection, firefly (transfection control) and *Renilla* (fused to target sequence) luciferase activities were measured. First, the media was removed from the cells, and firefly luciferase activity was measured by adding 20 μL of Dual-Glo Luciferase Reagent (Promega) to each well. The mixture was incubated at room temperature for 30 min, and luminescence at 500 nm was measured using a Spectramax plate reader (Molecular Devices) to detect the firefly luciferase signal. *Renilla* luciferase activity was measured by adding 20 μL of Dual-Glo Stop & Glo Reagent (Promega) to each well. The plates were incubated for 10–15 min before luminescence was measured again to determine the *Renilla* luciferase signal. siRNA activity was determined by normalizing the *Renilla* signal to the firefly (control) signal within each well. The magnitude of siRNA activity was then assessed relative to cells transfected with the same vector but not treated with siRNA or treated with a non-targeting siRNA (antisense strand sequence: (5′-AAACCGTGA-3′)). All transfections were performed in triplicate.

### Treatment of mice with siRNA and quantification of TTR in serum

All studies were conducted by certified laboratory personnel using protocols consistent with local, state, and federal regulations, as applicable, and experimental protocols were approved by the Institutional Animal Care and Use Committee at Alnylam Pharmaceuticals. All animals were acclimated in-house for 48 h prior to study start. Female C57BL/6 mice, approximately 6–8 weeks of age, were obtained from Charles River Laboratories and randomly assigned to each group. All dosing solutions were stored at 4°C until 1 h before the time of injection, when they were removed from storage and allowed to reach room temperature. Animals received a single subscapular subcutaneous injection of siRNA, prepared as an injection volume of 10 μL/g in PBS, or PBS as a control. At the indicated time pre- or post-dosing, blood was collected via retro-orbital bleed. Serum samples were kept at room temperature for 1 h, then spun in a microcentrifuge at 21 000 × g at room temperature for 10 min, and subsequently stored at −80°C until analysis. Serum was diluted 1:4000, and TTR was quantified by ELISA using a mouse prealbumin kit (ALPCO, 41-PALMS-E01).

## Results

### Synthesis of RNA oligonucleotides containing modified UNAs

**Scheme 1.**
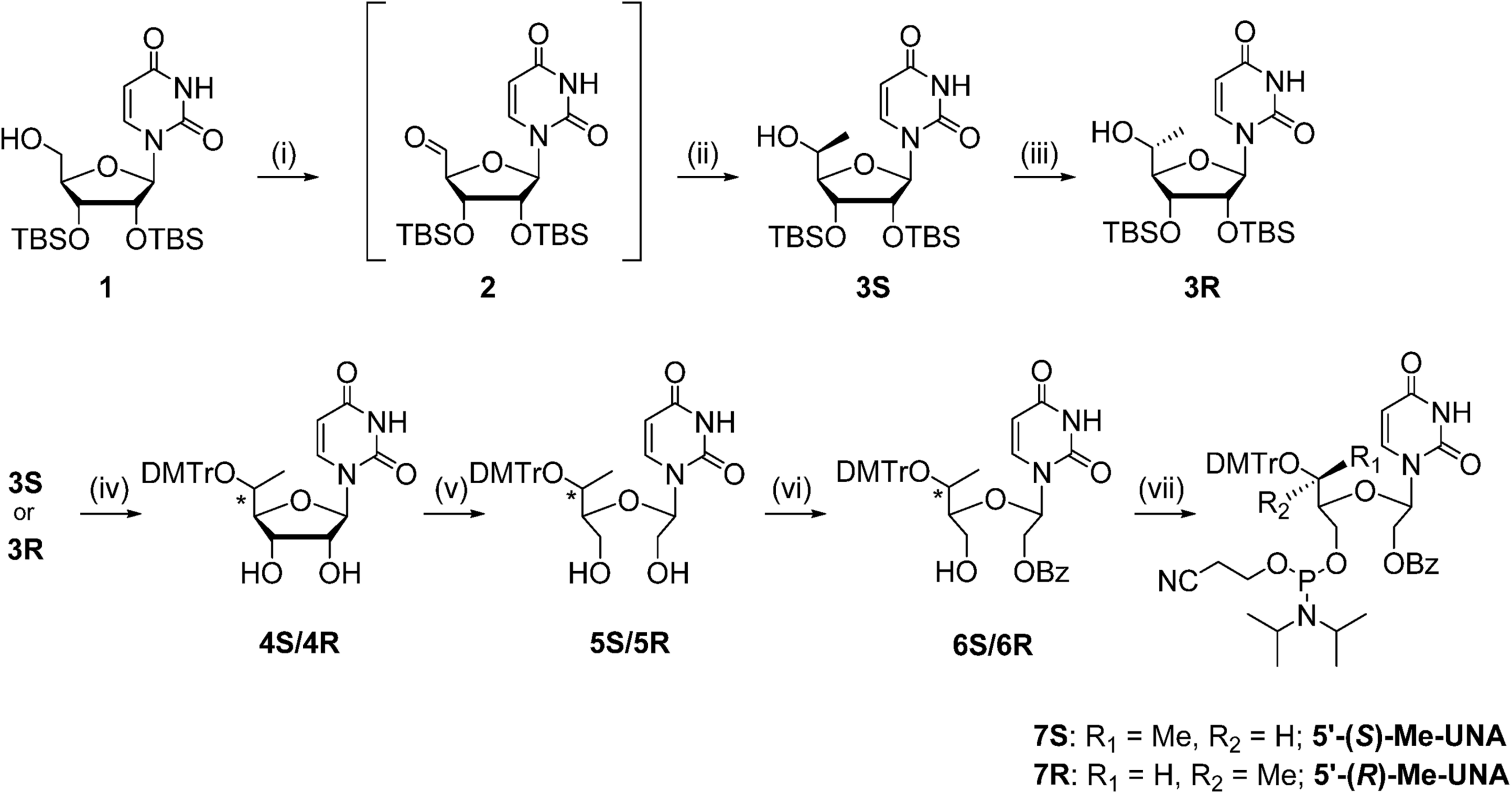
Synthesis of 5′-methyl-UNA uridine building blocks. Reagents and conditions: (i) DMP/DCM, 0°C to room temperature, 2.5 h; (ii) AlMe_3_/toluene/THF, room temperature, overnight, 25% over two steps; (iii) (a) *p*-NO_2_BzOH/PPh_3_/DIAD/THF, room temperature, overnight; (b) 7 M NH_3_/MeOH, overnight, 62% over two steps; (iv) (a) DMTrCl/Ag_2_O/pyridine/THF, room temperature, 24 h; (b) TBAF/THF, room temperature, overnight, 85% (**4S**), 61% (**4R**), over 2 steps (v) (a) NaIO_4_/1,4-dioxane/H_2_O, room temperature, 4 h; (b) NaBH_4_, room temperature, 2 h, 85% (**5S**), 25% (**5R**) over two steps; (vi) BzCl/pyridine/DCM, −78°C, 1 h, 22% (**6S**), 25% (**6R**); (vii) 2-cyanoethyl-*N*,*N*-diisopropylchlorophosphoramidite/DIPEA/DCM, room temperature, 2 h, 85% (**7S**), 73% (**7R**).

**Scheme 2.**
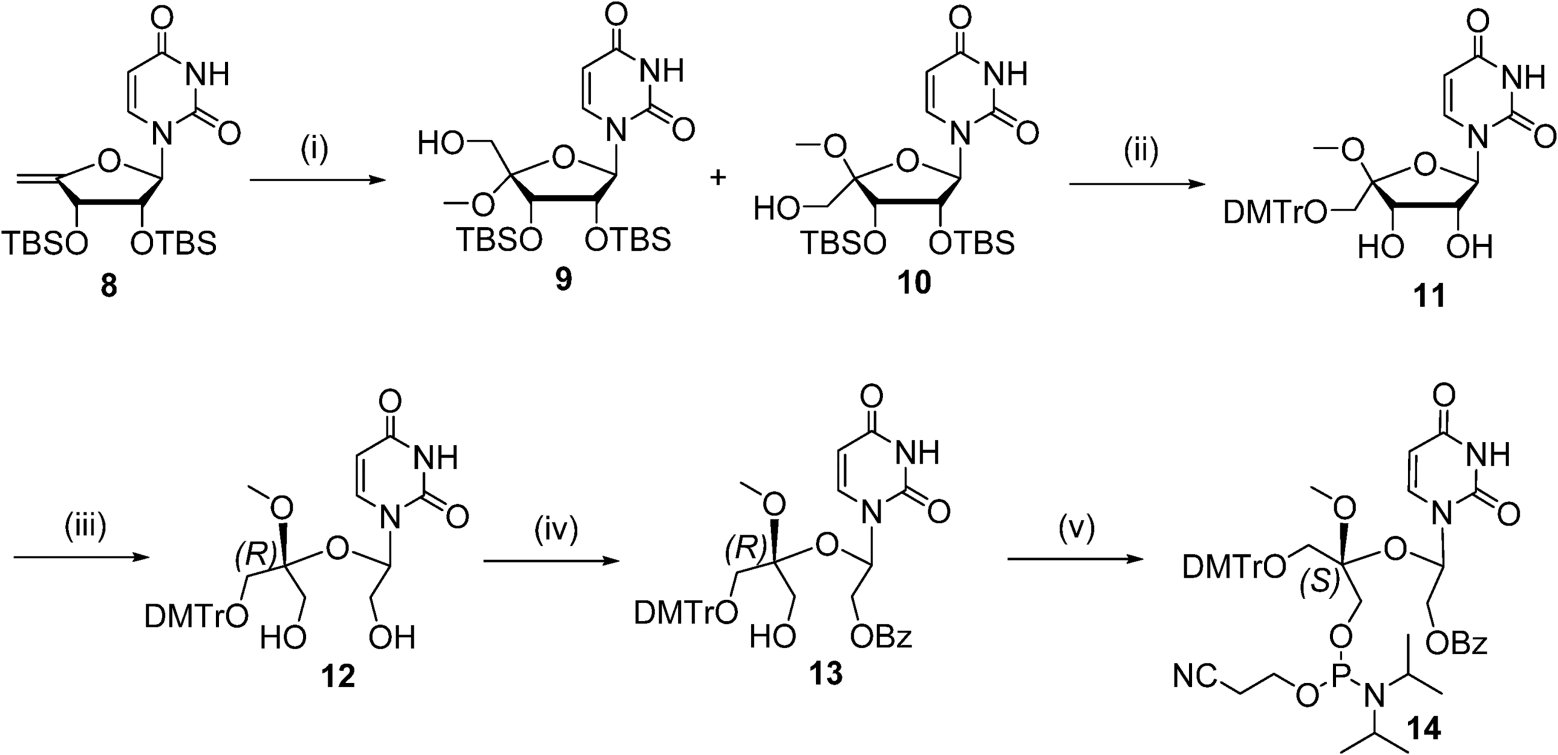
Synthesis of 4′-*C*-(β)-methoxy-UNA uridine building block **14**. Reagents and conditions: (i) *m*CPBA/MeOH, room temperature, overnight, 4% (**9**), 31% (**10**); (ii) (a) DMTrCl/pyridine, room temperature, overnight, quant.; (b) TBAF/THF, room temperature, overnight, 96%; (iii) (a) NaIO_4_/1,4-dioxne/H_2_O, room temperature, 14 h; (b) NaBH_4_, room temperature, 3 h, 74% over two steps; (iv) BzCl/pyridine/DCM, −78°C, 1 h, 49%; (v) 2-cyanoethyl-*N*,*N*-diisopropyl chlorophosphoramidite/DIPEA/DCM, room temperature, 3 h, 93%.

**Scheme 3.**
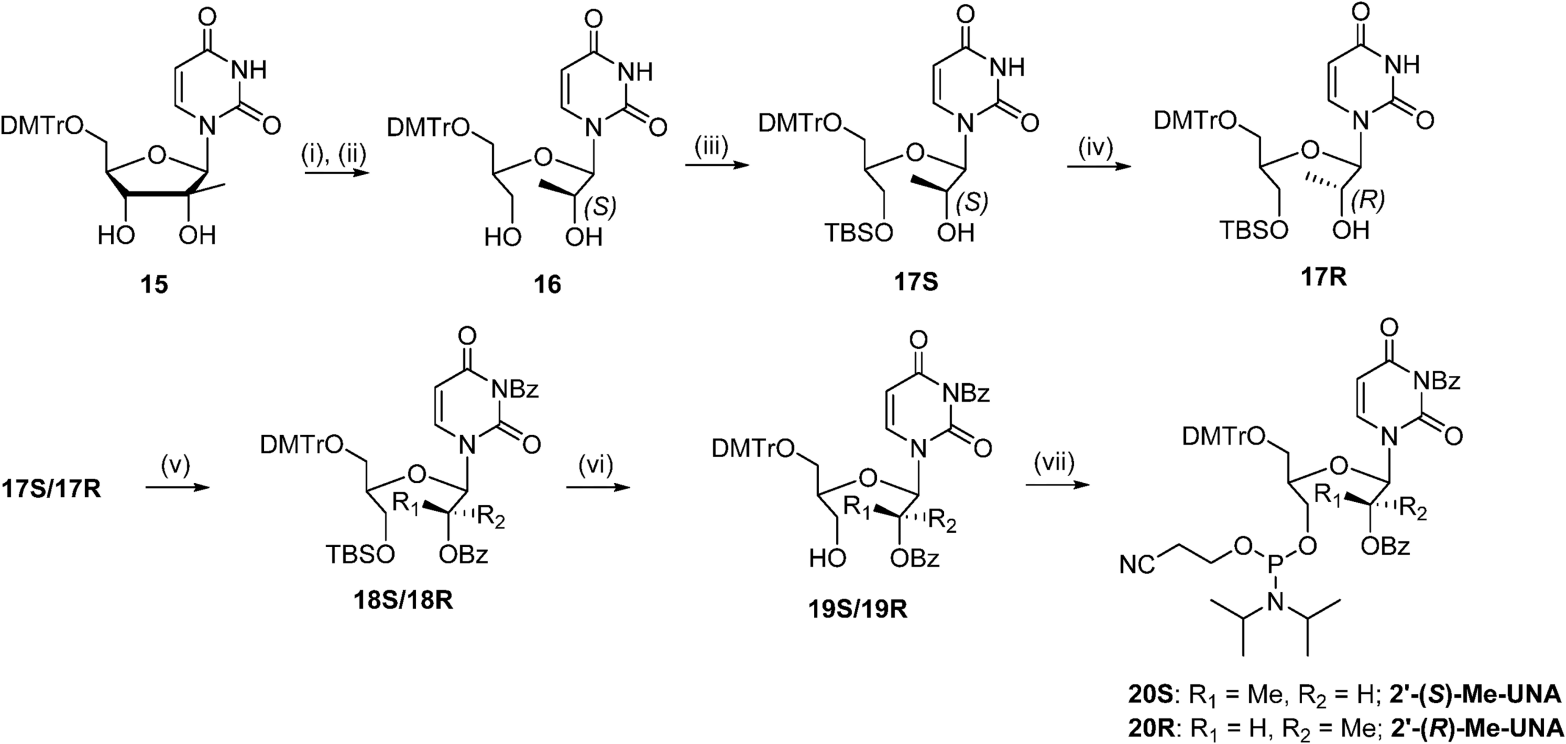
Synthesis of 2′-methyl-UNA uridine building blocks. Reagents and conditions: (i) NaIO_4_/H_2_O/DCM, room temperature, 4 h; (ii) RuCl(p-cymene)[(*S*,*S*)-Ts-DPEN]/HCOONa/H_2_O/EtOAc, room temperature, 12 h, 80% over two steps; (iii) TBSCl/pyridine, room temperature, 3 h, 64%; (iv) BzOH/DIAD/PPh_3_/THF, room temperature, 3 h; NaOH aq., room temperature, 3 h, 80%; (v) BzCl/Et_3_N/DCM, room temperature, 4 h, 90% (**18S**), 92% (**18R**); (vi) NEt_3_·HF/THF, room temperature, 8 h, 96% (**19S**), 97% (**19R**); (vii) 2-cyanoethyl-*N*,*N*-diisopropylchlorophosphoramidite/DIPEA/DCM, 1 h, 87% (**20S**), 80% (**20R**).

**Scheme 4.**
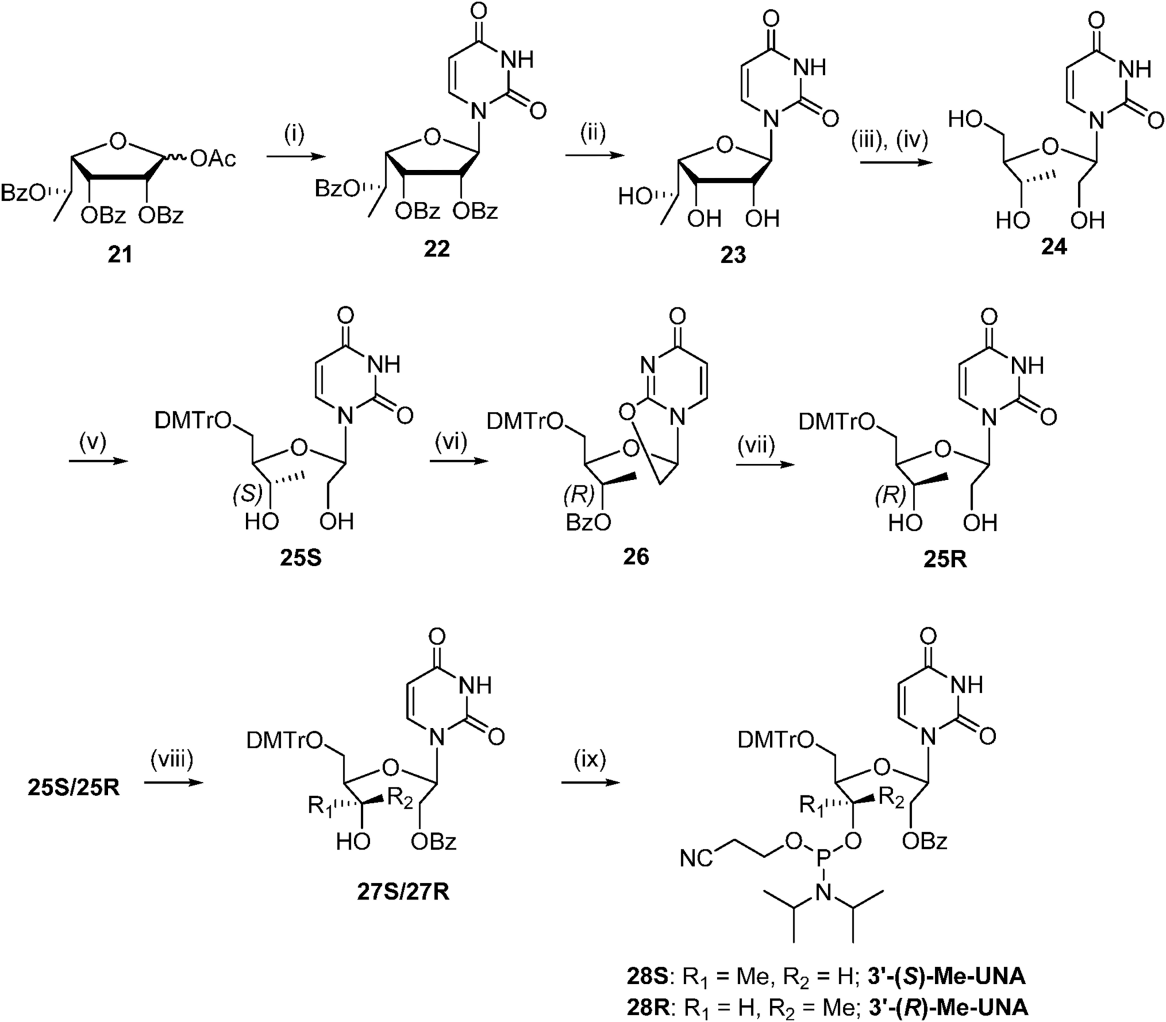
Synthesis of 3′-methyl-UNA uridine phosphoramidites. Reagents and conditions: (i) BSA/TMSOTf/uracil/MeCN, reflux, 1 h, 79%; (ii) NH_3_/MeOH, room temperature, 3 days, 80%; (iii) NaIO_4_/1,4-dioxane/H_2_O, room temperature, 1.5 h; (iv) NaBH_4_, 85% over two steps; (v) DMTrCl/DMAP/pyridine, room temperature, 12 h, 30%; (vi) BzOH/DIAD/PPh_3_/THF, room temperature, 5 h; (vii) NaOH aq., room temperature, 12 h, 77% over two steps (viii) Bz_2_O/DMAP/pyridine, room temperature, 5 h, 80% (**27S**), 84% (**27R**); (ix) 2-cyanoethyl-*N*,*N*-diisopropylchlorophosphoramidite/DIPEA/DCM, room temperature, 1–2 h, 86% (**28S**), 81% (**28R**).

The 5′-methyl-UNA uridine (5′-Me-UNA) phosphoramidites (**7S** and **7R**) were synthesized from commercially available 2′,3′-bis protected nucleosides [1] using Dess-Martin oxidation followed by reductive methylation. The resulting *S* isomer **3S** was inverted to the *R* isomer **3R** via a Mitsunobu reaction. **3S** and **3R** were converted to the UNA structure by an oxidative cleavage reaction, and the phosphoramidites **7S** and **7R** were obtained with a benzoyl group at the 2′ position (Scheme 1). The 4′-*C*-(β)-methoxy-UNA uridine phosphoramidite was synthesized via Prilezhaev epoxidation followed by ring opening. The resulting two isomers were separated and the β isomer **10** was converted to the 2′-Bz-protected UNA phosphoramidite **14** (Scheme 2). The 2′-methyl-UNA uridine (2′-Me-UNA) phosphoramidites (**20S** and **20R**) were obtained from the structurally defined 2′-methyl uridine analogue **15**. NalO_4_-mediated oxidative cleavage of the diol and Noyori asymmetric hydrogenation provided the stereo-pure *S* isomer **17S**. The remaining secondary alcohol was inverted to the *R* isomer by a Mitsunobu reaction to yield **17R**. **17R** and **17S** were converted to the phosphoramidites (Scheme 3). The configurations of 2′-Me-UNA enantiomers were determined by Mosher ester analysis (see below). The 3′-methyl-UNA uridine (3′-Me-UNA) phosphoramidites (**28S** and **28R**) were obtained from stereo-defined functionalized L-rhamnofuranose (compound **21**). The *S* isomer of 3′-Me-UNA (compound **24**) was obtained via glycosidation and diol oxidative cleavage. The *R* isomer was obtained via secondary hydroxyl inversion, and both isomers were converted to phosphoramidites (Scheme 4). All the phosphoramidite building blocks were site-specifically incorporated into oligonucleotides using an automated synthesizer. Cleavage from the solid support and subsequent deprotection of the synthesized oligonucleotides were performed under standard conditions using aqueous methylamine. The crude oligonucleotides were purified by analytical anion exchange chromatography and characterized by mass spectroscopy (see Supporting Information for details).

### Mosher ester analysis assignment of *R* and *S* configurations of 2′-methyl-UNAs

**Figure 2.**
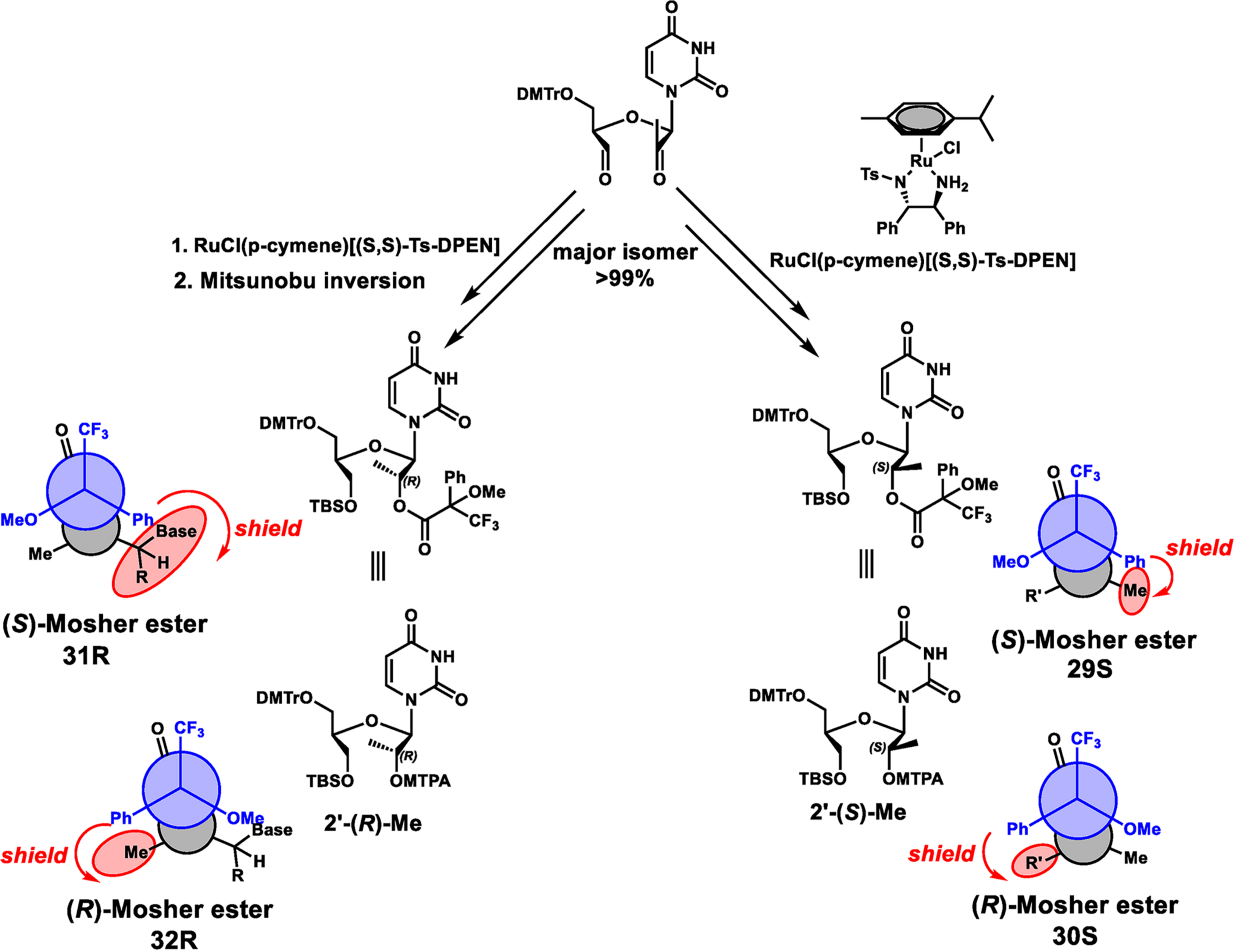
Conformations of the (*S*)- and (*R*)-MTPA ester pairs **29S** and **30S** and pairs **31R** and **32R**. Atomic structures and Newman projections are shown.

**Scheme 5.**
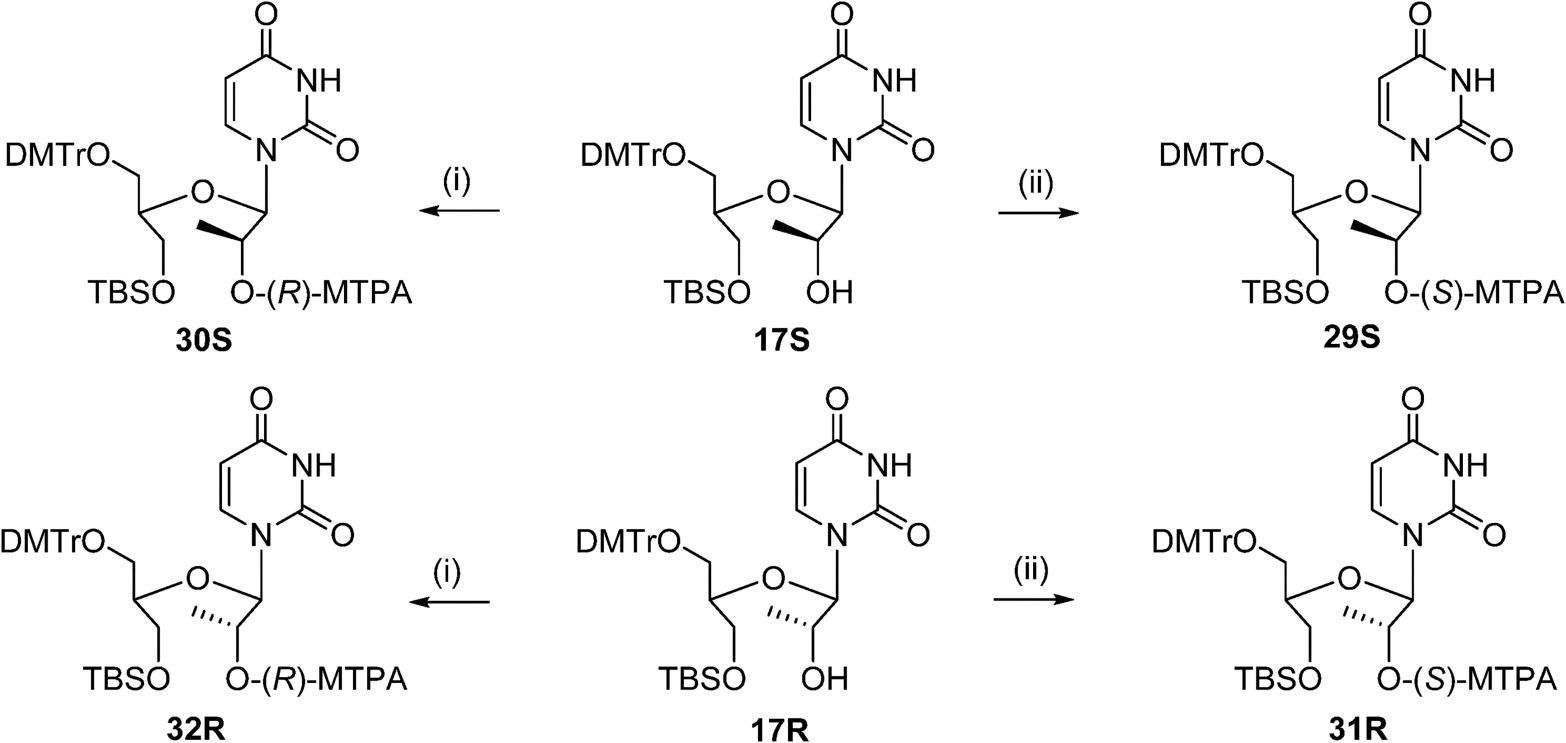
Synthesis of 2′-methyl-UNA uridine Mosher esters. Reagents and conditions: (i) (*S*)-MTPACl/DMAP/Et_3_N/MeCN, room temperature, 5 h, 52–77%; (ii) (*R*)-MTPACl/DMAP/Et_3_N/MeCN, room temperature, 5 h, 54–78%.

To determine the absolute configurations of the 2′ positions of the modified UNAs, we used an NMR-based Mosher ester analysis similar to that described previously [41]. We coupled the 2′-hydroxyl groups of **17S** and of **17R** separately with (*R*)-(−)-MTPACl and (*S*)-(+)-MTPACl. This resulted in the formation of Mosher esters **29S** and **30S** for compound **17S** and Mosher esters **31R** and **32R** for compound **17R**. The phenyl substituent of the MTPA ester imposes an anisotropic, magnetic shielding effect on protons residing above and below the plane of the phenyl ring. This shielding results in an upfield chemical shift for the affected protons in the NMR spectrum. The ^1^H NMR chemical shift differences (δ*^SR^*) for the 1′ proton and the 2′-methyl protons in the Mosher ester pairs were obtained (Table 1). For the Mosher ester pair derived from compound **17S** (compounds **29S** and **30S**), the δ*^SR^* values for nucleobase proton and 1′ proton were positive, and the δ*^SR^* value for the 2′-methyl protons was negative. This result indicates that in compound **29S**, the 2′-methyl group is on the same side as the phenyl group, whereas the nucleobase proton and the 1′ proton are on the opposite side. In compound **30S**, the converse was observed: Results indicated that the nucleobase proton and 1′ proton were on the same side as the phenyl group, whereas the 2′-methyl group was on the opposite side. For the Mosher ester pair derived from compound **17R** (compounds **31R** and **32R**), the δ*^SR^* value for the 2′-methyl proton was positive, and the δ*^SR^* value for the nucleobase proton and 1′ proton were negative. Thus, **17S** has the 2′-(*S*)-methyl configuration and **17R** has the 2′-(*R*)-methyl configuration (Fig. 2 and Scheme 5).

**Table 1.** Δδ*^SR^* data for the (*S*)- and (*R*)-MTPA esters of 2′-methyl-UNA

				Δδ*^SR^=* (δ*_S_*− δ*_R_*)
Proton	*d* (*S*)-Mosher ester	*d* (*R*)-Mosher ester	ppm	Hz (400 MHz)
compound **17S**				
3NH	11.48	11.44	0.04	16
1′H	5.88	5.82	0.02	8
2′Me	1.20	1.31	−0.11	−44
compound **17R**				
3NH	11.28	11.47	−0.19	−76
1′H	5.89	5.98	−0.09	−36
2′Me	1.39	1.30	0.09	36

### Modified UNA destabilizes an RNA:RNA duplex

Melting temperatures (*T*_m_) of hybridization of 12-mer RNA duplexes containing a single, centrally located modification were evaluated. Duplexes containing modified UNAs had similar or slightly decreased *T*_m_ values compared to the RNA duplex containing a single UNA (Table 2).

**Table 2.** Melting temperatures and extent of RNA:RNA duplex destabilization by modified UNA^a^

ID	Sequence 5′-UACAG**X**CUAUGU-3′	*T*_m_ [°C]	(Δ*T*_m_) [°C]
**ON 1**	X = U	61.1	
**ON 4**	X = UNA	43.6	−17.5
**ON 5**	X = 5′-(*S*)-Me-UNA	43.3	−17.8
**ON 6**	X = 5′-(R)-Me-UNA	42.5	−18.6
**ON 7**	X = 2′-(S)-Me-UNA	42.4	−18.7
**ON 8**	X = 2′-(R)-Me-UNA	42.6	−18.5
**ON 9**	X = 3′-(S)-Me-UNA	42.1	−19.0
**ON 10**	X = 3′-(R)-Me-UNA	43.3	−17.8
**ON 11**	X = 4′-(β)-methoxy-UNA	45.4	−15.7

^a^The absorbances of hybridized duplexes formed by indicated sequence with 3′-AUGUCAGAUACA)-5′ (2.5 μM each strand) at 260 nm were determined as a function of temperature in PBS. The *T*_m_ was determined as the maximum of the first derivative of the melting curve. Values are reported as the average of two independent experiments. Δ*T*_m_ was calculated with respect to the unmodified RNA duplex. Reported values are an average of six determinations using the Varian Cary Bio-300 built-in software, with standard deviation reported.

### Modified UNAs impart stability against exonuclease degradation

**Figure 3.**
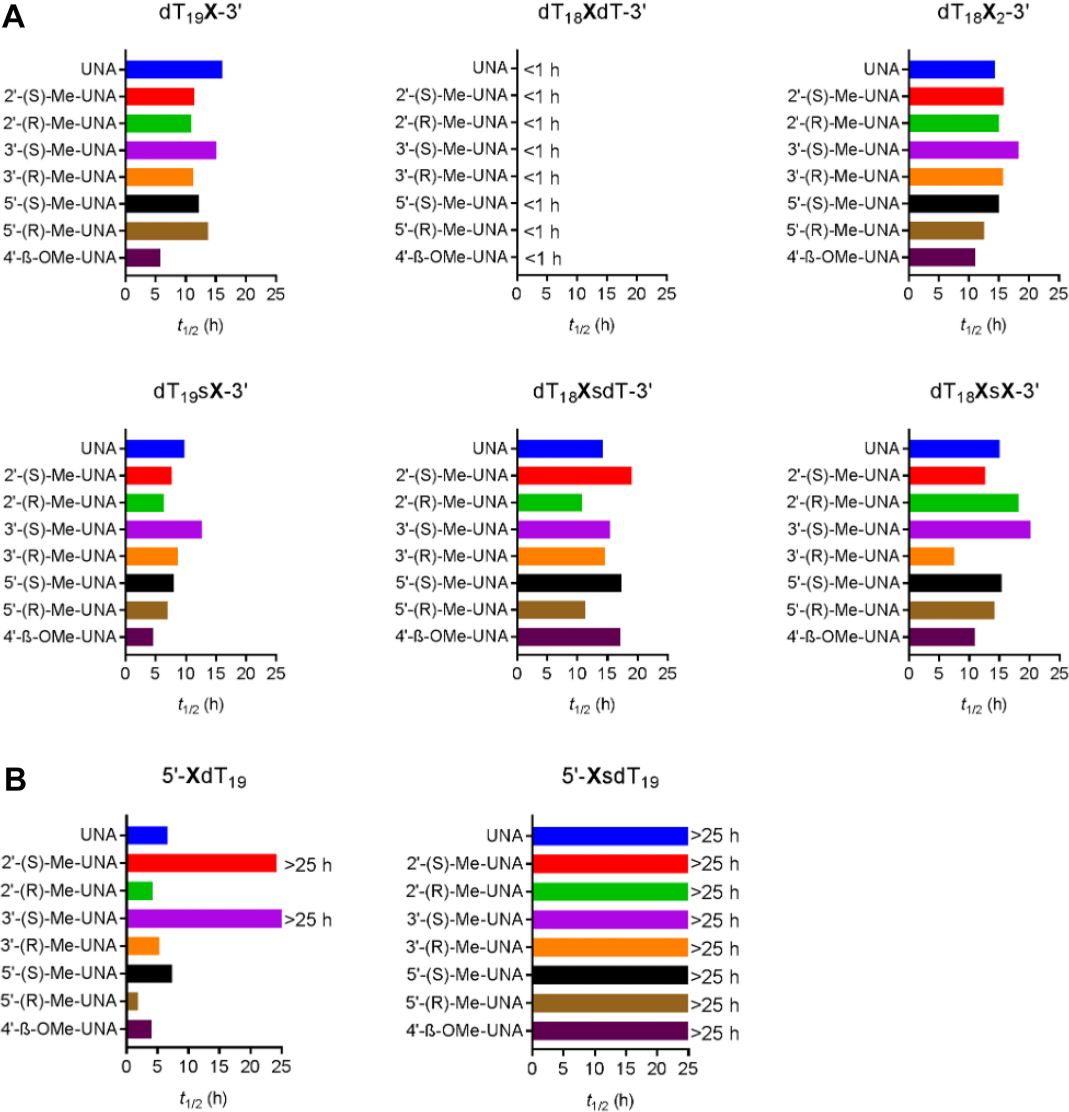
Half-lives of modified oligonucleotides in the presence of either 5′- or 3′-exonuclease. Oligonucleotides (0.1 mg/mL) were incubated with (**A**) 150 mU/mL SVPD in 50 mM Tris, pH 7.2, 10 mM MgCl_2_ or (**B**) 500 mU/mL PDE II in 50 mM sodium acetate buffer (pH 6.5) with 10 mM MgCl_2_. Full-length product was quantified IEX-HPLC. ON12, 5′-dT_19_X-3′; ON13, 5′-dT_18_XdT-3′; ON14, 5′-dT_18_X_2_-3′; ON15, 5′-dT_19_sX-3′; ON16, 5′-dT_18_XsdT-3′; ON17, 5′-dT_18_XsX-3′; ON18, 5′-XdT_19_-3′; and ON19, 5′-XsdT_19_-3′. The “s” indicates a PS linkage. All other linkages were phosphodiester.

To assess the impact of modified UNA on metabolic stability, terminally modified poly-dT oligonucleotides were incubated in the presence of either a 3′- or 5′-exonuclease. In the presence of 3′-exonuclease SVPD, the stabilities of the oligonucleotides containing modified UNAs were comparable to those containing unmodified UNA regardless of stereochemistry or position for a single or double terminal incorporation in a phosphodiester backbone (Fig. 3A). Introduction of a PS linkage at the 3′ end of the modified oligonucleotides (dT_18_ **X**sdT) led to improved stability compared to phosphodiester counterparts (Fig. 3A). The stability of 5′-modified oligonucleotides against degradation mediated by the 5′ PDE-II was also evaluated. Oligonucleotides with phosphodiester backbones containing single UNA residue at the terminus were quite stable in the presence of PDE-II (Fig. 3B). Compared to standard assay conditions, which result in considerable degradation of an oligonucleotide with a 5′ terminal DNA phosphorothioate, as the acyclic UNA backbone is quite stable toward PDE-II, a 10-fold increase in the concentration of PDE-II was required to evaluate the stabilities. The oligonucleotides containing 2′-(*S*)-Me-UNA and 3′-(*S*)-Me-UNA were considerably more resistant than the other oligonucleotides tested to PDE-II-catalyzed degradation than the oligonucleotide with a UNA, possibly due to the steric hinderance. The PS backbone modification provided an additional stabilizing effect.

### Modified UNAs mitigate off-target activity without reducing on-target activity in cell culture

**Figure 4.**
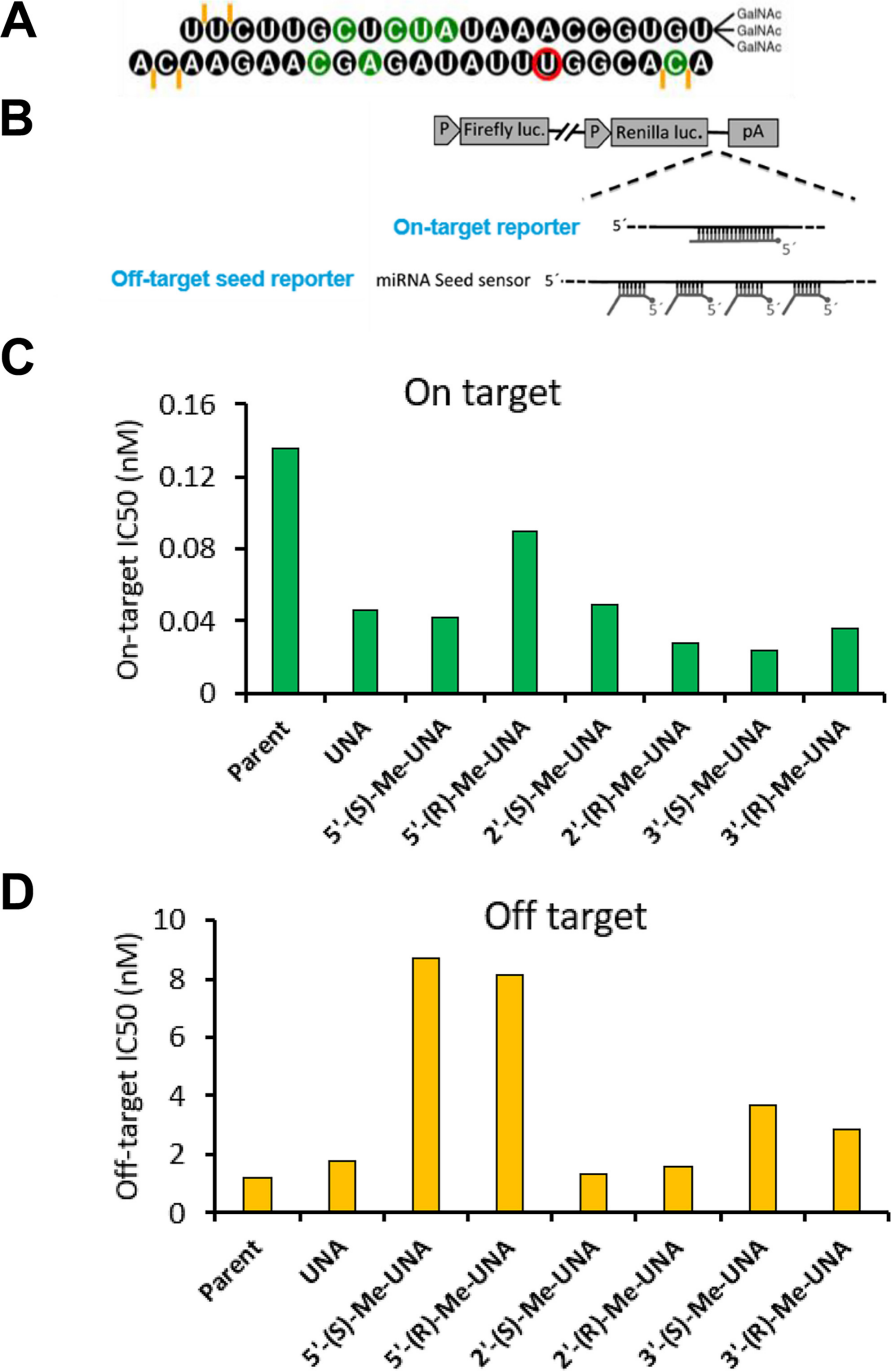
Seed-mediated off-target activity is mitigated by incorporation of UNA or modified UNA at position 7 of the antisense strand. (**A**) Bead diagram of the siRNA used in the experiment. 2′-F and 2′-OMe nucleotides are indicated in green and black, respectively. PS linkages are indicated by orange lines. (**B**) Schematic of reporters used to evaluate on- and off-target effects. Reporters were expressed in Cos7 cells, and *Renilla* luciferase signal was measured at 48 h after transfection with siRNA (50 nM - 0.64 pM). *Renilla* luciferase signal was normalized to signal from cells treated with non-targeting siRNA. (**C**) On-target IC_50_ values (*n* = 3). (**D**) Off-target IC_50_ values (*n* = 3).

Placement of a single UNA residue in the seed region of the antisense strand inhibits miRNA-like, off-target activity without loss of on-target activity [25]. The efficiency of UNA-mediated inhibition of off-target activity was greater when the UNA was located in positions 5, 6, or 7 than in positions 1, 2, or 3 [42]. We incorporated modified UNAs at position 7 of the antisense strand of an siRNA targeting *Ttr* (Fig. 4A) and measured the IC_50_ values for on- and off-target activities using a luciferase reporter assay that has been previously described [18, 19, 28] (Fig. 4B). For evaluation of on-target silencing, the 23-mer siRNA target site was cloned into the 3′-UTR of *Renilla* luciferase. For measurement of off-target gene silencing, four tandem repeats of a sequence complementary to antisense positions 2–9 were cloned into the 3′-UTR of *Renilla* luciferase. The siRNAs containing UNA or modified UNAs reduced on-target potencies slightly compared to that of the parent siRNA (Fig. 4C, Table 3). The off-target activity was considerably mitigated by incorporating a single *R* or *S* isomer of 5′-Me-UNA at position 7 (Fig. 4D, Table 3). The off-target to on-target ratios were calculated by dividing the off-target IC_50_ values by the on-target IC_50_ values (Table 3); the larger this ratio, the better the off-target mitigation. The 5′-(*S*)-Me-UNA had a very high off-target to on-target ratio and reduced potency relative to the parent only slightly. This modification may conformationally pre-organize the siRNA antisense strand and facilitate the local kink in the seed region when the RNA is bound by AGO2.

**Table 3.** On- and off-target effects of siRNAs containing UNA or modified UNA^a^

siRNA	Sequence (5′→3′)	on-target IC_50_ (pM)	off-target IC_50_ (nM)	off-target/ on-target ratio
**si1**	u•u•cuugCuCUAuaaaccgugu*	136	1.21	9
	a•C•acgguuuauagAgCaagaa•c•a			
**si2**	u•u•cuugCuCUAuaaaccgugu*	46.3	1.80	39
	a•C•acgg***U***uuauagAgCaagaa•c•a			
**si3**	u•u•cuugCuCUAuaaaccgugu*	42.1	8.73	207
	a•C•acgg***U_5__′__S_***uuauagAgCaagaa•c•a			
**si4**	u•u•cuugCuCUAuaaaccgugu*	89.8	8.13	90
	a•C•acgg***U_5__′__R_***uuauagAgCaagaa•c•a			
**si5**	u•u•cuugCuCUAuaaaccgugu*	48.6	1.35	28
	a•C•acgg***U_2__′__S_***uuauagAgCaagaa•c•a			
**si6**	u•u•cuugCuCUAuaaaccgugu*	27.7	1.61	58
	a•C•acgg***U_2__′__R_***uuauagAgCaagaa•c•a			
**si7**	u•u•cuugCuCUAuaaaccgugu*	23.9	3.65	152
	a•C•acgg***U_3__′__S_***uuauagAgCaagaa•c•a			
**si8**	u•u•cuugCuCUAuaaaccgugu*	35.8	2.86	80
	a•C•acgg***U_3__′__R_***uuauagAgCaagaa•c•a			

^a^On- and off-target IC_50_ values were measured using luciferase reporter assays in COS-7 cells. For experimental conditions, see Fig. 4. Here, uppercase letters, 2′-F nucleotides; lowercase letters, 2′-OMe nucleotides; ***U***, UNA; ***U_5__′__S_***, 5′-(*S*)-Me-UNA; ***U_5__′__R_***, 5′-(*R*)-Me-UNA; ***U_2__′__S_***, 2′-(*S*)-Me-UNA; ***U_2__′__R_***, 2′-(*R*)-Me-UNA; ***U_3__′__S_***, 3′-(*S*)-Me-UNA; ***U_3__′__R_***, 3′-(*R*)-Me-UNA; ***U_4__′__β_***, 4′-(β)-methoxy-UNA;• phosphorothioate linkages and *, trivalent GalNAc ligand

### siRNAs containing modified UNA induce silencing in mice

Next, the activities of siRNAs containing with UNA or modified UNA at position 7 of the antisense strand were evaluated in mice. These siRNAs are 21:23-mer asymmetric duplexes modified with 2′-OMe and 2′-F nucleotides, terminal phosphorothioates, and, for delivery via subcutaneous administration, the sense strands of the siRNAs were conjugated to GalNAc-conjugated siRNA. The siRNAs target *Ttr*. Melting temperatures were evaluated. Incorporation of modified UNAs were destabilizing (Table 4); extents of destabilization due to different UNA modifications for these 21/23mer duplexes were smaller to those observed in the context of 12-mer duplexes as expected (Table 2).

**Table 4.** Sequences of siRNAs used for *in vivo* study and *T*_m_ values

siRNA	Modification at position 7 of antisense strand	Sequences (5′ -3′)^a^	*T*_m_ [°C]^b^	Δ*T*_m_ [°C]^c^
**si9**	-	a•g•uguuCuUGCucuauaaaca*	87.27	-
		u•G•uuuauagagcaAgAacacu•g•u		
**si10**	UNA	a•g•uguuCuUGCucuauaaaca*	81.97	-5.30
		u•G•uuua***U***agagcaAgAacacu•g•u		
**si11**	5′-(*S*)-Me-UNA	a•g•uguuCuUGCucuauaaaca*	81.97	-5.30
		u•G•uuua***U_5__′__S_***agagcaAgAacacu•g•u		
**si12**	5′-(*R*)-Me-UNA	a•g•uguuCuUGCucuauaaaca*	82.02	-5.25
		u•G•uuua***U_5__′__R_***agagcaAgAacacu•g•u		
**si13**	2′-(*S*)-Me-UNA	a•g•uguuCuUGCucuauaaaca*	83.02	-4.25
		u•G•uuua***U_2__′__S_***agagcaAgAacacu•g•u		
**si14**	2′-(*R*)-Me-UNA	a•g•uguuCuUGCucuauaaaca*	82.12	-5.15
		u•G•uuua***U_2__′__R_*** agagcaAgAacacu•g•u		
**si15**	3′-(*S*)-Me-UNA	a•g•uguuCuUGCucuauaaaca*	81.97	-5.30
		u•G•uuua***U_3__′__S_*** agagcaAgAacacu•g•u		
**si16**	3′-(*R*)-Me-UNA	a•g•uguuCuUGCucuauaaaca*	81.97	-5.30
		u•G•uuua***U_3__′__R_*** agagcaAgAacacu•g•u		
**si17**	4′-(β)-OMe-UNA	a•g•uguuCuUGCucuauaaaca*	82.97	-4.30
		u•G•uuua***U_4__′__b_***agagcaAgAacacu•g•u		

^a^Uppercase letters, 2′-F nucleotides; lowercase letters, 2′-OMe nucleotides; *U*, UNA; *U_5__′__S_*, 5′-(*S*)-Me-UNA; *U_5__′__R_*, 5′-(*R*)-Me-UNA; *U_2__′__S_*, 2′-(*S*)-Me-UNA; *U_2__′__R_*, 2′-(*R*)-Me-UNA; *U_3__′__S_*, 3′-(*S*)-Me-UNA; *U_3__′__R_*, 3′-(*R*)-Me-UNA; *U_4__′__β_*, 4′-(β)-methoxy-UNA;• phosphorothioate linkages; *, trivalent GalNAc ligand.

^b^The absorbances of hybridized duplexes (2.5 μM) at 260 nm were determined as a function of temperature in PBS. The *T*_m_ was determined as the maximum of the first derivative of the melting curve. Values are reported as the average of two independent experiments.

^c^Δ*T*_m_ was calculated with respect to si9.

**Figure 5.**
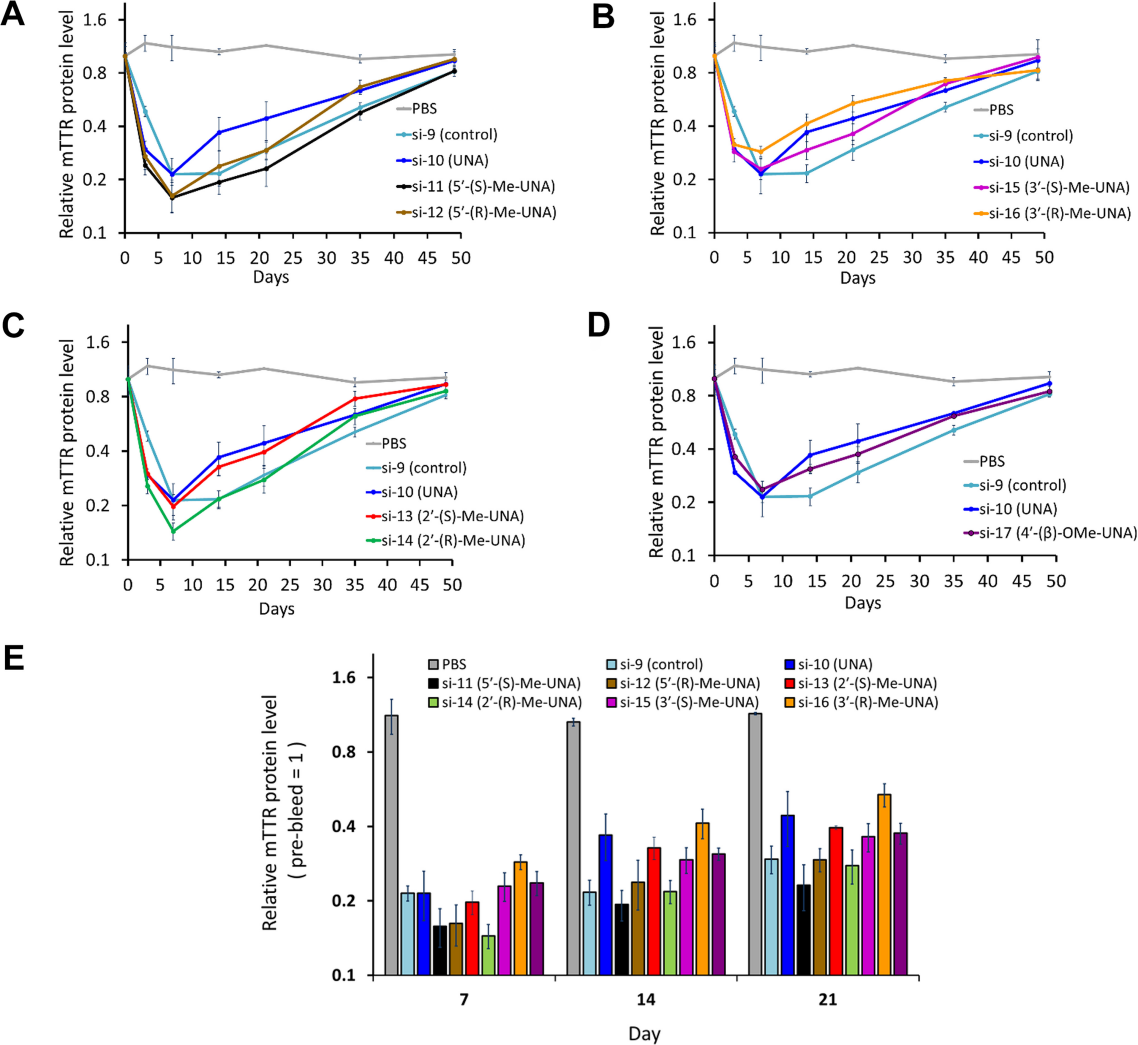
The siRNA modified with 5′-(*S*)-Me-UNA has higher potency than other siRNAs tested. (**A-D**) TTR protein levels relative to pre-dose levels in mice treated with a single dose of 1.0 mg/kg siRNA modified with A) 5′-Me-UNA, B) 3′-Me-UNA, C) 2′-Me-UNA, and D) 4′-β-methoxy-UNA. TTR was quantified in serum samples using a sandwich ELISA assay. All samples were tested in duplicate in the same experiment. In these plots, samples are grouped to facilitate comparison of isomers; data for controls PBS, **si9** (parent), and **si10** (UNA modified) are shown in each panel. Each data point is the average of the mice in each cohort (*n* = 3). (**E**) The relative protein TTR protein level on day 7 indicates the nadir for all the modified siRNA including the control duplex **si9**. Interestingly, here 2′-(*R*)-Me-UNA containing **si14** showed slightly better potency than 5′-(*S*) and (*R*)- Me-UNA modified duplexes **si11** and **si12**, respectively. However, the difference becomes clear on days 14 and 21, respectively, where 5′-(*S*)-Me-UNA showed better efficacy over the 2′ analogue.

In mice, at a dose of 1.0 mg/kg, the parent siRNA with a 2′-OMe at antisense strand position 7 (**si9**) decreased levels of circulating TTR protein by about 80% compared to pre-dose levels at 7 days post-administration. Incorporation of a UNA at position 7 of the antisense strand (**si10**) resulted in activity similar to the parent at day 7 post treatment; however, the duration of action was slightly reduced (Fig. 5A). The siRNA modified with the 5′-(*S*)-Me-UNA (**si11**) was more potent than the parent **si9** on day 7 and had comparable duration to the parental siRNA (Fig. 5A). The 5′-(*R*)-Me-UNA isomer-modified siRNA **si12** had similar potency on day 7 but shorter duration of action. The *S* isomer of the 3′-Me-UNA imparted higher potency than the *R* isomer (Fig. 5B). Interestingly, examination of the relative TTR levels on day 7, the nadir for the control duplex **si9**, revealed that 2′-(*R*)-Me-UNA-containing **si14** appeared to have higher potency than the other siRNAs tested, including the 2′-(*S*)-Me-UNA-modified siRNA **si13** (Fig. 5C). It is possible that **si9** may be less metabolically stable than the UNA-containing siRNAs, although we do not have data to confirm this speculation. It should be noted that we did not perform sufficient replicates to evaluate statistical significance. These siRNAs will need to be evaluated at lower doses to confirm these trends. The *in vitro* and *in vivo* results were in agreement as observed previously [25].

Using **si17**, we have evaluated the (*S*)-4′-OMe isomer of UNA. In the past, we have evaluated both 4′-*C*α-OMe, (the *R* isomer) and 4′-*C*β-OMe (the *S* isomer) epimers (with 2′-F RNA) for RNAi mediated silencing activities and found them to be different: The 4′-*C*β-OMe epimer reduced the potency of the siRNA when incorporated into the siRNAs significantly more compared to 4′-*C*α-OMe epimer [43]. In the present synthesis (Scheme 2, with 2'-OH) the 4′-*C*β-OMe, the (*S*) isomer is the major product and the other one was minor and difficult to purify. Based on these observations, in the present work, we have evaluated the off-target mitigation potential and *in vivo* silencing of (*S*)-4′-OMe UNA isomer only.

### Structural and functional consequences of *S* and *R* isomers of methylated UNAs

To gain insights into the origins of differences in potencies and durations of action of siRNAs containing modified UNA, we used computational modeling to visualize the interactions between UNA and modified UNA and AGO2 residues. We used coordinates of the complex between AGO2 and miR-20a (PDB ID 4f3t) [44] and replaced the residue at position 7 of the antisense strand with UNA using the UCSF Chimera suite [45]. Subsequently, the model was energy-minimized with Amber [46] as implemented in UCSF Chimera using steepest descent and conjugate gradient algorithms until convergence. The destabilization caused by UNA in the context of an isolated duplex suggested that there might be a drastic change in conformation relative to RNA. However, the UNA in the grip of AGO2 residues had only minor changes in conformation relative to this position in miR-20a (Supplementary Fig. S1). The inter-phosphate distance is 5.7 Å for the UNA compared to 5.5 Å in the parent miR-20a strand. The kink in the antisense strand at that site is stabilized by three arginines, Arg-375, Arg-714, and Arg-761. It is likely that the more flexible UNA backbone facilitates formation of the kink. The 2′ hydroxyl groups of both UNA and RNA engage in hydrogen bonds to the main chain oxygen of Ala-221 and the side chain Oγ of Thr-368 (Supplementary Fig. S1).

**Figure 6.**
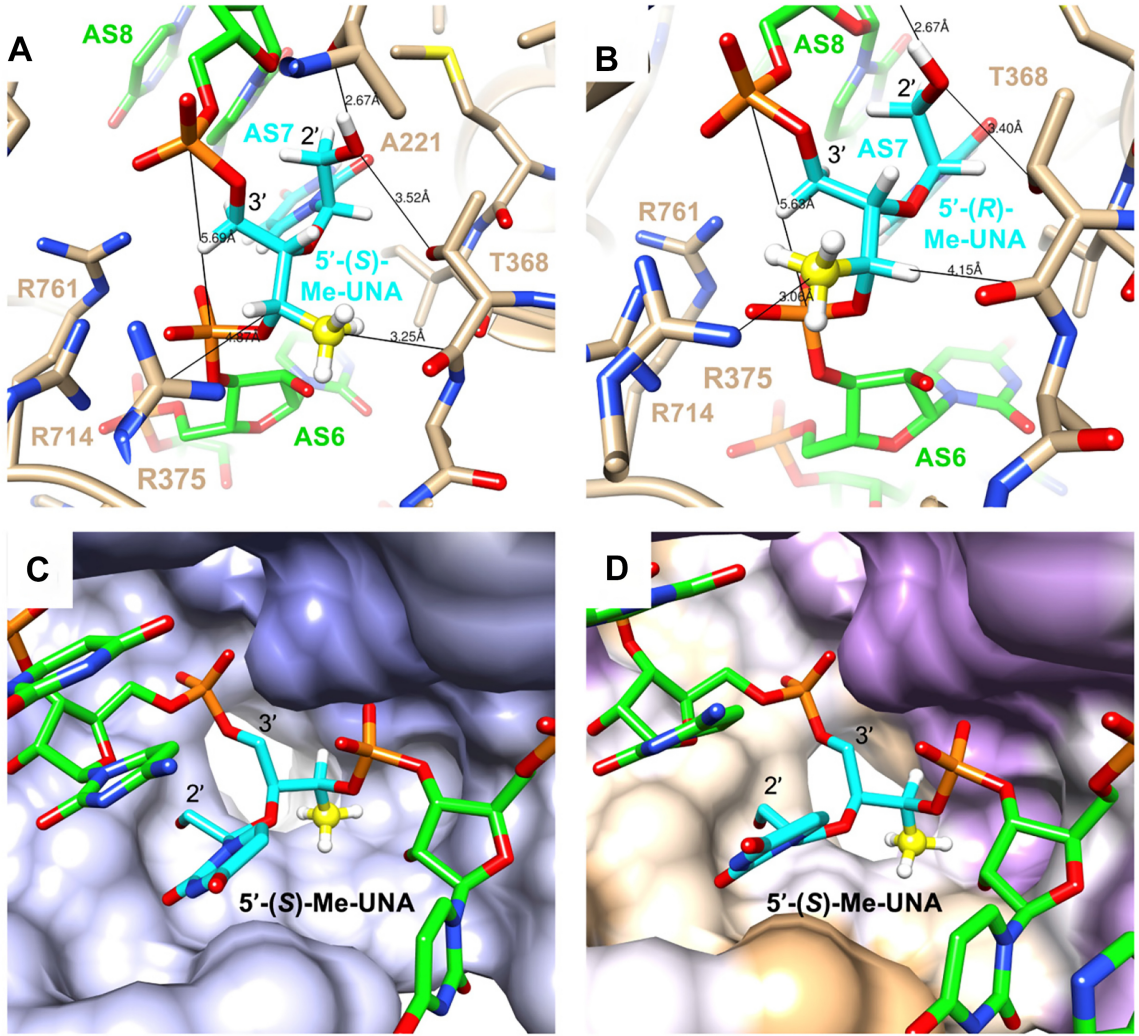
Computationally modeled structures of siRNAs modified with 5′-(*S*)-Me-UNA and 5′-(*R*)-Me-UNA in the AGO2 binding site. (**A**and**B**) Conformation and Ago2 interactions of **A**) 5′-(*S*)-Me-UNA-U and **B**) 5′-(*R*)-Me-UNA-U. Carbon atoms of modified UNA residues are colored in cyan, methyl groups are highlighted in ball-and-stick mode, and selected distances are indicated by thin solid lines. (**C**) AGO2 surface around 5′-(*S*)-Me-UNA colored according to Coulombic potential: blue positive and white neutral. (**D**) AGO2 surface around 5′-(*S*)-Me-UNA-U colored according to hydrophobic potential: purple low, white neutral, and orange high.

Next, we modeled interaction of AGO2 with siRNAs containing 5′-(*R*)-Me-UNA and 5′-(*S*)-Me-UNA at position 7 of the antisense strand. For the *S* isomer, the methyl group points toward the Cα carbon of Thr-368 (C-C distance of 3.7 Å) (Fig. 6A). For the *R* isomer, the methyl group points toward the guanidino moiety of Arg-375 (C-N distance of 3.1 Å) such that it is partly wedged between phosphates that are tightly spaced due to the kink (Fig. 6B). The 5′-(*S*)-methyl group points away from the hinge and thus does not interfere with formation of the kink in the antisense strand between positions 6 and 7. The starkly different environments of the 5′-(*S*)- and 5′-(*R*)-methyl groups in the AGO2 binding site can be illustrated by coloring the protein surface according to Coulombic potential (Fig. 6C) and hydrophobic potential (Fig. 6D). Consistent with the higher potency of the siRNA modified with the 5′-(*S*)-Me-UNA isomer (**si11**) compared to that of the siRNA modified with the 5′-(*R*)-Me-UNA (**si12**) in mice (Fig. 5A), the fit for methyl moiety of the *R* isomer is clearly worse than that of the *S* isomer.

**Figure 7.**
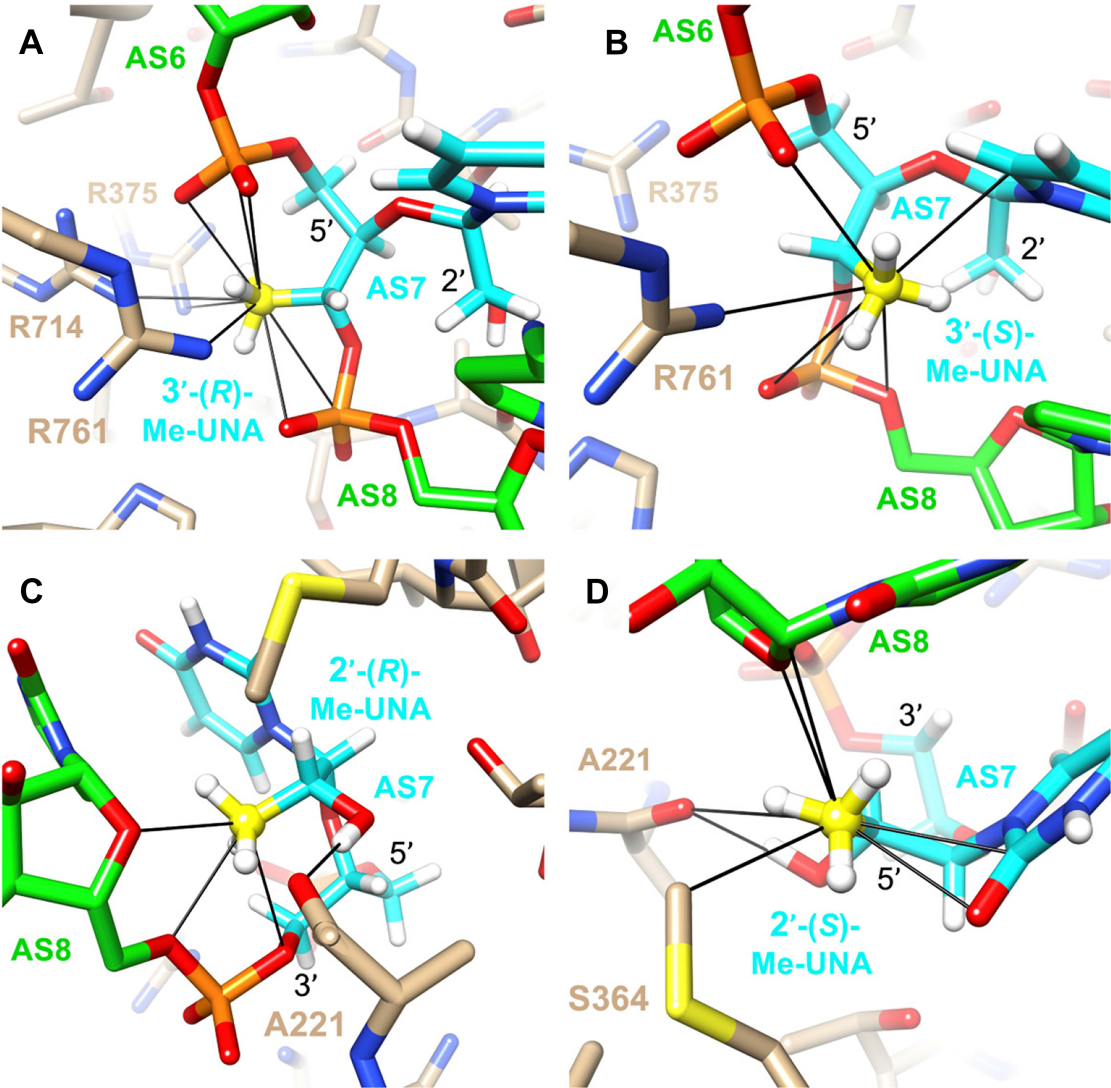
Conformations and AGO2 interactions of (**A**) 3′-(*R*)-Me-UNA-U, (**B**) 3′-(*S*)-Me-UNA-U, (**C**) 2′-(*R*)-Me-UNA-U, and (**D**) 2′-(*S*)-Me-UNA-U. Carbon atoms of modified UNA residues are colored in cyan, methyl groups are highlighted in ball-and-stick mode, contacts < 3.5 Å for methyl groups along with the 2′-OH…O (Ala-221) hydrogen bonds are indicated by thin solid lines, and selected residues are labeled.

The 3′-(*R*)-methyl group is wedged between the phosphate groups at positions 7 and 8 (P…P distance 5.7 Å, Fig. 6A), resulting in short contacts and the intrusion of a hydrophobic moiety into an environment dominated by electrostatics with phosphate groups surrounded by three arginine side chains (Fig. 7A). By comparison, the 3′-(*S*)-methyl group has fewer steric and electrostatic problems consistent with its higher potency (Fig. 5B).

The 2′-(*R*)-Me-UNA-modified **si14** was more potent than the 2′-(*S*)-Me-UNA-modified **si13** (Fig. 5C). Modeling indicated no steric issues between the 2′-(*R*)-methyl group and atoms of the backbone and the protein (Fig. 7C), whereas the 2′-(*S*)-methyl group is trapped between the nucleobase of the residue at position 7 and the ribose of position 8 (Fig. 7D). Rotation around the C1′-C2′ bond might relieve the steric conflict with the sugar but will also sacrifice the hydrogen bond between the 2′-hydroxyl group and the keto group of Ala-221. Thus, the models provide insights into the differences in potencies of siRNAs containing *R* and *S* isomers of modified UNAs observed in mice.

There were striking differences in the half-lives of strands carrying at their 5′ ends either *R* or *S* isomers of 2′-Me-UNA and 3′-Me-UNA (Fig. 2B). The *t*_1/2_ values of *S* isomers for both 2′- and 3′-mUNA-protected 20-mers were greater than 25 h, whereas the corresponding oligonucleotides with the *R* isomers have *t*_1/2_ values of less than 5 h. To gain a better understanding of the origins of these differences, we built models of complexes of UNA-modified trimers with *Drosophila melanogaster* 5′-3′ exoribonuclease Xrn1 based on the crystal structure of Xrn1 bound to a 5′-phosphorylated trinucleotide P-d(TTT) (PDB ID 2y35) [47].

**Figure 8.**
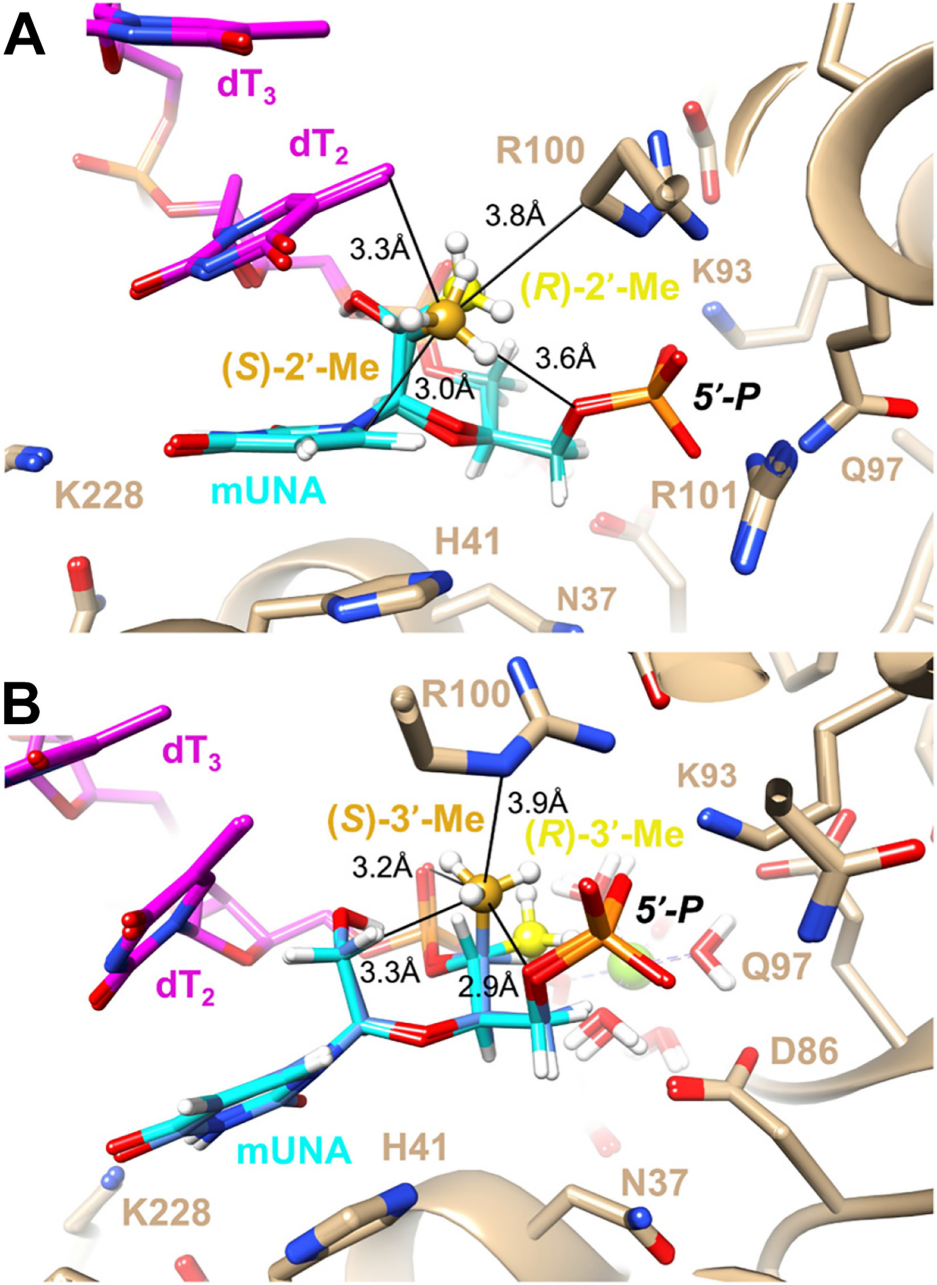
Computational models of the Xrn1 active site bound to mUNA-dTdT trimers reveal differences between *R* and *S* isomers of (**A**) 2′-(*S*)- and 2′-(*R*)-Me-UNA-U and (**B**) 3′-(*S*)- and 3′-(*R*)-Me-UNA-U. Methyl groups are highlighted in ball-and-stick mode, with the carbon of the (*S*) and (*R*) isomers colored in golden and yellow, respectively. Selected residues are labeled, close contacts are shown with thin solid lines and distances in Å, and a Mg^2+^ is visible as a green sphere behind the 5′-phosphate in panel B.

The energy minimized models of Xrn1 complexes with these trimers show distinct orientations of the *S*- and *R*-methyl groups (Fig. 8). In all cases, methyl groups are distal to Mg^2+^ ions; metal ions in the Xrn1 active site do not form inner sphere contacts with phosphate groups of the terminal nucleotide. Therefore, the observed differences in resistance between isomers are unlikely to be the result of a steric interference between methyl and metal ion. However, it appears that *S*-methyl groups cause more steric strain than *R*-methyl groups. In the case of 2′-Me-UNA, the *S*-methyl group is partly wedged between the nucleobases of the terminal and penultimate residues. Thus, it appears to push apart the base moieties and the shortest distances between methyl carbon and base atoms are around 3 Å (Fig. 8A). Additional close contracts exist to O5′ and a key residue at the active site, Arg-100; by comparison, the *R*-methyl group shows none of these close interactions (Fig. 8A).

The distinct steric consequences of different orientations of *S*- and *R*-methyl groups are also obvious in the case of the 3′-Me-UNA. The *S*-methyl adopts an axial orientation that results in two tight 1,3-diaxial contacts, one to the OP2 non-bridging oxygen of the 3′-phosphate and one to O5′ of the 5′ phosphate (Fig. 8B). Both may affect the orientations of phosphates, which could influence binding and cleavage by the exonuclease. Additional tight contacts exist between *S*-methyl and C2′ as well as Arg-100. The *R*-methyl group does not exhibit close 1,3-diaxial contacts and is farther removed from both Arg-100 and Lys-93 than the *S*-methyl group (Fig. 8B).

## Conclusions

We explored the effects of flexible modified UNAs, modifications not previously investigated, on the thermal stability, nuclease resistance, and *in vitro* and *in vivo* activities of siRNAs. Modified UNA uridine phosphoramidites were synthesized and incorporated into oligonucleotides. Our studies showed that one or two terminal modified UNAs, regardless of stereochemistry or position, imparted similar stabilities in the presence of 3′-exonuclease as unmodified UNA. Thus, despite the presence of the free hydroxyl group, these thermally destabilizing modifications do not decrease stability in the presence of the 3′-exonuclease. In contrast, there were striking differences in the half-lives of strands with *R* or *S* isomers of 2′-Me-UNA and 3′-Me-UNA in the presence of a 5′-exonuclease. Energy minimized models of Xrn1 complexes with trimers containing modified UNAs revealed distinct orientations of the *S*- and *R*-methyl groups. The *S*-methyl groups appear to cause more steric strain than *R*-methyl groups resulting in longer half-lives for oligonucleotides that terminate in *S* isomers of 2′-Me-UNA and 3′-Me-UNA compared to those with *R* isomers.

Incorporating a single modified UNA into the seed region of siRNA duplexes resulted in similar reductions in levels of *Ttr* mRNA *in vitro* as incorporating a single UNA; however, siRNAs with modified UNA, especially 5′-(*S*)-Me-UNA, in the seed region reduced off-target effects more than incorporation of UNA. The siRNAs with 5′-(*R*)-Me-UNA and with 5′-(*S*)-Me-UNA in the seed region of the antisense strand performed better than siRNA containing UNA or other modified UNA analogs in gene silencing assays in mice. Modeling studies indicated that the flexibility of the UNA and modified UNA facilitate kinking of the antisense strand when incorporated at position 7. That the siRNAs with modified UNA have silencing activities on par with an siRNA with a 2′-OMe analogs at position 7 in mice but have the ability to mitigate off-target activity warrants their further investigation. We plan to synthesize all nucleobase analogs of select modified UNA building blocks to enable further evaluation of their utility in nucleic acid-based therapeutics.

## Supplementary Material

gkaf937_Supplemental_File

## Data Availability

Analytical data of all oligonucleotides are available as Supplementary Table S1. All other data needed to evaluate the conclusions of the paper are present in the paper and Supplementary Data.

## References

[1] Akinc A, Maier MA, Manoharan M et al. The Onpattro story and the clinical translation of nanomedicines containing nucleic acid-based drugs. Nat Nanotechnol. 2019; 14:1084–7. 10.1038/s41565-019-0591-y. 31802031

[2] Chan A, Liebow A, Yasuda M et al. Preclinical development of a subcutaneous ALAS1 RNAi therapeutic for treatment of hepatic porphyrias using circulating RNA quantification. Mol Ther Nucleic Acids. 2015; 4:e263 10.1038/mtna.2015.36. 26528940 PMC4877445

[3] Balwani M, Sardh E, Ventura P et al. Phase 3 trial of RNAi therapeutic givosiran for acute intermittent porphyria. N Engl J Med. 2020; 382:2289–301. 10.1056/NEJMoa1913147. 32521132

[4] Liebow A, Li X, Racie T et al. An investigational RNAi therapeutic targeting glycolate oxidase reduces oxalate production in models of primary hyperoxaluria. JASN. 2017; 28:494–503. 10.1681/ASN.2016030338. 27432743 PMC5280024

[5] Fitzgerald K, White S, Borodovsky A et al. A highly durable RNAi therapeutic inhibitor of PCSK9. N Engl J Med. 2017; 376:41–51. 10.1056/NEJMoa1609243. 27959715 PMC5778873

[6] Ray KK, Wright RS, Kallend D et al. Two Phase 3 trials of inclisiran in patients with elevated LDL cholesterol. N Engl J Med. 2020; 382:1507–19. 10.1056/NEJMoa1912387. 32187462

[7] Raal FJ, Kallend D, Ray KK et al. Inclisiran for the treatment of heterozygous familial hypercholesterolemia. N Engl J Med. 2020; 382:1520–30. 10.1056/NEJMoa1913805. 32197277

[8] Adams D, Tournev IL, Taylor MS et al. Efficacy and safety of vutrisiran for patients with hereditary transthyretin-mediated amyloidosis with polyneuropathy: a randomized clinical trial. Amyloid. 2023; 30:18–26. 10.1080/13506129.2022.2091985. 35875890

[9] Liu A, Zhao J, Shah M et al. Nedosiran, a candidate siRNA drug for the treatment of primary hyperoxaluria: design, development, and clinical studies. ACS Pharmacol Transl Sci. 2022; 5:1007–16. 10.1021/acsptsci.2c00110. 36407951 PMC9667536

[10] Fontana M, Berk JL, Gillmore JD et al. Vutrisiran in patients with transthyretin amyloidosis with cardiomyopathy. N Engl J Med. 2025; 392:33–44. 10.1056/NEJMoa2409134. 39213194

[11] Young G, Srivastava A, Kavakli K et al. Efficacy and safety of fitusiran prophylaxis, an siRNA therapeutic, in a multicenter Phase 3 study (ATLAS-INH) in people with hemophilia A or B, with inhibitors (PwHI). Blood. 2021; 138:4 10.1182/blood-2021-150273.34236429

[12] Young G, Srivastava A, Kavakli K et al. Efficacy and safety of fitusiran prophylaxis in people with haemophilia A or haemophilia B with inhibitors (ATLAS-INH): a multicentre, open-label, randomised phase 3 trial. The Lancet. 2023; 401:1427–37. 10.1016/S0140-6736(23)00284-2. 37003287

[13] Egli M, Manoharan M Re-engineering RNA molecules into therapeutic agents. Acc Chem Res. 2019; 52:1036–47. 10.1021/acs.accounts.8b00650. 30912917

[14] Corey DR, Damha MJ, Manoharan M Challenges and opportunities for nucleic acid therapeutics. Nucleic Acid Ther. 2022; 32:8–13. 10.1089/nat.2021.0085. 34931905 PMC8817707

[15] Egli M, Manoharan M Chemistry, structure and function of approved oligonucleotide therapeutics. Nucleic Acids Res. 2023; 51:2529–73. 10.1093/nar/gkad067. 36881759 PMC10085713

[16] Bumcrot D, Manoharan M, Koteliansky V et al. RNAi therapeutics: a potential new class of pharmaceutical drugs. Nat Chem Biol. 2006; 2:711–9. 10.1038/nchembio839. 17108989 PMC7097247

[17] Nair JK, Willoughby JL, Chan A et al. Multivalent N-acetylgalactosamine-conjugated siRNA localizes in hepatocytes and elicits robust RNAi-mediated gene silencing. J Am Chem Soc. 2014; 136:16958–61. 10.1021/ja505986a. 25434769

[18] Schlegel MK, Foster DJ, Kel’in AV et al. Chirality dependent potency enhancement and structural impact of Glycol nucleic acid Modification on siRNA. J Am Chem Soc. 2017; 139:8537–46. 10.1021/jacs.7b02694. 28570818

[19] Guenther DC, Mori S, Matsuda S et al. Role of a “Magic” Methyl: 2'-Deoxy-2'-α-F-2'-β-C-methyl pyrimidine nucleotides modulate RNA interference activity through synergy with 5'-phosphate mimics and mitigation of off-target effects. J Am Chem Soc. 2022; 144:14517–34. 10.1021/jacs.2c01679. 35921401 PMC9389587

[20] Allerson CR, Sioufi N, Jarres R et al. Fully 2'-modified oligonucleotide duplexes with improved in vitro potency and stability compared to unmodified small interfering RNA. J Med Chem. 2005; 48:901–4. 10.1021/jm049167j. 15715458

[21] Manoharan M, Akinc A, Pandey RK et al. Unique gene-silencing and structural properties of 2'-fluoro-modified siRNAs. Angew Chem Int Ed. 2011; 50:2284–8. 10.1002/anie.201006519. PMC3516925 21351337

[22] Pallan PS, Greene EM, Jicman PA et al. Unexpected origins of the enhanced pairing affinity of 2'-fluoro-modified RNA. Nucleic Acids Res. 2011; 39:3482–95. 10.1093/nar/gkq1270. 21183463 PMC3082899

[23] Patra A, Paolillo M, Charisse K et al. 2'-Fluoro RNA shows increased Watson-Crick H-bonding strength and stacking relative to RNA: evidence from NMR and thermodynamic data. Angew Chem Int Ed. 2012; 51:11863–6. 10.1002/anie.201204946. PMC3757553 23055396

[24] Kenski DM, Cooper AJ, Li JJ et al. Analysis of acyclic nucleoside modifications in siRNAs finds sensitivity at position 1 that is restored by 5'-terminal phosphorylation both in vitro and in vivo. Nucleic Acids Res. 2010; 38:660–71. 19917641 10.1093/nar/gkp913 PMC2811019

[25] Laursen MB, Pakula MM, Gao S et al. Utilization of unlocked nucleic acid (UNA) to enhance siRNA performance in vitro and in vivo. Mol Biosyst. 2010; 6:862–70. 20567772 10.1039/b918869j

[26] Kamiya Y, Takai J, Ito H et al. Enhancement of stability and activity of siRNA by terminal substitution with serinol nucleic acid (SNA). ChemBioChem. 2014; 15:2549–55. 10.1002/cbic.201402369. 25233814

[27] Matsuda S, Bala S, Liao JY et al. Shorter is better: the α-(l)-Threofuranosyl nucleic acid modification improves stability, potency, safety, and Ago2 binding and mitigates off-target effects of small interfering RNAs. J Am Chem Soc. 2023; 145:19691–706. 10.1021/jacs.3c04744. 37638886

[28] Schlegel MK, Janas MM, Jiang Y et al. From bench to bedside: improving the clinical safety of GalNAc-siRNA conjugates using seed-pairing destabilization. Nucleic Acids Res. 2022; 50:6656–70. 10.1093/nar/gkac539. 35736224 PMC9262600

[29] Egli M, Schlegel MK, Manoharan M Acyclic (S)-glycol nucleic acid (S-GNA) modification of siRNAs improves the safety of RNAi therapeutics while maintaining potency. RNA. 2023; 29:402–14. 10.1261/rna.079526.122. 36725319 PMC10019370

[30] Nielsen P, Dreiøe LH, Wengel J Synthesis and evaluation of oligodeoxynucleotides containing acyclic nucleosides: introduction of three novel analogues and a summary. Bioorg Med Chem. 1995; 3:19–28. 10.1016/0968-0896(94)00143-Q. 8612043

[31] Langkjaer N, Pasternak A, Wengel J UNA (unlocked nucleic acid): a flexible RNA mimic that allows engineering of nucleic acid duplex stability. Bioorg Med Chem. 2009; 17:5420–5. 10.1016/j.bmc.2009.06.045. 19604699

[32] Bramsen JB, Laursen MB, Nielsen AF et al. A large-scale chemical modification screen identifies design rules to generate siRNAs with high activity, high stability and low toxicity. Nucleic Acids Res. 2009; 37:2867–81. 10.1093/nar/gkp106. 19282453 PMC2685080

[33] Bramsen JB, Pakula MM, Hansen TB et al. A screen of chemical modifications identifies position-specific modification by UNA to most potently reduce siRNA off-target effects. Nucleic Acids Res. 2010; 38:5761–73. 10.1093/nar/gkq341. 20453030 PMC2943616

[34] Campbell MA, Wengel J Locked vs. unlocked nucleic acids (LNA vs. UNA): contrasting structures work towards common therapeutic goals. Chem Soc Rev. 2011; 40:5680–9. 10.1039/c1cs15048k. 21556437

[35] Snead NM, Escamilla-Powers JR, Rossi JJ et al. 5' unlocked nucleic acid modification improves siRNA targeting. Mol Ther Nucleic Acids. 2013; 2:e103 10.1038/mtna.2013.36. 23820891 PMC3732871

[36] Flockerzi D, Silber G, Charubala R et al. Nucleoside, XXXVII. Synthese und Eigenschaften von 2′-O- und 3′-O-(tert-Butyldi-methylsilyl)-5′-O-(4-methoxytrityl)- sowie 2′,3′-Bis-O-(tert-butyl-dimethylsilyl)ribonucleosiden — Ausgangssubstanzen für Oligoribonucleotid-Synthesen. Liebigs Ann Chem. 1981; 1981:1568–85. 10.1002/jlac.198119810907.

[37] Haraguchi K, Takeda S, Tanaka H Ring opening of 4',5'-epoxynucleosides: a novel stereoselective entry to 4'-C-branched nucleosides. Org Lett. 2003; 5:1399–402. 10.1021/ol020259h. 12713283

[38] Sofia MJ, Du J Preparation of 2',4'-substituted nucleoside analogs for use treating viral infections. 2009; US20090318380A1.

[39] Barbeyron R, Wengel J, Vasseur JJ et al. Boronic acid-based autoligation of nucleic acids: influence of the nature of the 3′-end ribonucleotidic strand. Monatsh. Chem. 2013; 144:495–500. 10.1007/s00706-012-0881-7.

[40] Lerner LM Adenine nucleosides derived from 6-deoxyhexofuranoses. J Org Chem. 1976; 41:306–10. 10.1021/jo00864a026. 1245905

[41] Hoye TR, Jeffrey CS, Shao F Mosher ester analysis for the determination of absolute configuration of stereogenic (chiral) carbinol carbons. Nat Protoc. 2007; 2:2451–8. 10.1038/nprot.2007.354. 17947986

[42] Vaish N, Chen F, Seth S et al. Improved specificity of gene silencing by siRNAs containing unlocked nucleobase analogs. Nucleic Acids Res. 2011; 39:1823–32. 10.1093/nar/gkq961. 21047800 PMC3061082

[43] Malek-Adamian E, Guenther DC, Matsuda S et al. 4′-C-Methoxy-2′-deoxy-2′-fluoro modified ribonucleotides improve metabolic stability and elicit efficient RNAi-mediated gene silencing. J Am Chem Soc. 2017; 139:14542–55. 10.1021/jacs.7b07582. 28937776

[44] Elkayam E, Kuhn CD, Tocilj A et al. The structure of human argonaute-2 in complex with miR-20a. Cell. 2012; 150:100–10. 10.1016/j.cell.2012.05.017. 22682761 PMC3464090

[45] Pettersen EF, Goddard TD, Huang CC et al. UCSF Chimera–a visualization system for exploratory research and analysis. J Comput Chem. 2004; 25:1605–12. 10.1002/jcc.20084. 15264254

[46] Case DA, Cheatham TE 3rd, Darden T et al. The Amber biomolecular simulation programs. J Comput Chem. 2005; 26:1668–88. 10.1002/jcc.20290. 16200636 PMC1989667

[47] Jinek M, Coyle SM, Doudna JA Coupled 5' nucleotide recognition and processivity in Xrn1-mediated mRNA decay. Mol Cell. 2011; 41:600–8. 10.1016/j.molcel.2011.02.004. 21362555 PMC3090138

