# Supplementary material for: Modified unlocked nucleic acid (MUNA) mitigates off-target effects of small interfering RNAs
Source: Nucleic Acids Res. 2025 Sep 29;53(18):gkaf937. doi: 10.1093/nar/gkaf937 (PMC12477598; doi:10.1093/nar/gkaf937)
Supplement: gkaf937_Supplemental_File [file gkaf937_supplemental_file.docx]

**Modified Unlocked Nucleic Acid (MUNA) Mitigates Off-target Effects of Small Interfering RNAs**

Shohei Mori^1^, Dhrubajyoti Datta^1^, Lydia Perkins^1^, Michelle Jung^1^, Lauren Blair Woods^1^, Alex Eaton^1^, June Qin^1^, Tim Racie^1^, MaryBeth Kim^1^, Dale C. Guenther^1^, Adam Castoreno^1^, Mark K. Schlegel^1^, Klaus Charisse^1^, Martin Egli^2^, Shigeo Matsuda^1,*^, and Muthiah Manoharan^1,*^

^1^*Alnylam Pharmaceuticals, 675 West Kendall, Cambridge, MA 02142, USA*

^2^*Department of Biochemistry and Center for Structural Biology, Vanderbilt University, School of Medicine, Nashville, TN 37232-0146, USA*

*Correspondence to: Email address mmanoharan@alnylam.com; shigeomatsuda@gmail.com

**SUPPORTING INFORMATION**

**Table of Contents**

Oligonucleotide characterizationS2

Table S1. MS (*m/z*) analysis of modified ONsS2

Modeling studiesS5

NMR characterization of new compounds………………………………………………………………..S6

**Oligonucleotide characterization**

**Table S1**. MS (*m/z*) analysis of modified oligonucleotides *^a^*

| ID | Sequence (5′-3′) | Mass (m/z) in negative mode (M−H)^−^ | |
| --- | --- | --- | --- |
|  |  | Calc. | Obs. |
| 1 | UACAGUCUAUGU | 3756.3 | 3755.5 |
| 2 | ACAUAGACUGUA | 3802.3 | 3801.8 |
| 3 | d(ACATAGACTGTA) | 3652.4 | 3652.3 |
| 4 | UACAG***U***CUAUGU | 3758.3 | 3757.5 |
| 5 | UACAG***U_5′S_***CUAUGU | 3772.3 | 3771.5 |
| 6 | UACAG***U_5′R_***CUAUGU | 3772.3 | 3771.5 |
| 7 | UACAG***U_2′S_***CUAUGU | 3772.3 | 3771.5 |
| 8 | UACAG***U_2′R_***CUAUGU | 3772.3 | 3771.5 |
| 9 | UACAG***U_3′S_***CUAUGU | 3772.3 | 3771.5 |
| 10 | UACAG***U_3′R_***CUAUGU | 3772.3 | 3771.5 |
| 11 | UACAG ***U_4′β_*** CUAUGU | 3788.3 | 3787.5 |
| 12(***U***) | dT_19_***U*** | 6024.9 | 6023.0 |
| 12(***U_5′S_***) | dT_19_***U_5′S_*** | 6038.9 | 6037.0 |
| 12(***U_5′R_***) | dT_19_***U_5′R_*** | 6038.9 | 6037.0 |
| 12(***U_2′S_***) | dT_19_***U_2′S_*** | 6038.9 | 6037.0 |
| 12(***U_2′R_***) | dT_19_***U_2′R_*** | 6038.9 | 6037.0 |
| 12(***U_3′S_***) | dT_19_***U_3′S_*** | 6038.9 | 6037.0 |
| 12(***U_3′R_***) | dT_19_***U_3′R_*** | 6038.9 | 6037.0 |
| 12(***U_4′β_***) | dT_19_***U_4′β_*** | 6054.9 | 6053.0 |
| 13(***U***) | dT_18_***U***dT | 6024.9 | 6023.0 |
| 13(***U_5′S_***) | dT_18_***U_5′S_***dT | 6038.9 | 6037.0 |
| 13(***U_5′R_***) | dT_18_***U_5′R_***dT | 6038.9 | 6037.0 |
| 13(***U_2′S_***) | dT_18_***U_2′S_***dT | 6038.9 | 6037.0 |
| 13(***U_2′R_***) | dT_18_***U_2′R_***dT | 6038.9 | 6037.0 |
| 13(***U_3′S_***) | dT_18_***U_3′S_***dT | 6038.9 | 6037.0 |
| 13(***U_3′R_***) | dT_18_***U_3′R_***dT | 6038.9 | 6037.0 |
| 13(***U_4′β_***) | dT_18_***U_4′β_***dT | 6054.9 | 6053.0 |
| 14(***U***) | dT_18_***UU*** | 6028.9 | 6027.0 |
| 14(***U_5′S_***) | dT_18_***U_5′S_U_5′S_*** | 6056.9 | 6055.0 |
| 14(***U_5′R_***) | dT_18_***U_5′R_U_5′R_*** | 6056.9 | 6055.0 |
| 14(***U_2′S_***) | dT_18_***U_2′S_U_2′S_*** | 6056.9 | 6055.0 |
| 14(***U_2′R_***) | dT_18_***U_2′R_U_2′R_*** | 6056.9 | 6055.0 |
| 14(***U_3′S_***) | dT_18_***U_3′S_U_3′S_*** | 6056.9 | 6055.0 |
| 14(***U_3′R_***) | dT_18_***U_3′R_U_3′R_*** | 6056.9 | 6055.0 |
| 14(***U_4′β_***) | dT_18_***U_4′β_U_4′β_*** | 6088.9 | 6087.0 |
| 15(***U***) | dT_19_•***U*** | 6041.0 | 6038.9 |
| 15(***U_5′S_***) | dT_19_•***U_5′S_*** | 6055.0 | 6053.0 |
| 15(***U_5′R_***) | dT_19_•***U_5′R_*** | 6055.0 | 6053.0 |
| 15(***U_2′S_***) | dT_19_•***U_2′S_*** | 6055.0 | 6053.0 |
| 15(***U_2′R_***) | dT_19_•***U_2′R_*** | 6055.0 | 6053.0 |
| 15(***U_3′S_***) | dT_19_•***U_3′S_*** | 6055.0 | 6053.0 |
| 15(***U_3′R_***) | dT_19_•***U_3′R_*** | 6055.0 | 6053.0 |
| 15(***U_4′β_***) | dT_19_•***U_4′β_*** | 6071.0 | 6069.0 |
| 16(***U***) | dT_18_***U***•dT | 6041.0 | 6038.9 |
| 16(***U_5′S_***) | dT_18_***U_5′S_***•dT | 6055.0 | 6053.0 |
| 16(***U_5′R_***) | dT_18_***U_5′R_***•dT | 6055.0 | 6053.0 |
| 16(***U_2′S_***) | dT_18_***U_2′S_***•dT | 6055.0 | 6053.0 |
| 16(***U_2′R_***) | dT_18_***U_2′R_***•dT | 6055.0 | 6053.0 |
| 16(***U_3′S_***) | dT_18_***U_3′S_***•dT | 6055.0 | 6053.0 |
| 16(***U_3′R_***) | dT_18_***U_3′R_***•dT | 6055.0 | 6053.0 |
| 16(***U_4′β_***) | dT_18_***U_4′β_***•dT | 6071.0 | 6069.0 |
| 17(***U***) | dT_18_***U***•***U*** | 6044.9 | 6043.0 |
| 17(***U_5′S_***) | dT_18_***U_5′S_***•***U_5′S_*** | 6073.0 | 6071.0 |
| 17(***U_5′R_***) | dT_18_***U_5′R_***•***U_5′R_*** | 6073.0 | 6071.0 |
| 17(***U_2′S_***) | dT_18_***U_2′S_***•***U_2′S_*** | 6073.0 | 6071.0 |
| 17(***U_2′R_***) | dT_18_***U_2′R_***•***U_2′R_*** | 6073.0 | 6071.0 |
| 17(***U_3′S_***) | dT_18_***U_3′S_***•***U_3′S_*** | 6073.0 | 6071.0 |
| 17(***U_3′R_***) | dT_18_***U_3′R_***•***U_3′R_*** | 6073.0 | 6071.0 |
| 17(***U_4′β_***) | dT_18_***U_4′β_***•***U_4′β_*** | 6105.0 | 6103.0 |
| 18(***U***) | ***U***dT_19_ | 6024.9 | 6023.0 |
| 18(***U_5′S_***) | ***U_5′S_***dT_19_ | 6038.9 | 6037.0 |
| 18(***U_5′R_***) | ***U_5′R_***dT_19_ | 6038.9 | 6037.0 |
| 18(***U_2′S_***) | ***U_2′S_***dT_19_ | 6038.9 | 6037.0 |
| 18(***U_2′R_***) | ***U_2′R_***dT_19_ | 6038.9 | 6037.0 |
| 18(***U_3′S_***) | ***U_3′S_***dT_19_ | 6038.9 | 6037.0 |
| 18(***U_3′R_***) | ***U_3′R_***dT_19_ | 6038.9 | 6037.0 |
| 18(***U_4′β_***) | ***U_4′β_***dT_19_ | 6054.9 | 6053.0 |
| 19(***U***) | ***U***•dT_19_ | 6041.0 | 6038.9 |
| 19(***U_5′S_***) | ***U_5′S_***•dT_19_ | 6055.0 | 6052.9 |
| 19(***U_5′R_***) | ***U_5′R_***•dT_19_ | 6055.0 | 6052.9 |
| 19(***U_2′S_***) | ***U_2′S_***•dT_19_ | 6055.0 | 6052.9 |
| 19(***U_2′R_***) | ***U_2′R_***•dT_19_ | 6055.0 | 6052.9 |
| 19(***U_3′S_***) | ***U_3′S_***•dT_19_ | 6055.0 | 6052.9 |
| 19(***U_3′R_***) | ***U_3′R_***•dT_19_ | 6055.0 | 6052.9 |
| 19(***U_4′β_***) | ***U_4′β_***•dT_19_ | 6071.0 | 6068.9 |
| 20 | u•u•cuugCfuCfUfAfuaaaccgugu* | 8638.4 | 8638.2 |
| 21 | a•Cf•acgguuuauagAfgCfaagaa•c•a | 7751.3 | 7751.6 |
| 22 | a•Cf•acgg***U***uuauagAfgCfaagaa•c•a | 7739.3 | 7736.3 |
| 23 | a•Cf•acgg***U_5′S_***uuauagAfgCfaagaa•c•a | 7753.3 | 7750.3 |
| 24 | a•Cf•acgg***U_5′R_***uuauagAfgCfaagaa•c•a | 7753.3 | 7750.3 |
| 25 | a•Cf•acgg***U_2′S_***uuauagAfgCfaagaa•c•a | 7753.3 | 7750.3 |
| 26 | a•Cf•acgg***U_2′R_***uuauagAfgCfaagaa•c•a | 7753.3 | 7750.3 |
| 27 | a•Cf•acgg***U_3′S_***uuauagAfgCfaagaa•c•a | 7753.3 | 7750.3 |
| 28 | a•Cf•acgg***U_3′R_***uuauagAfgCfaagaa•c•a | 7753.3 | 7750.3 |
| 29 | a•g•uguuCfuUfGfCfucuauaaaca* | 8685.5 | 8685.5 |
| 30 | u•Gf•uuuauagagcaAfgAfacacu•g•u | 7708.2 | 7707.5 |
| 31 | u•Gf•uuua***U***agagcaAfgAfacacu•g•u | 7694.2 | 7691.2 |
| 32 | u•Gf•uuua***U_5′S_***agagcaAfgAfacacu•g•u | 7708.2 | 7705.2 |
| 33 | u•Gf•uuua***U_5′R_***agagcaAfgAfacacu•g•u | 7708.2 | 7705.2 |
| 34 | u•Gf•uuua***U_2′S_***agagcaAfgAfacacu•g•u | 7708.2 | 7705.2 |
| 35 | u•Gf•uuua***U_2′R_***agagcaAfgAfacacu•g•u | 7708.2 | 7705.2 |
| 36 | u•Gf•uuua***U_3′S_***agagcaAfgAfacacu•g•u | 7708.2 | 7705.2 |
| 37 | u•Gf•uuua***U_3′R_***agagcaAfgAfacacu•g•u | 7708.2 | 7705.2 |
| 38 | u•Gf•uuua***U_4′β_***agagcaAfgAfacacu•g•u | 7724.2 | 7721.2 |

*^a^* Uppercase letters (B) = ribonucleotides, prefix d (dB) = deoxyribonucleotides, uppercase with lowercase f (Bf) = 2′-fluoro nucleotides, lowercase letters (b) = 2′-OMe nucleotides, colored uppercase italicized letters with and without subscript = modified and unmodified unlocked nucleotides, ● = phosphorothiate linkage, P = 5′-monophosphate, * = triantennary GalNAc ligand.

| * (GalNAc) = |  |
| --- | --- |

**Modeling study**

**Figure S1.** Two views of the computational model of Ago2 in complex with RNA modified with UNA. (A) Conformation and protein interactions of UNA-U at position 7 of the antisense strand. (B) The UNA-modified strand with overlaid miR-20a. UNA is shown with carbon atoms colored in cyan, hydrogen bonds are thin solid lines, and selected Ago2 side chains are labeled.

**NMR characterization of new compounds**


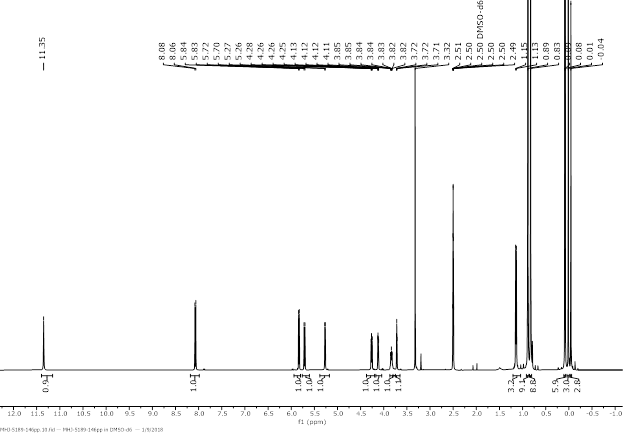


^1^H NMR (400 MHz, DMSO-*d*_6_) of **3S**


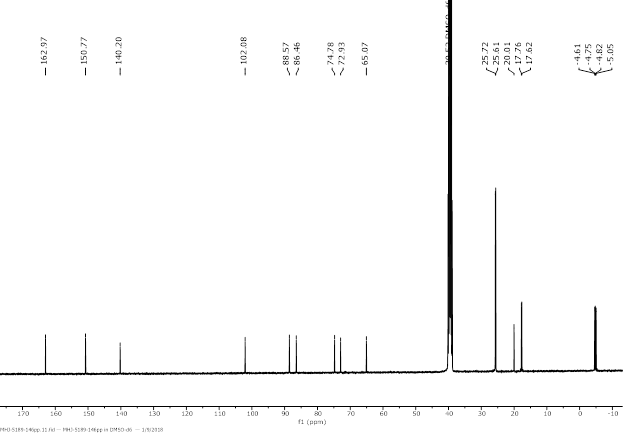


^13^C NMR (101 MHz, DMSO-*d*_6_) of **3S**


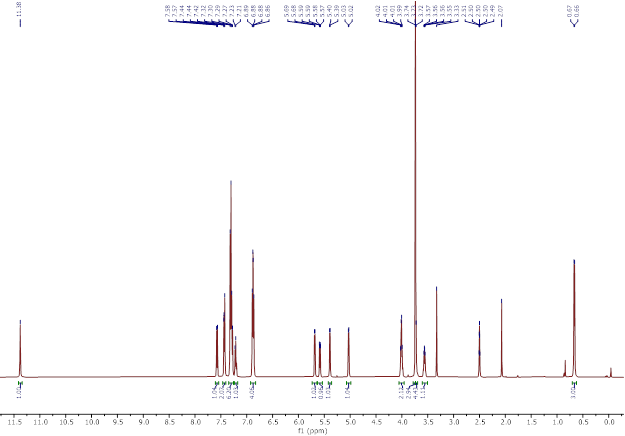


^1^H NMR (500 MHz, DMSO-*d*_6_) of **4S**


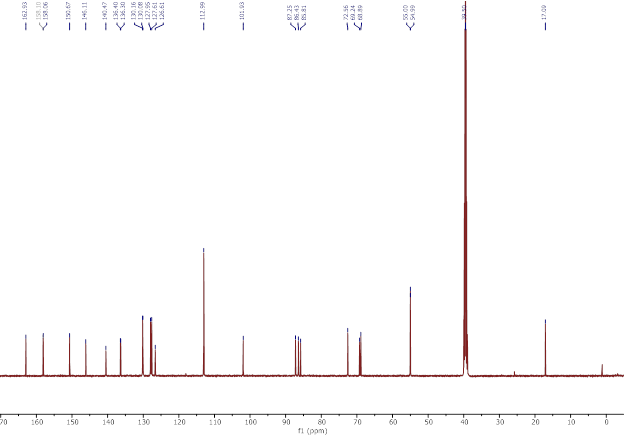


^13^C NMR (126 MHz, DMSO-*d*_6_) of **4S**


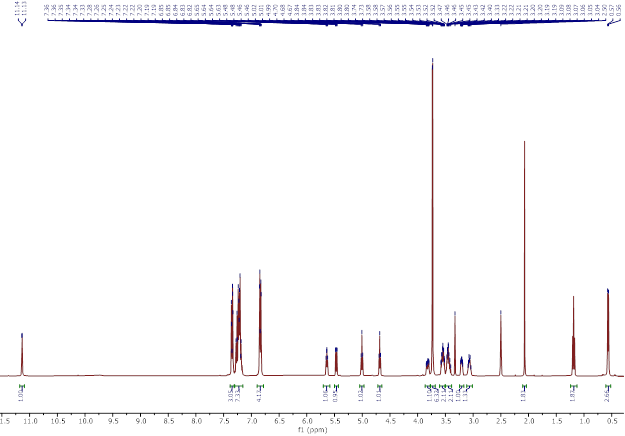


^1^H NMR (400 MHz, DMSO-*d*_6_) of **5S**


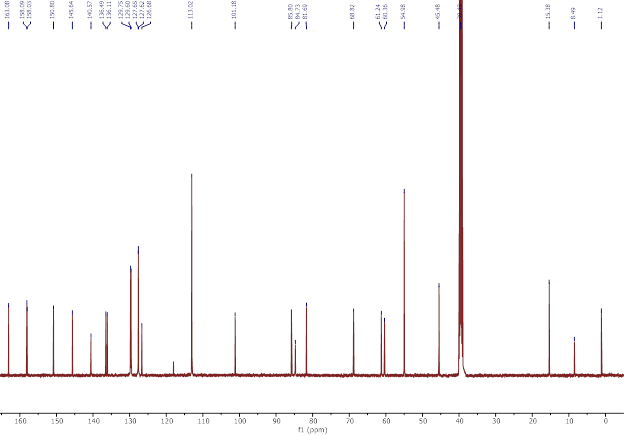


^13^C NMR (126 MHz, DMSO-*d*_6_) of **5S**


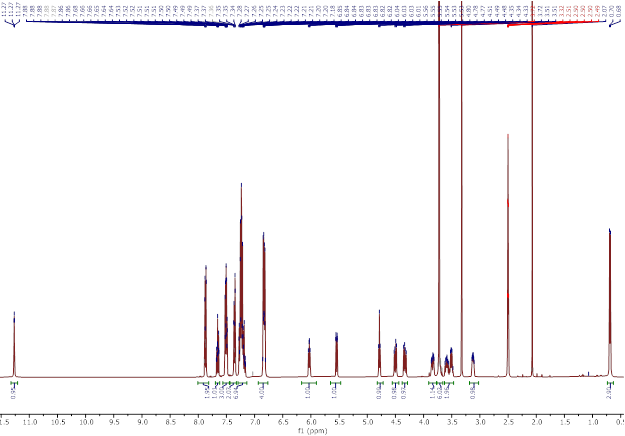


^1^H NMR (400 MHz, DMSO-*d*_6_) of **6S**


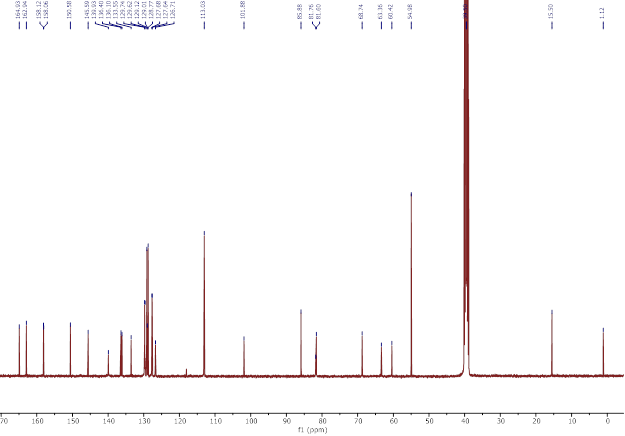


^13^C NMR (101 MHz, DMSO-*d*_6_) of **6S**


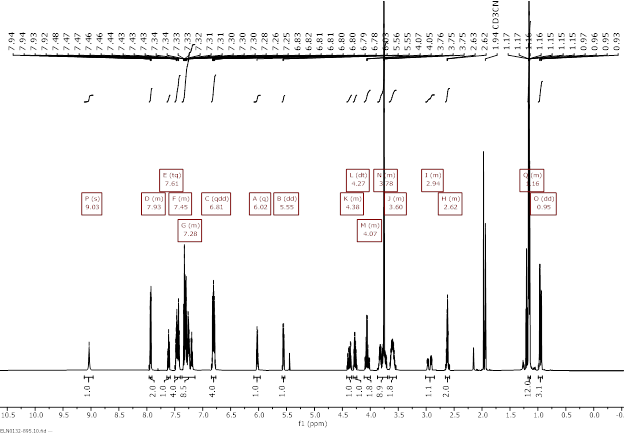


^1^H NMR (600 MHz, CD_3_CN) of **7S**


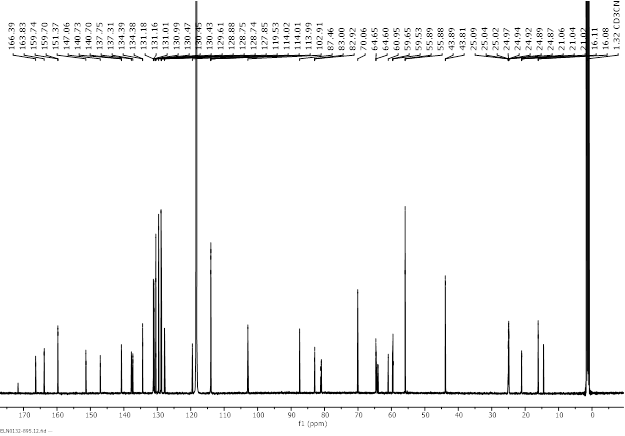


^13^C NMR (151 MHz, CD_3_CN) of **7S**


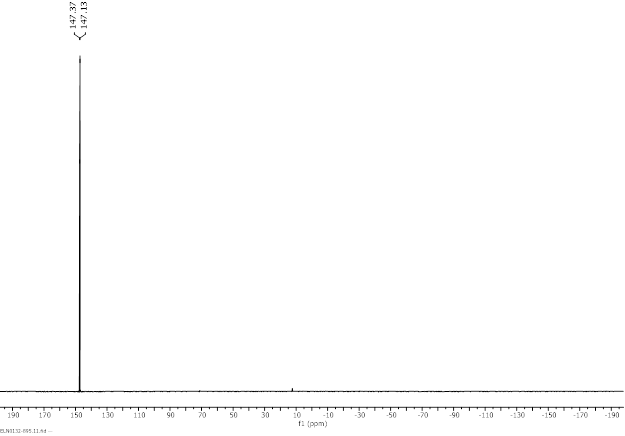


^31^P NMR (243 MHz, CD_3_CN) of **7S**


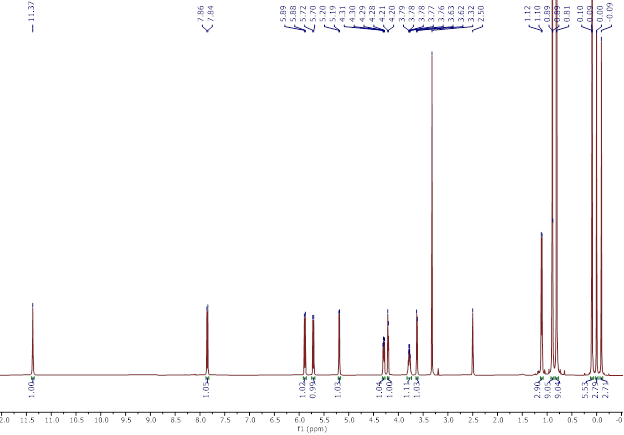


^1^H NMR (400 MHz, DMSO-*d*_6_) of **3R**


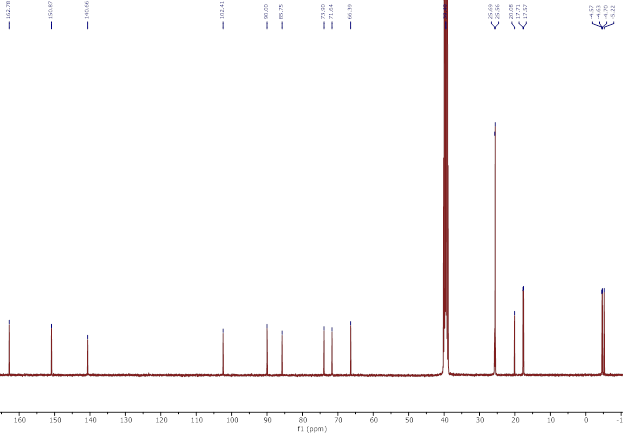


^13^C NMR (101 MHz, DMSO-*d*_6_) of **3R**


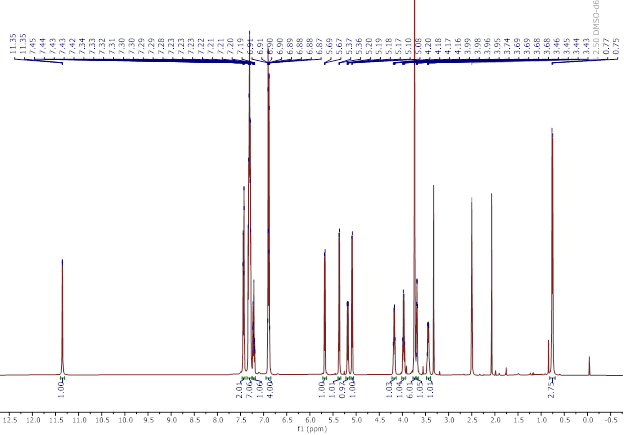


^1^H NMR (400 MHz, DMSO-*d*_6_) of **4R**


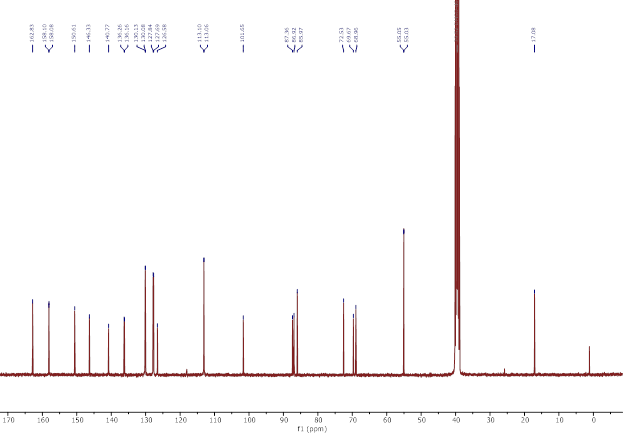


^13^C NMR (101 MHz, DMSO-*d*_6_) of **4R**


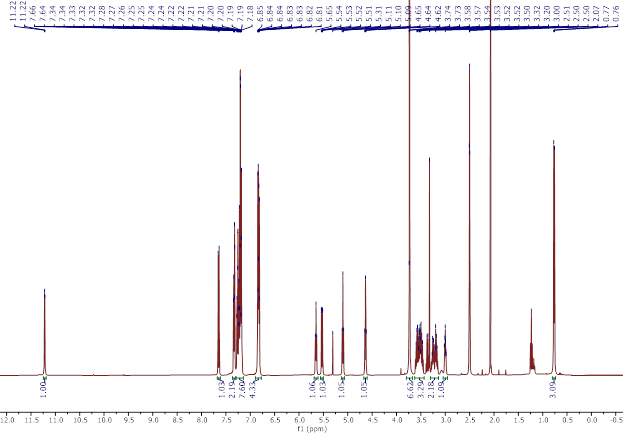


^1^H NMR (400 MHz, DMSO-*d*_6_) of **5R**


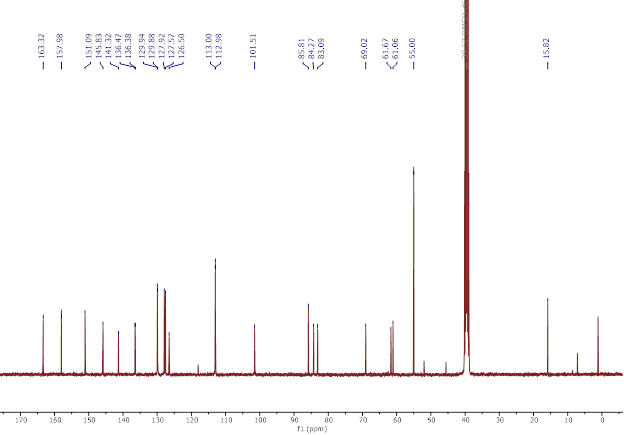


^13^C NMR (101 MHz, DMSO-*d*_6_) of **5R**


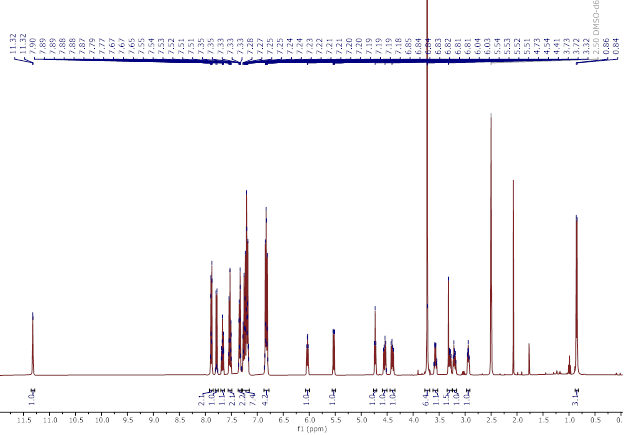


^1^H NMR (400 MHz, DMSO-*d*_6_) of **6R**


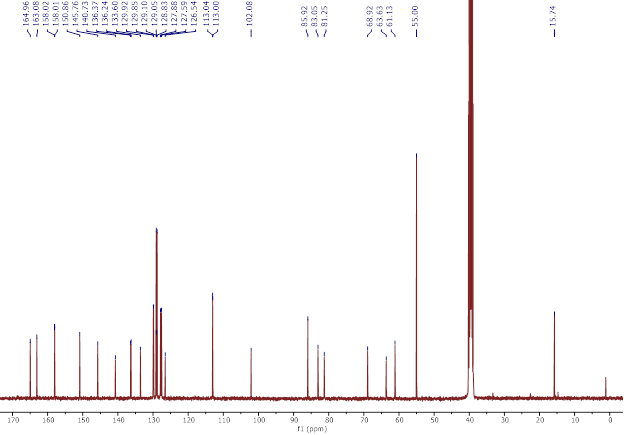


^13^C NMR (101 MHz, DMSO-*d*_6_) of **6R**


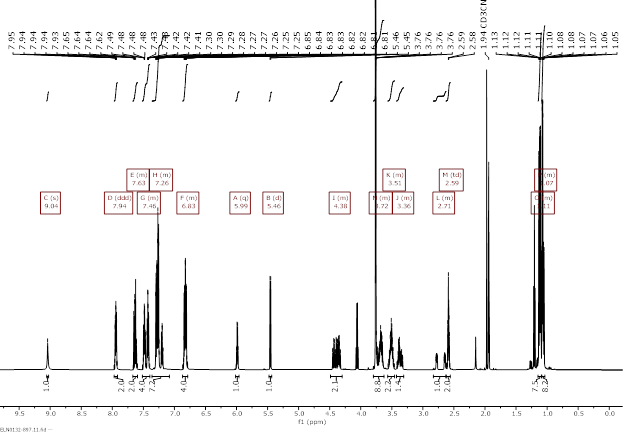


^1^H NMR (600 MHz, CD_3_CN) of **7R**


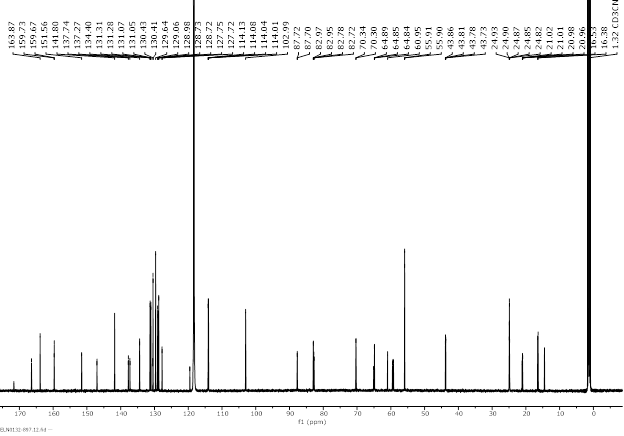


^13^C NMR (151 MHz, CD_3_CN) of **7R**


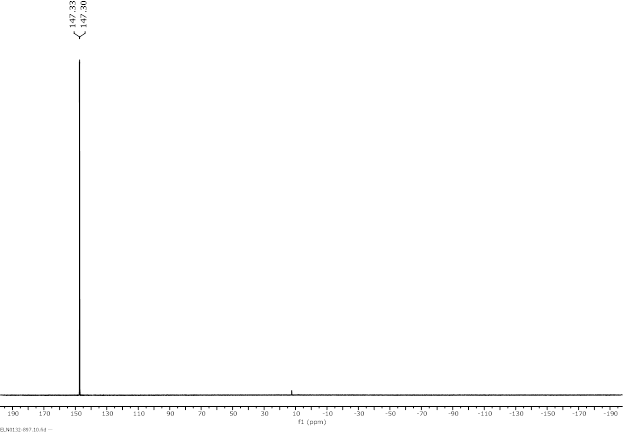


^31^P NMR (243 MHz, CD_3_CN) of **7R**


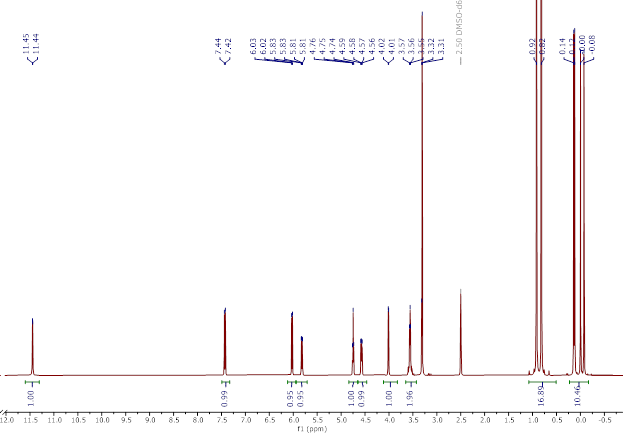


^1^H NMR (400 MHz, DMSO-*d*_6_) of **9**


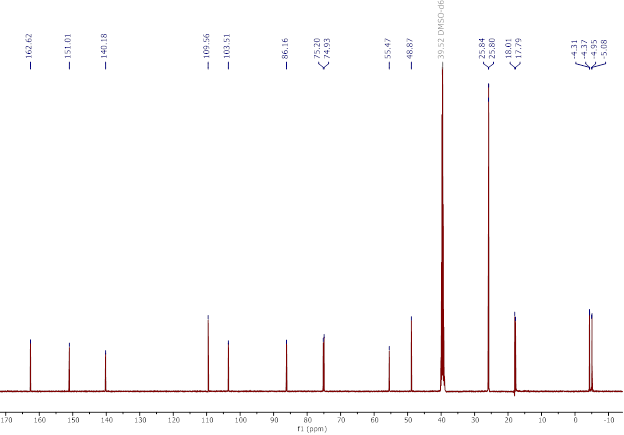


^13^C NMR (126 MHz, DMSO-*d*_6_) of **9**


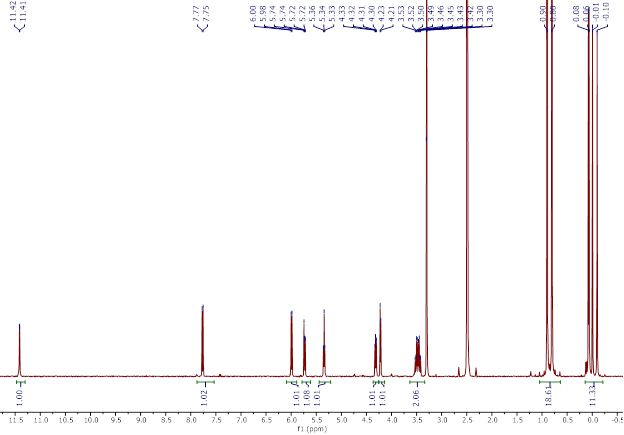


^1^H NMR (400 MHz, DMSO-*d*_6_) of **10**


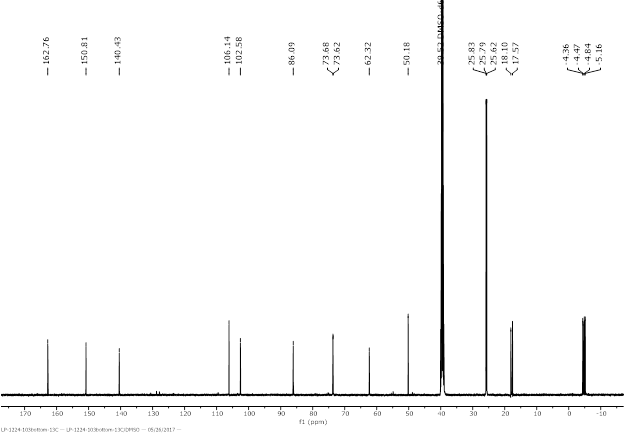


^13^C NMR (126 MHz, DMSO-*d*_6_) of **10**


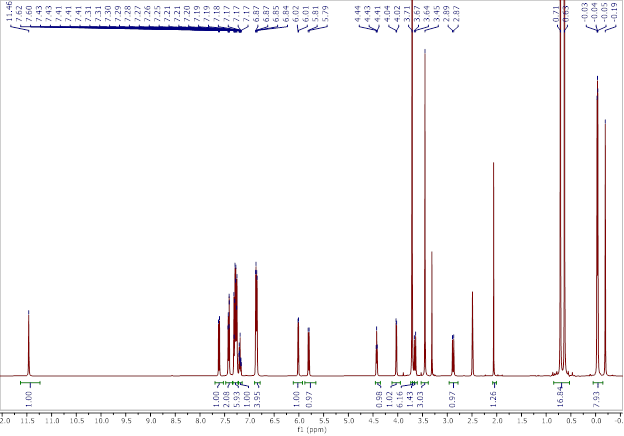


^1^H NMR (400 MHz, DMSO-*d*_6_) of **11** (step 1)


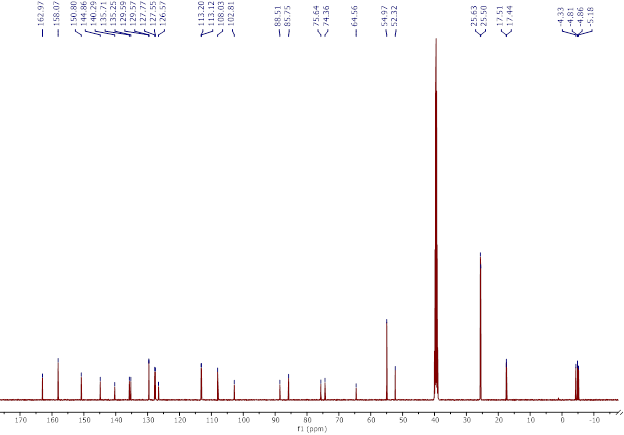


^13^C NMR (126 MHz, DMSO-*d*_6_) of **11** (step 1)


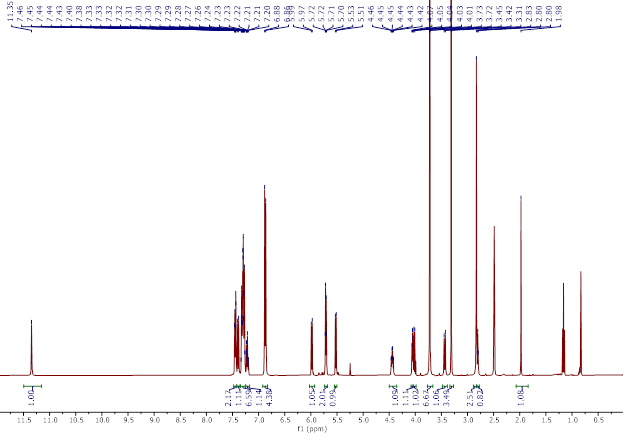


^1^H NMR (400 MHz, DMSO-*d*_6_) of **11** (step 2)


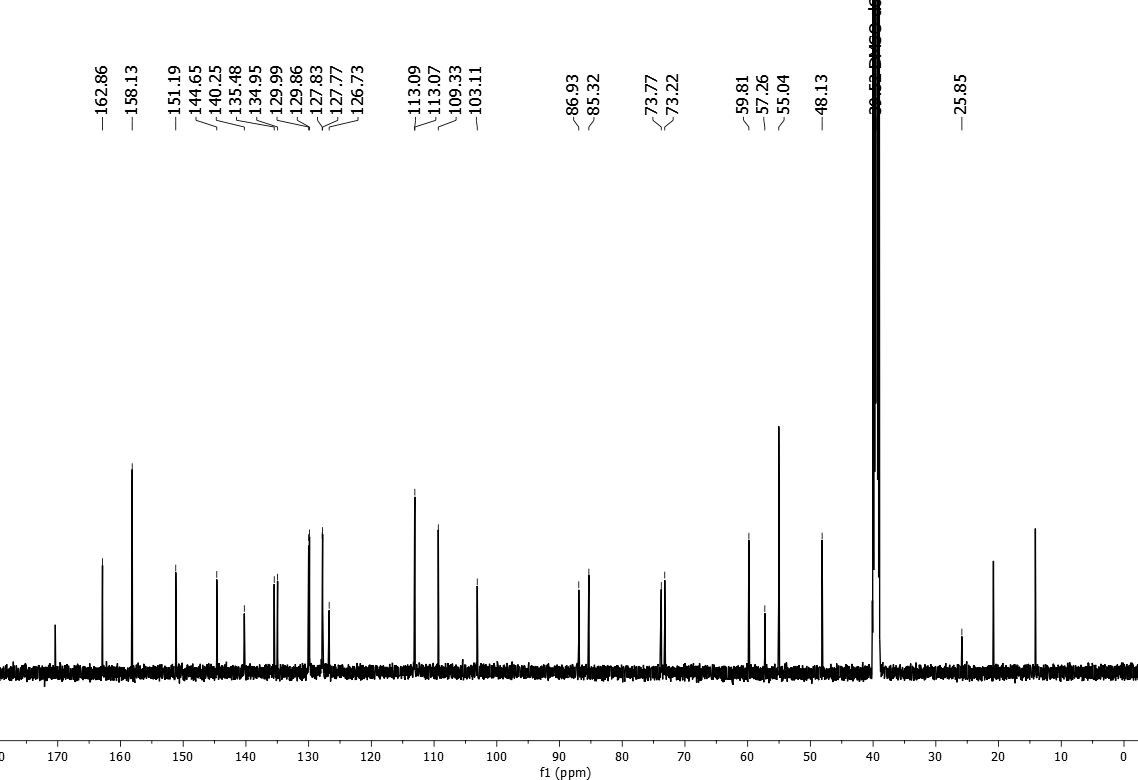


^13^C NMR (126 MHz, DMSO-*d*_6_) of **11** (step 2)


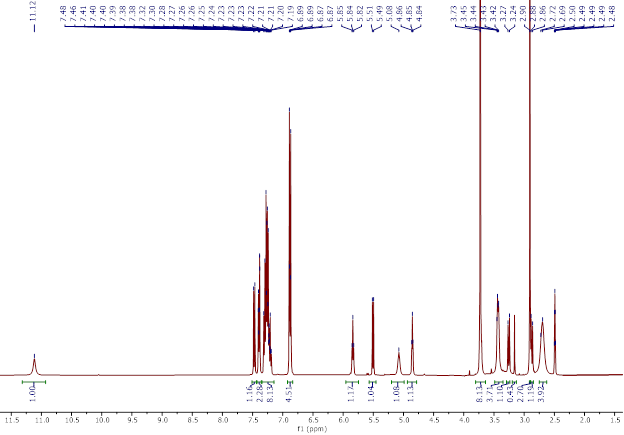


^1^H NMR (400 MHz, DMSO-*d*_6_) of **12**


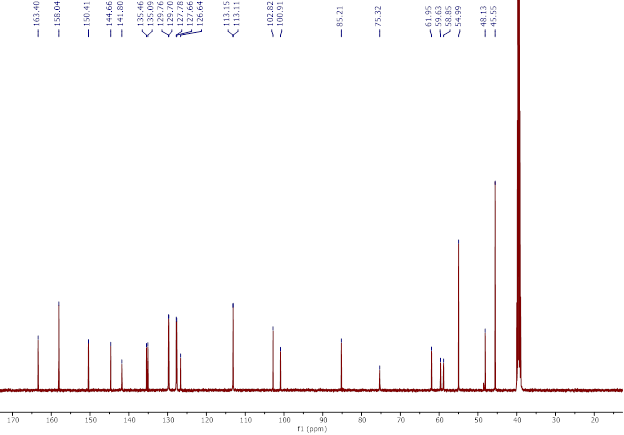


^13^C NMR (126 MHz, DMSO-*d*_6_) of **12**


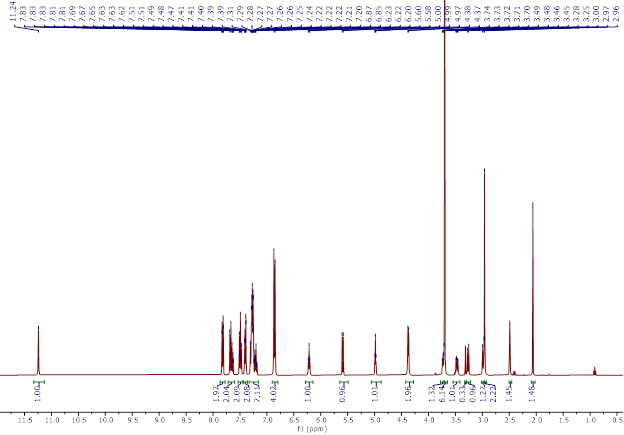


^1^H NMR (400 MHz, DMSO-*d*_6_) of **13**


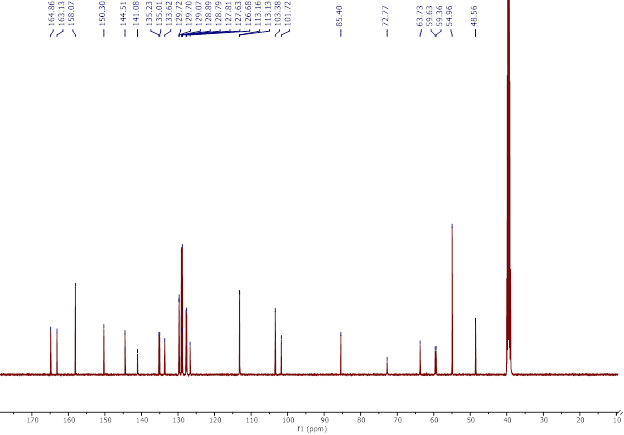


^13^C NMR (126 MHz, DMSO-*d*_6_) of **13**


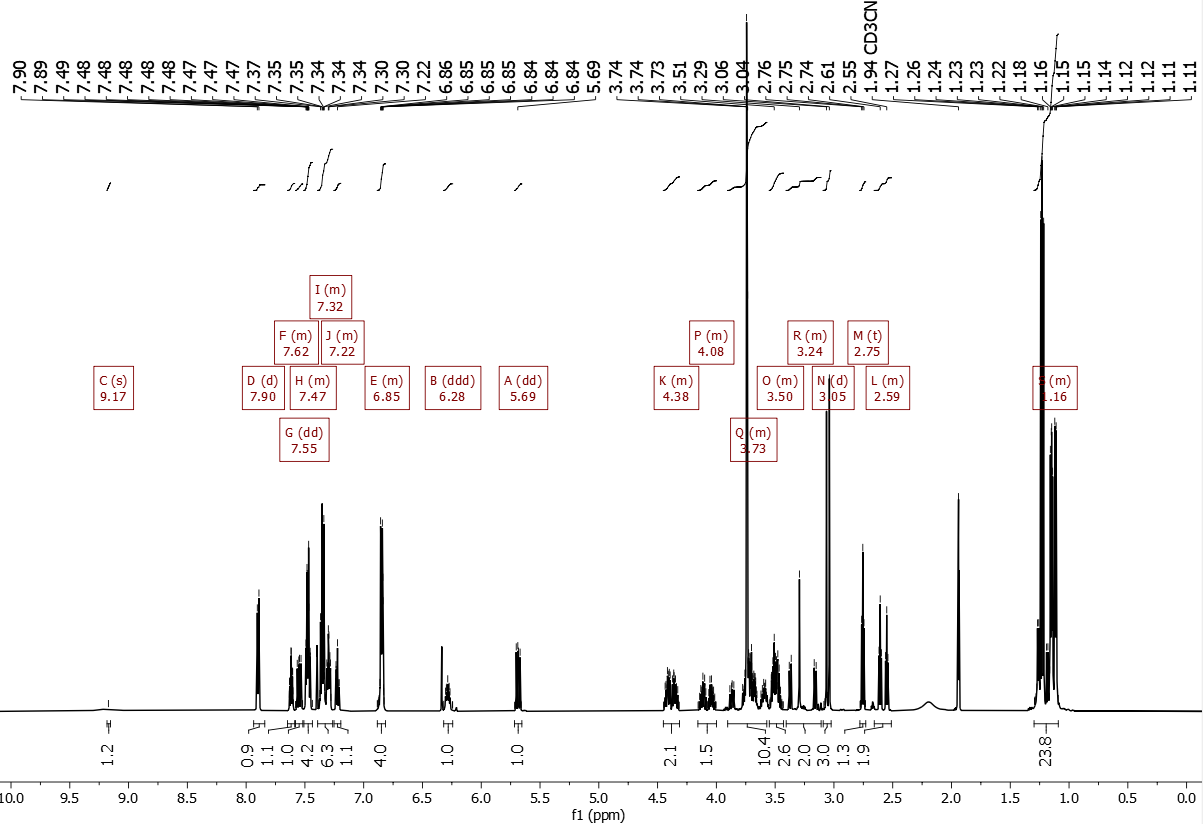


^1^H NMR (600 MHz, CD_3_CN) of **14**


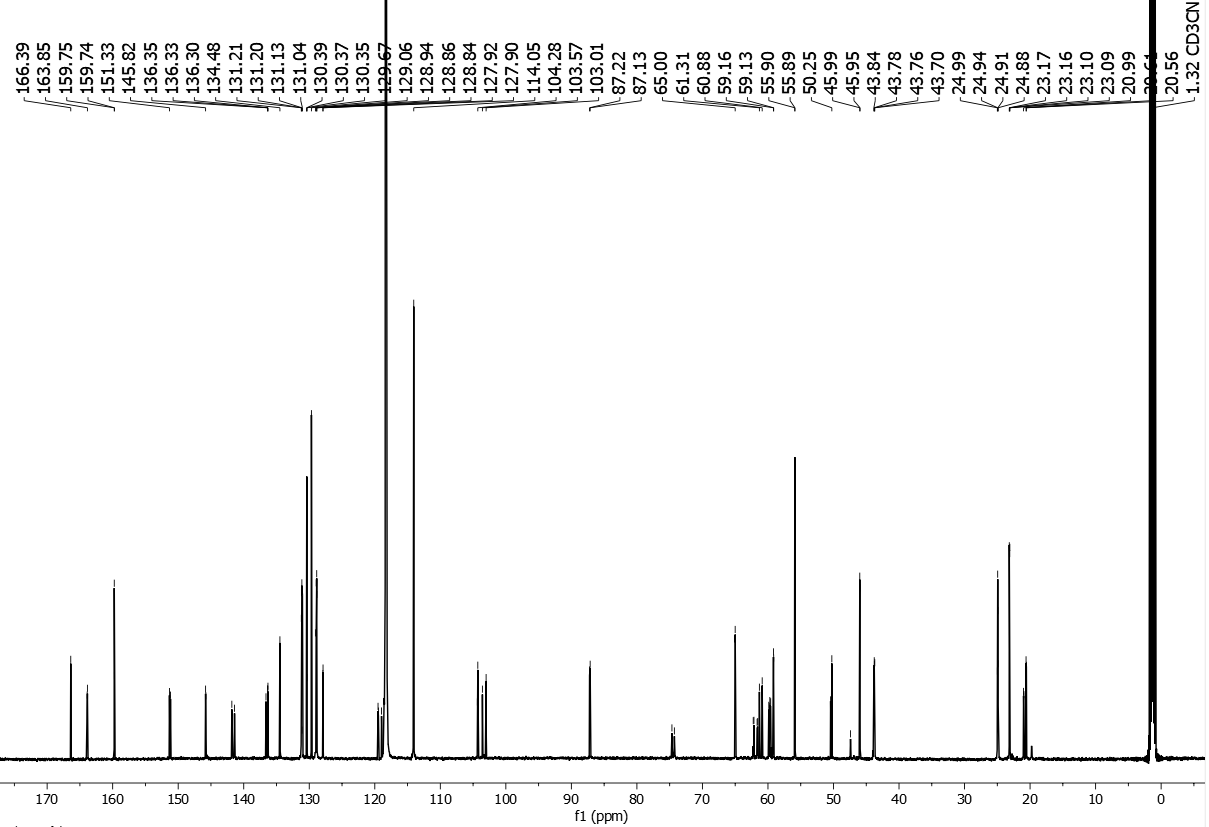


^13^C NMR (151 MHz, CD_3_CN) of **14**


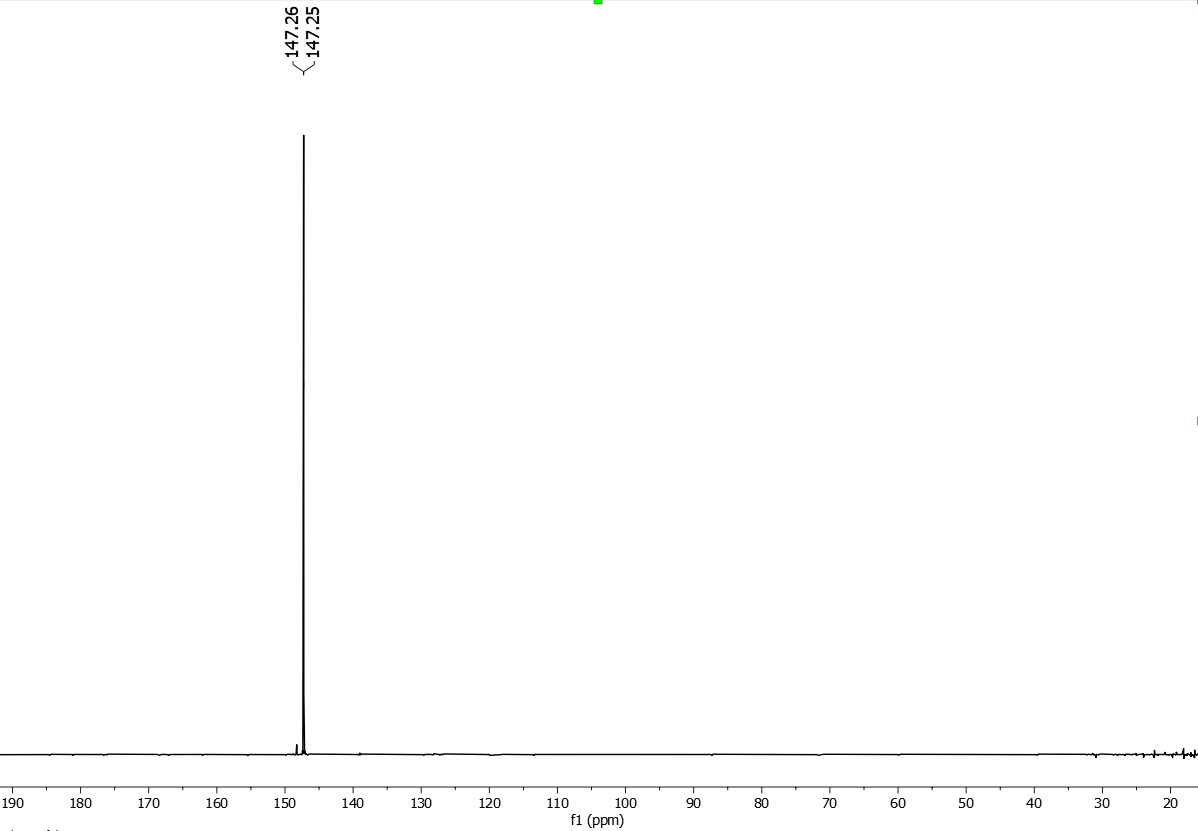


^31^P NMR (243 MHz, CD_3_CN) of **14**


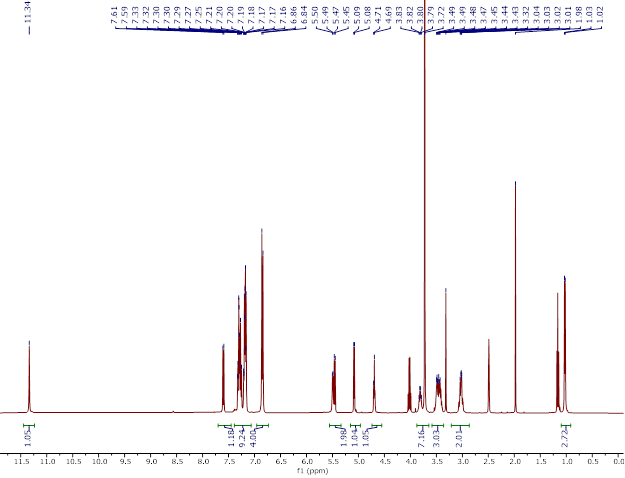


^1^H NMR (400 MHz, DMSO-*d*_6_) of **16**


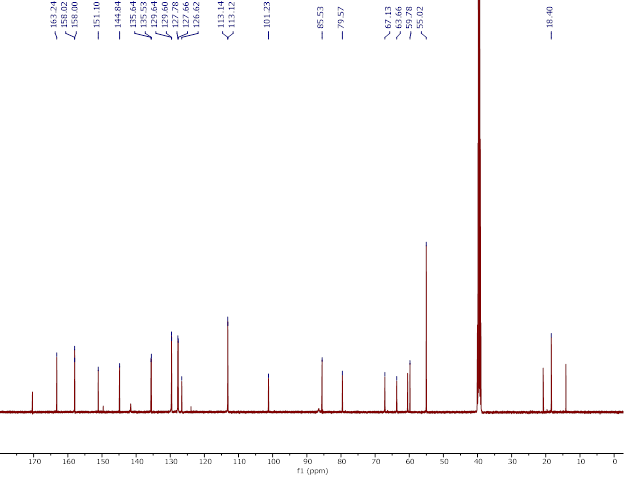


^13^C NMR (126 MHz, DMSO-*d*_6_) of **16**


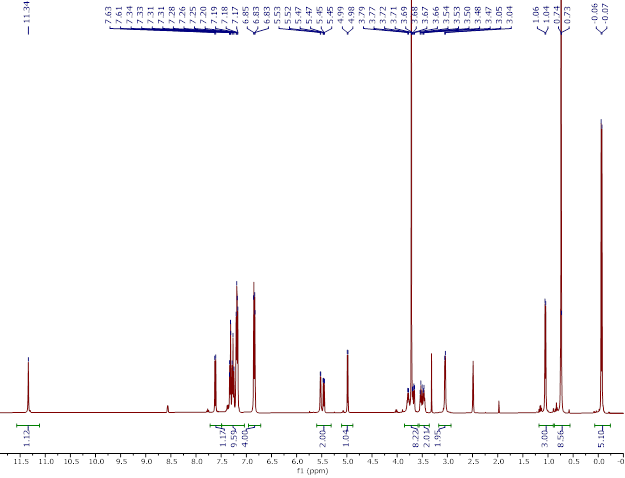


^1^H NMR (400 MHz, DMSO-*d*_6_) NMR of **17S**


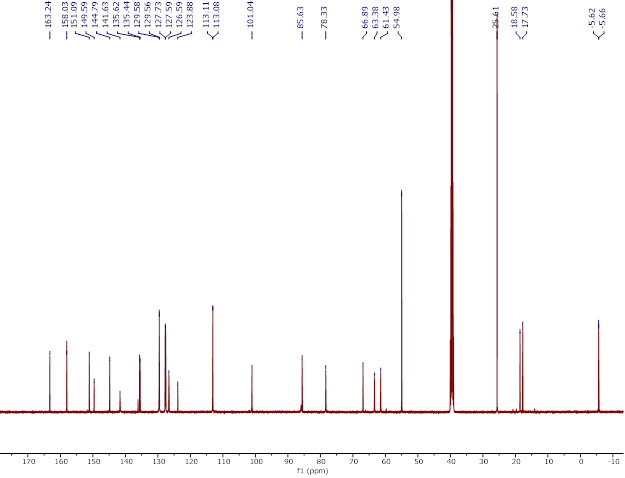


^13^C NMR (126 MHz, DMSO-*d*_6_) of **17S**


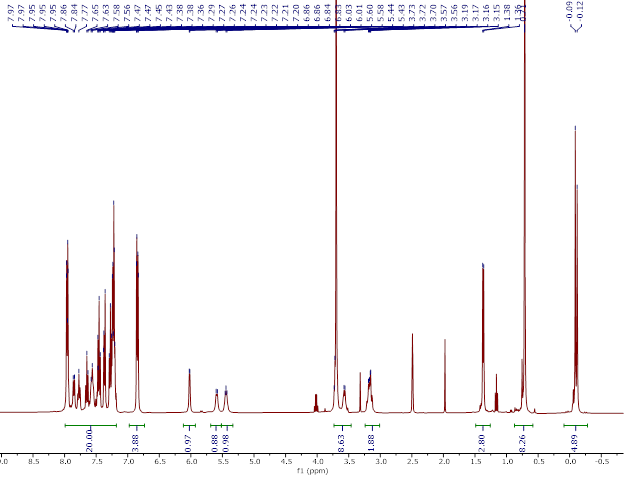


^1^H NMR (400 MHz, DMSO-*d*_6_) NMR of **18S**


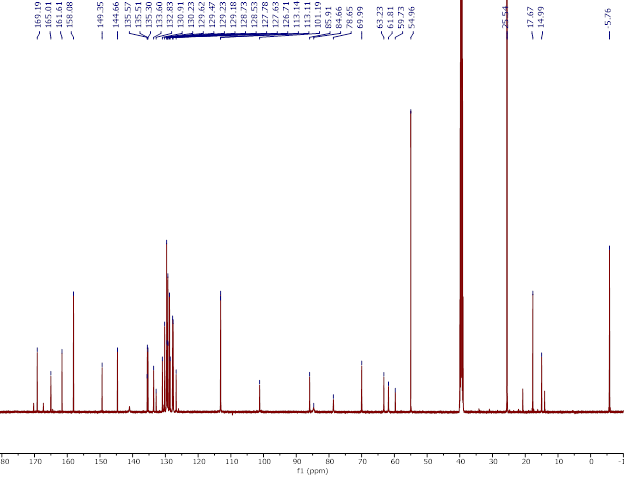


^13^C NMR (126 MHz, DMSO-*d*_6_) of **18S**


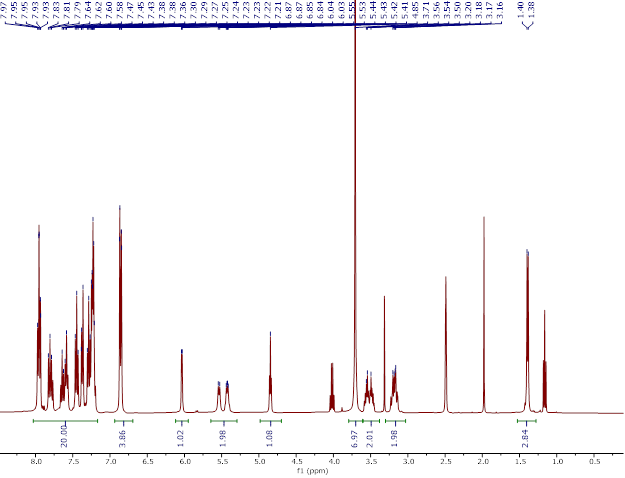


^1^H NMR (400 MHz, DMSO-*d*_6_) of **19S**


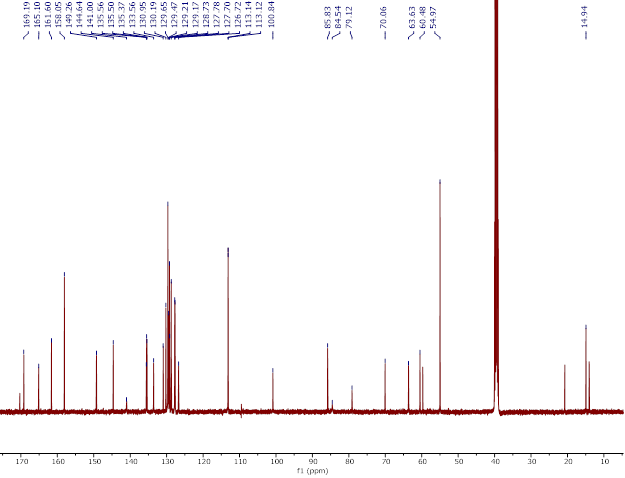


^13^C NMR (126 MHz, DMSO-*d*_6_) of **19S**


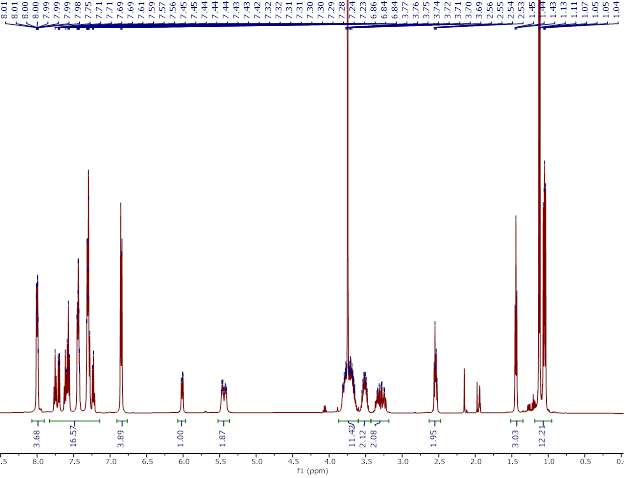


^1^H NMR (500 MHz, CD_3_CN) of **20S**


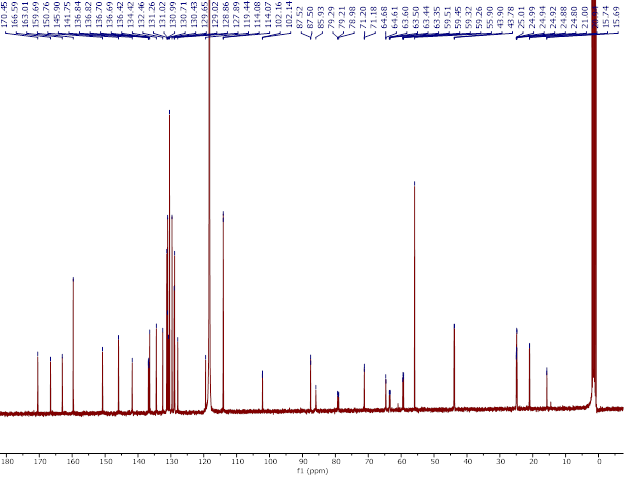


^13^C NMR (101 MHz, CD_3_CN) of **20S**


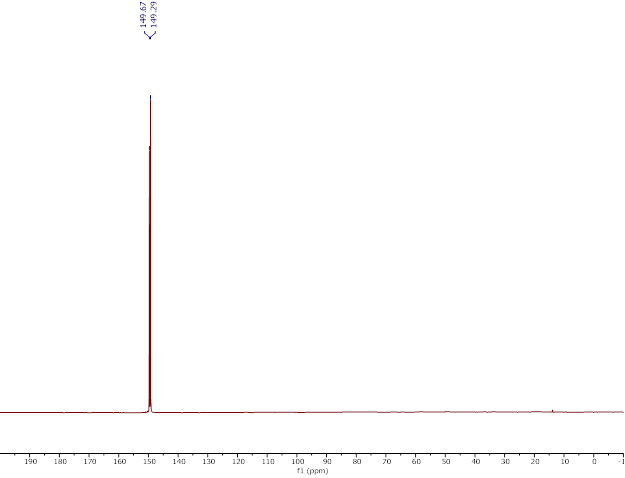


^31^P NMR (202 MHz, CD_3_CN) of **20S**


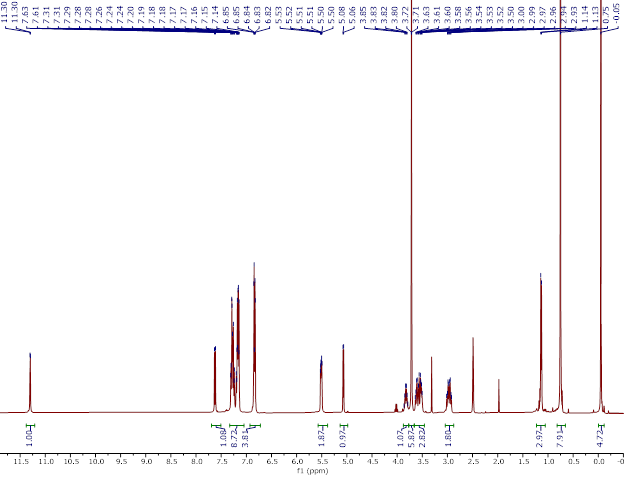


^1^H NMR (400 MHz, DMSO-*d*_6_) of **17R**


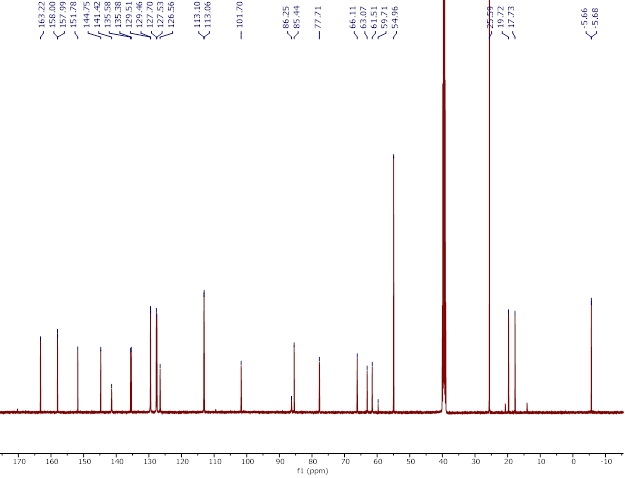


^13^C NMR (126 MHz, DMSO-*d*_6_) of **17R**


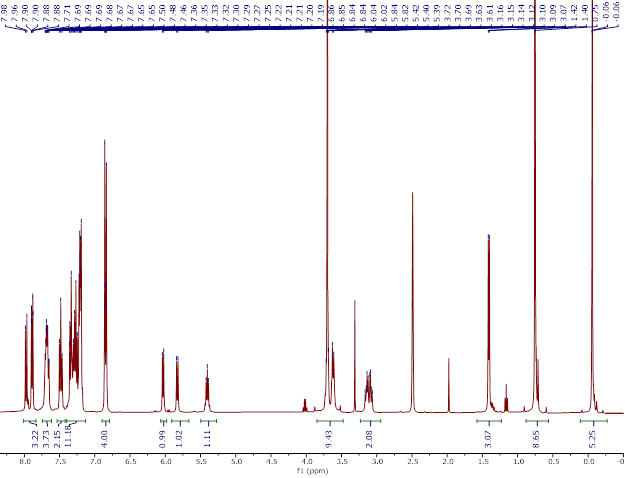


^1^H NMR (400 MHz, DMSO-*d*_6_) of **18R**


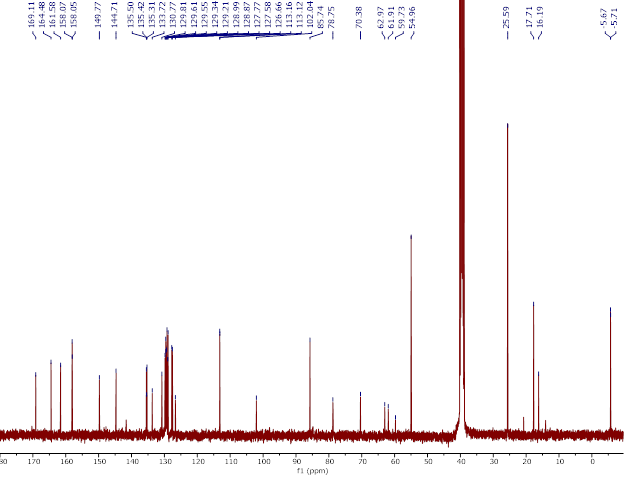


^13^C NMR (101 MHz, DMSO-*d*_6_) of **18R**


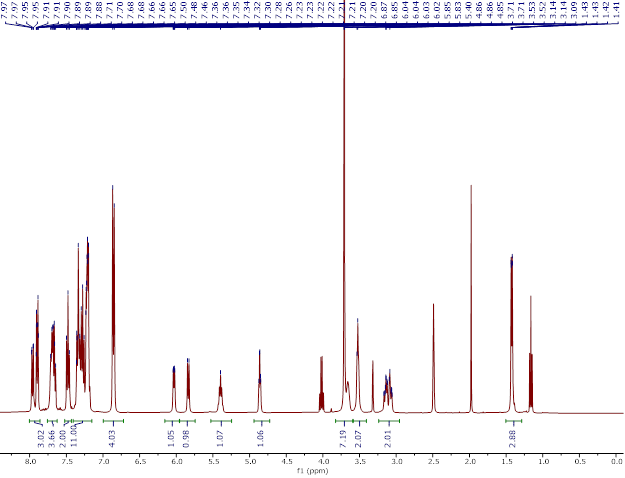


^1^H NMR (400 MHz, DMSO-*d*_6_) of **19R**


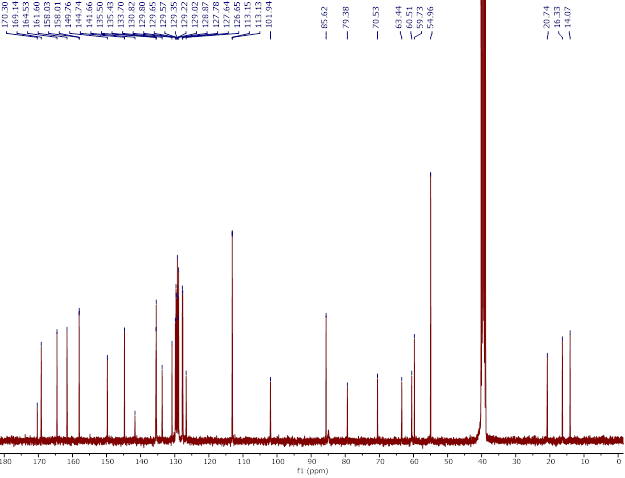


^13^C NMR (101 MHz, DMSO-*d*_6_) of **19R**


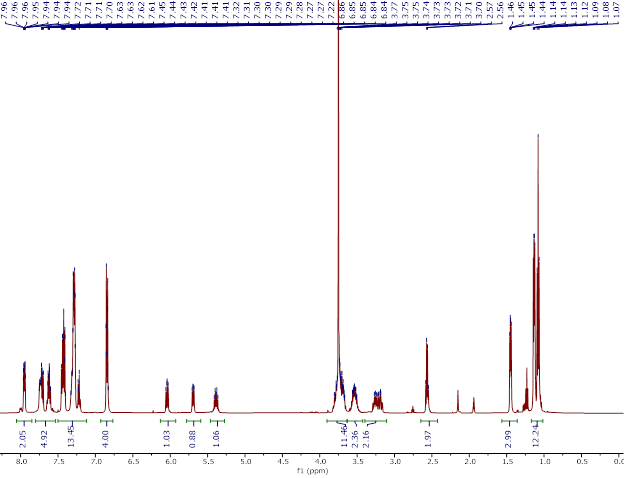


^1^H NMR (500 MHz, CD_3_CN) of **20R**


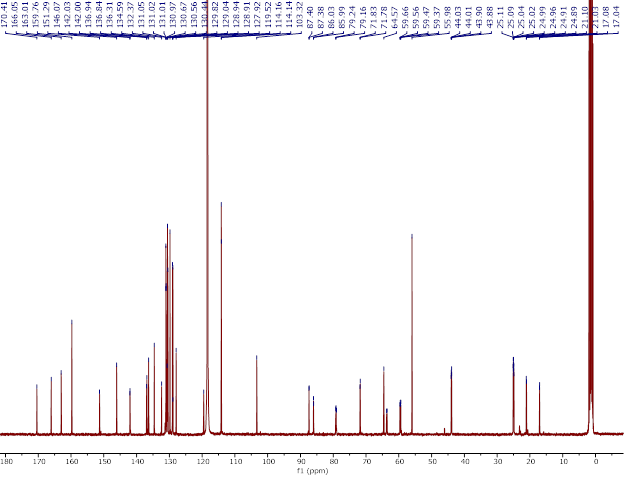


^13^C NMR (101 MHz, CD_3_CN) of **20R**


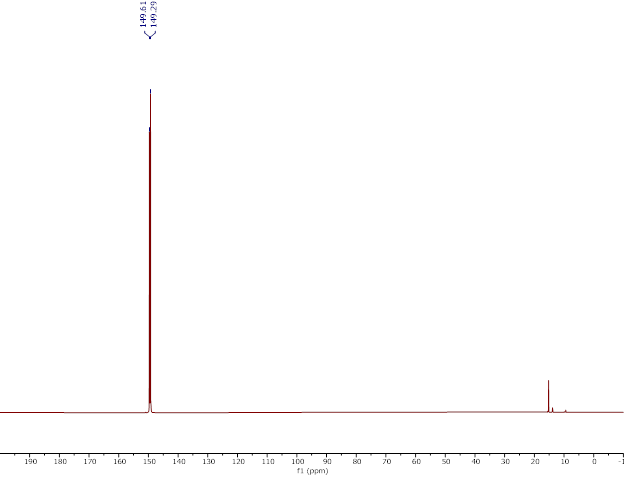


^31^P NMR (202 MHz, CD_3_CN) of **20R**


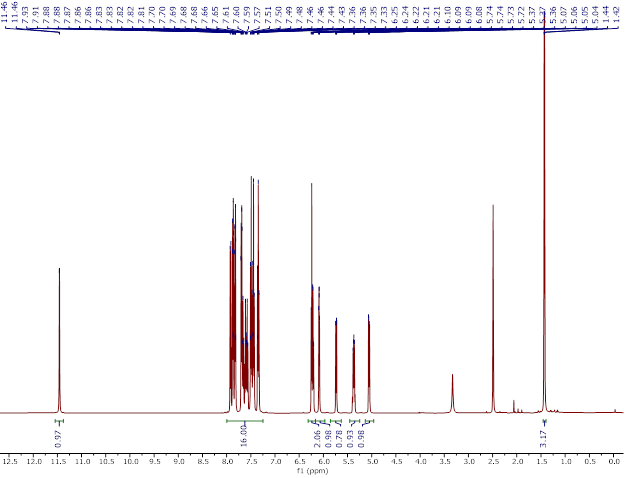


^1^H NMR (500 MHz, DMSO-*d*_6_) of **22**


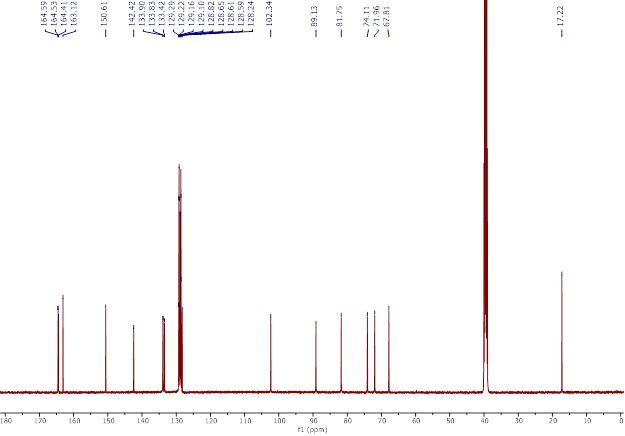


^13^C (126 MHz, DMSO-*d*_6_) of **22**


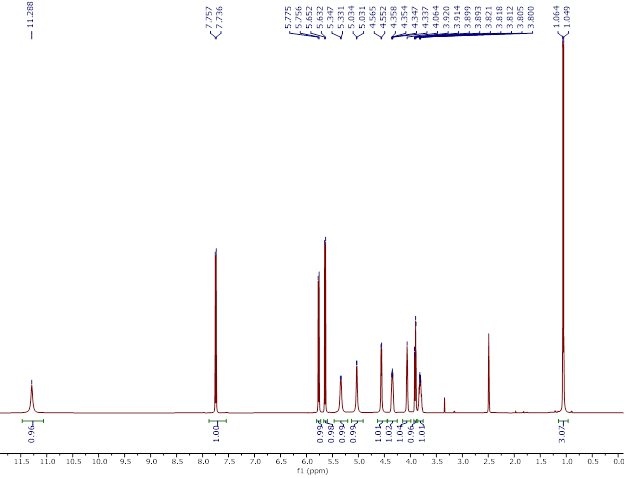


^1^H NMR (400 MHz, DMSO-*d*_6_) of **23**


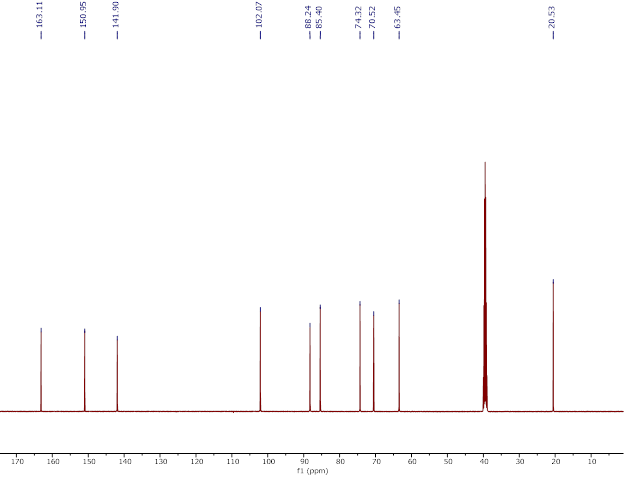


^13^C (126 MHz, DMSO-*d*_6_) of **23**


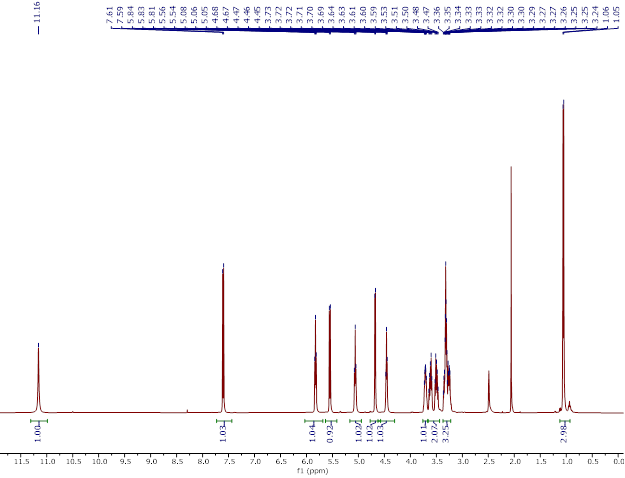


^1^H NMR (400 MHz, DMSO-*d*_6_) of **24**


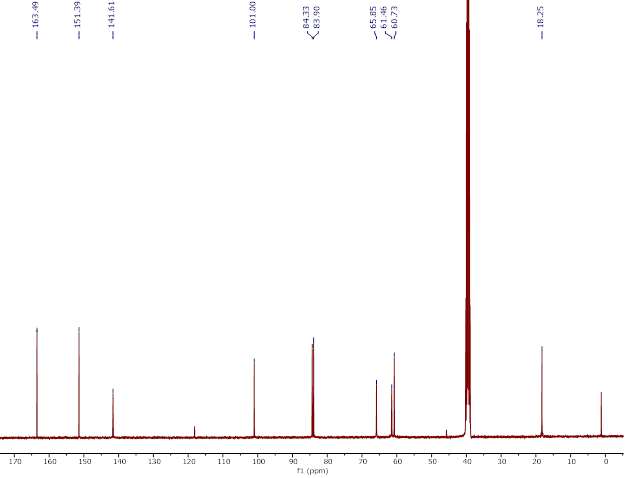


^13^C NMR (101 MHz, DMSO-*d*_6_) of **24**


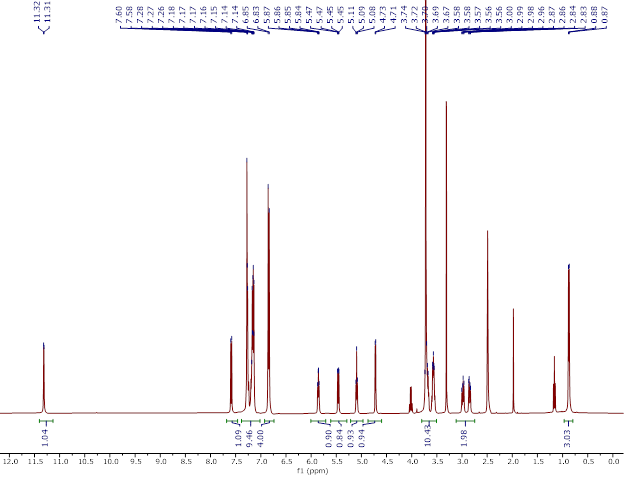


^1^H NMR (400 MHz, DMSO-*d*_6_) of **25S**


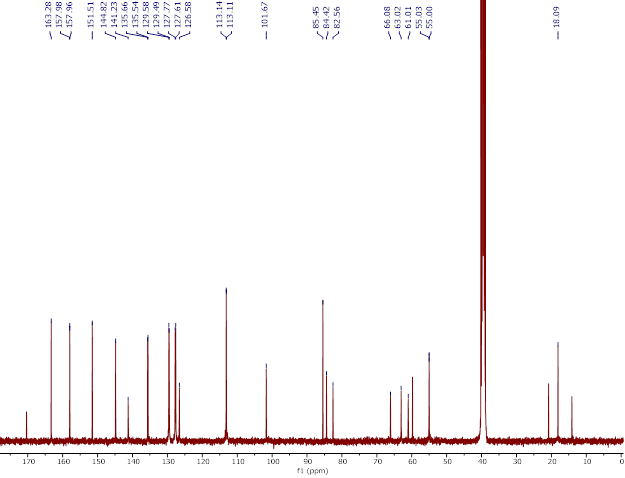


^13^C NMR (101 MHz, DMSO-*d*_6_) of **25S**


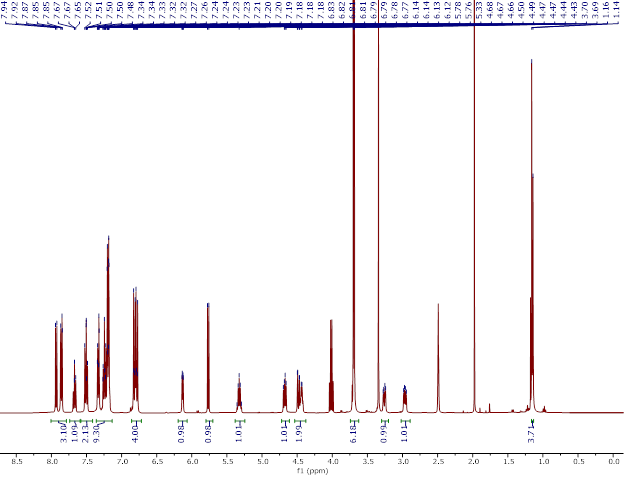


^1^H NMR (400 MHz, DMSO-*d*_6_) of **26**


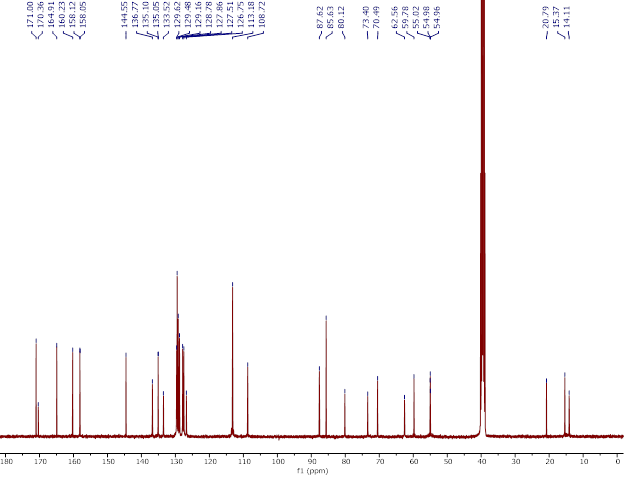


^13^C NMR (101 MHz, DMSO-*d_6_*) of **26**


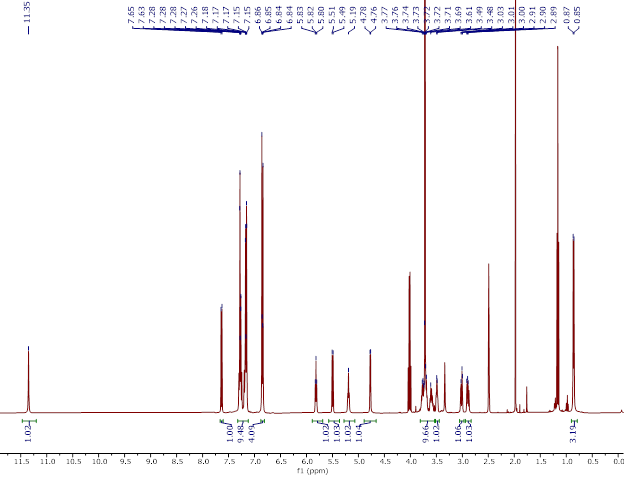


^1^H NMR (500 MHz, DMSO-*d*_6_) of **25R**


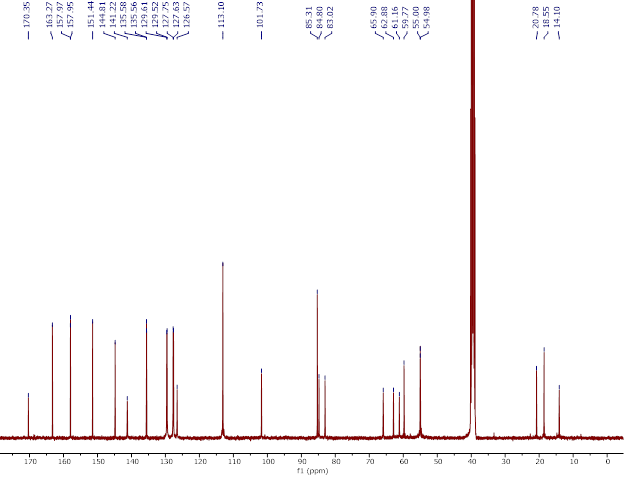


^13^C NMR (101 MHz, DMSO-*d*_6_) of **25R**


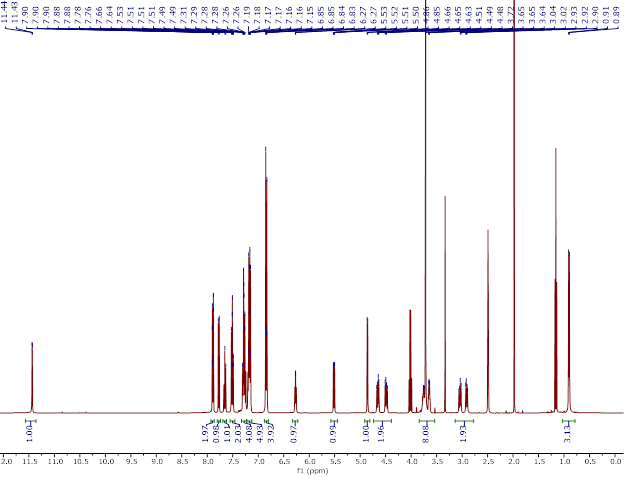


^1^H NMR (400 MHz, DMSO-*d*_6_) of **27S**


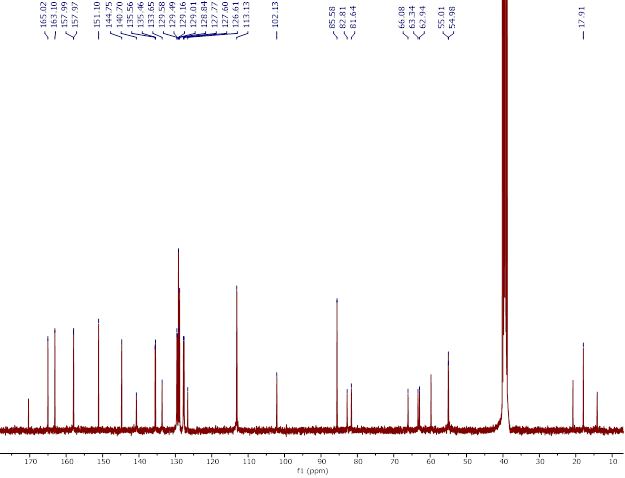


^13^C NMR (101 MHz, DMSO-*d*_6_) of **27S**


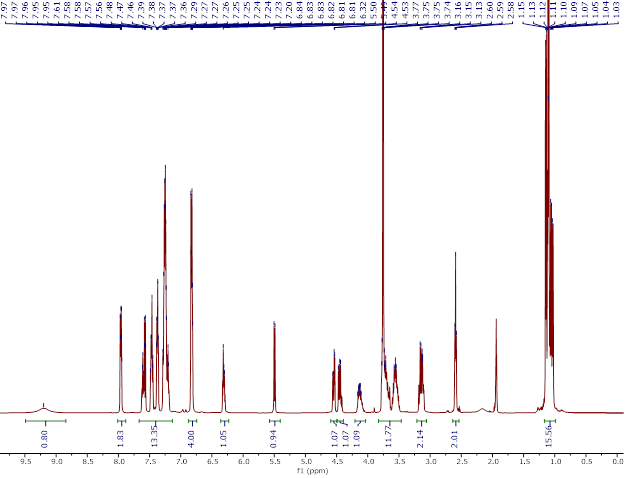


^1^H NMR (500 MHz, CD_3_CN) of **28S**


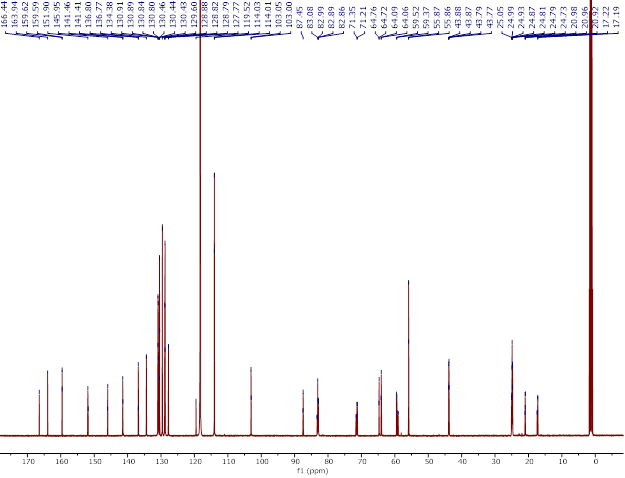


^13^C NMR (126 MHz, CD_3_CN) of **28S**


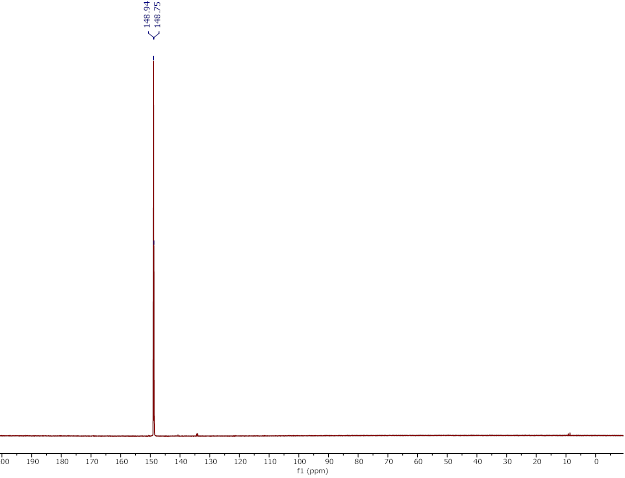


^31^P NMR (202 MHz, CD_3_CN) of **28S**


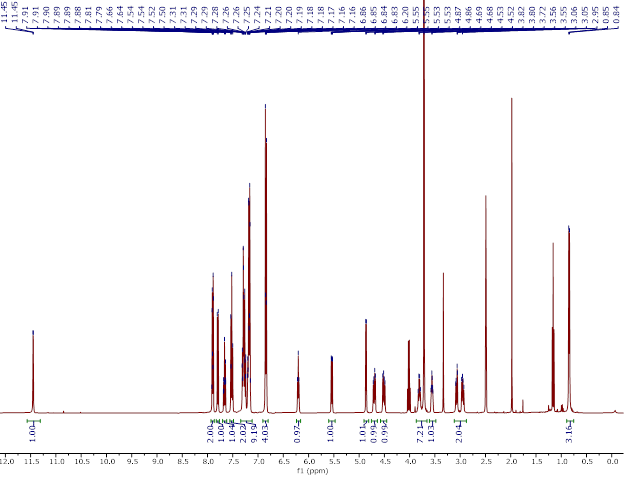


^1^H NMR (400 MHz, DMSO-*d*_6_) of **27R**


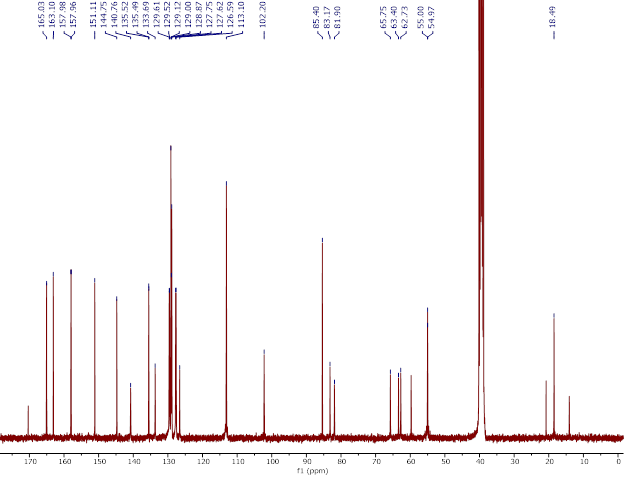


^13^C NMR (101 MHz, DMSO-*d*_6_) of **27R**

**
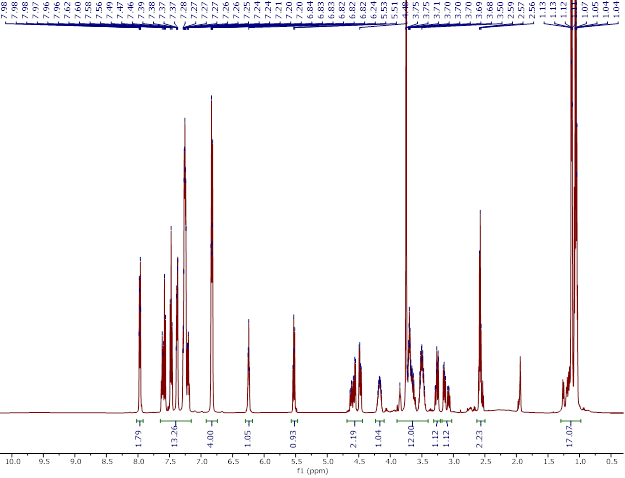
**^1^H NMR (500 MHz, CD_3_CN) of **28R**


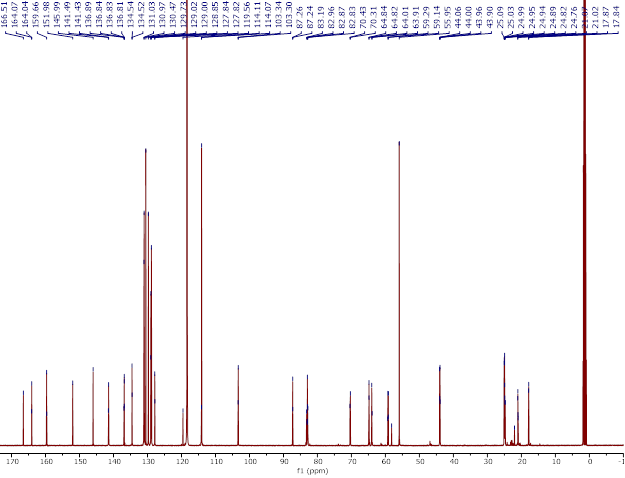


^13^C NMR (126 MHz, CD_3_CN) of **28R**


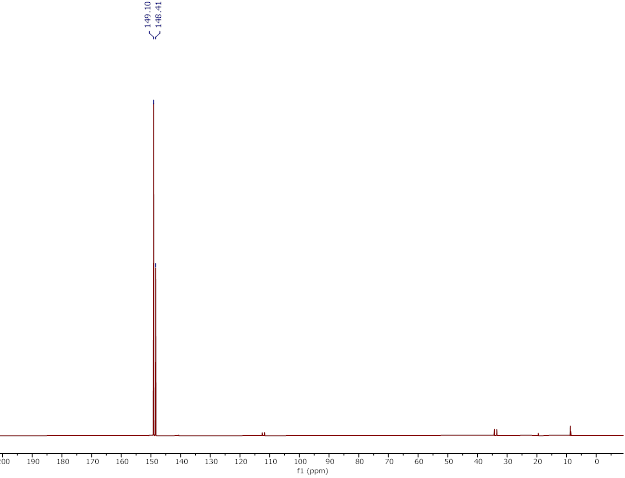


^31^P NMR (202 MHz, CD_3_CN) of **28R**


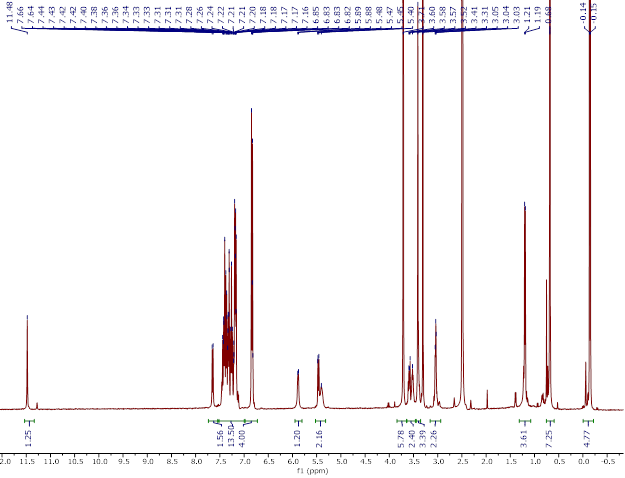


^1^H NMR (400 MHz, DMSO-*d*_6_) of **29S**


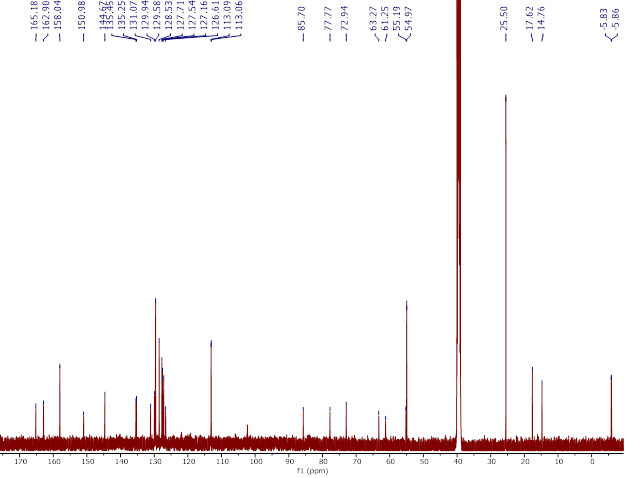


^13^C NMR (126 MHz, DMSO-*d*_6_) of **29S**


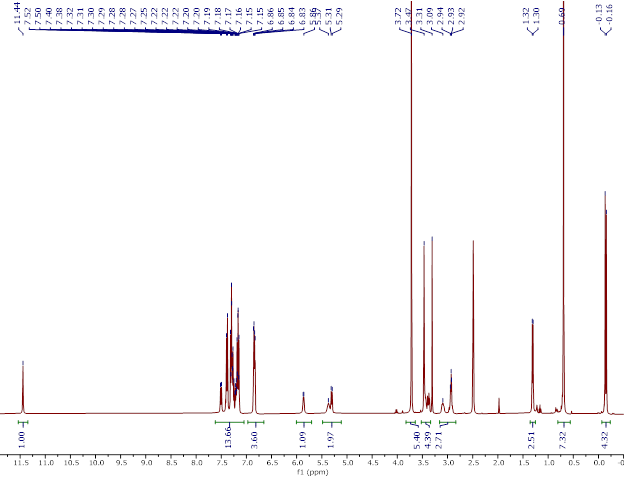


^1^H NMR (400 MHz, DMSO-*d*_6_) of **30S**


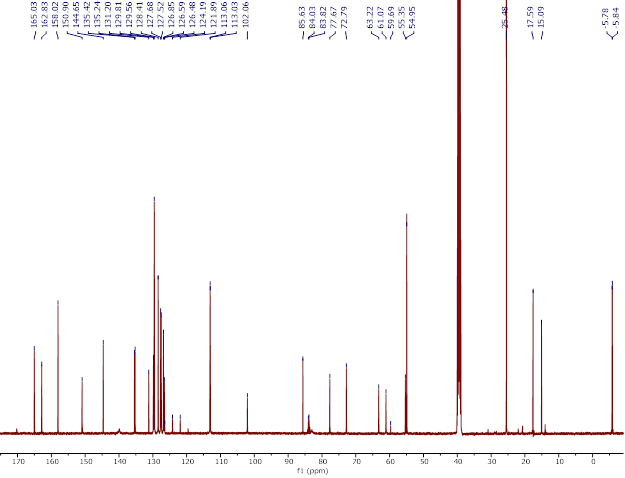


^13^C NMR (126 MHz, DMSO-*d*_6_) of **30S**


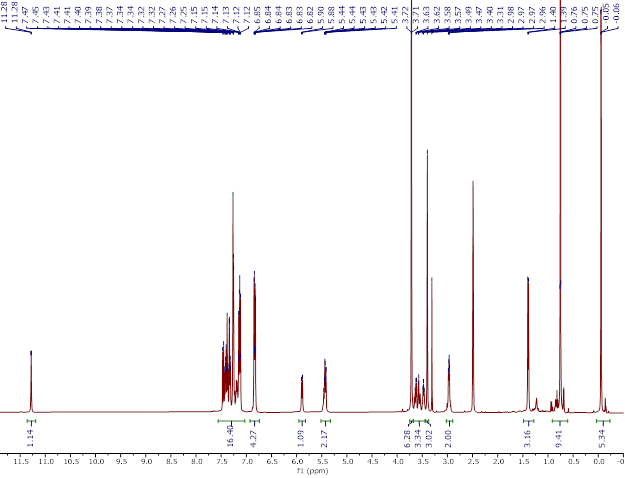


^1^H NMR (400 MHz, DMSO-*d*_6_) of **31R**


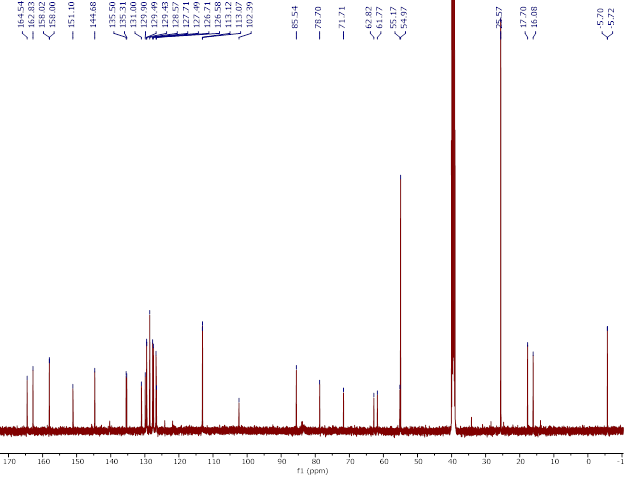


^13^C NMR (126 MHz, DMSO-*d*_6_) of **31R**


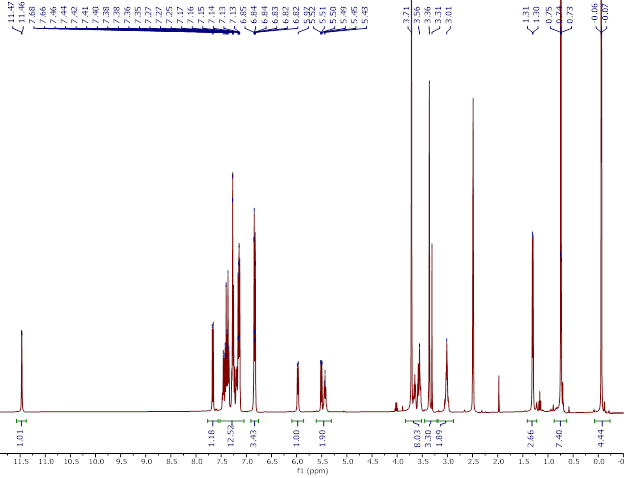


^1^H NMR (400 MHz, DMSO-*d*_6_) of **32R**


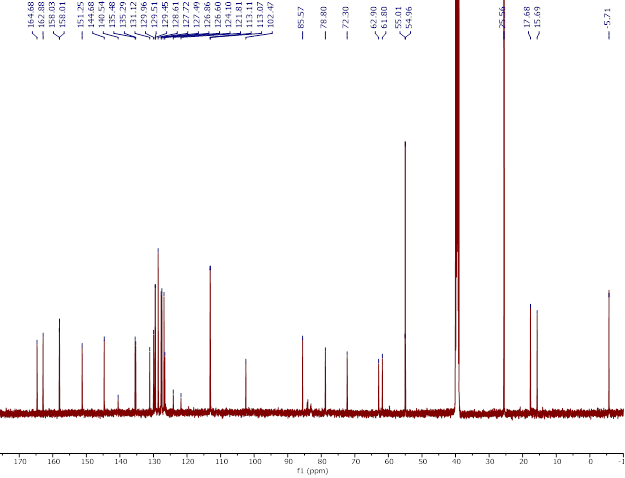


^13^C NMR (126 MHz, DMSO-*d*_6_) of **32R**
